# Protease Nexin I is a feedback regulator of EGF/PKC/MAPK/EGR1 signaling in breast cancer cells metastasis and stemness

**DOI:** 10.1038/s41419-019-1882-9

**Published:** 2019-09-09

**Authors:** Tingting Tang, Qinhua Zhu, Xinping Li, Gaole Zhu, Siwei Deng, Yingshan Wang, Lingyu Ni, Xinyuan Chen, Yanfeng Zhang, Tiansong Xia, Ke Zen, Yi Pan, Liang Jin

**Affiliations:** 1 State Key Laboratory of Natural Medicines, Jiangsu Key Laboratory of Druggability of Biopharmaceuticals, School of Life Science and Technology, China Pharmaceutical University, 24 Tongjiaxiang, Nanjing, Jiangsu province PR China; 2 Department of Breast Surgery, Breast Disease Center of Jiangsu Province, First Affiliated Hospital of Nanjing Medical University, 300 Guangzhou Road, Nanjing, Jiangsu province PR China; 3 Jiangsu Engineering Research Center for microRNA Biology and Biotechnology, State Key Laboratory of Pharmaceutical Biotechnology, School of Life Sciences, Nanjing University, 22 Hankou Road, Nanjing, Jiangsu province China

**Keywords:** Breast cancer, Cancer stem cells

## Abstract

Breast cancer is the most prevalent cancer in women worldwide, which remains incurable once metastatic. Breast cancer stem cells (BCSCs) are a small subset of breast cancer cells, which are the radical cause of drug resistance, tumor relapse, and metastasis in breast cancer. The extracellular serine protease inhibitor serpinE2, also named protease nexin-1 (PN-1), contributes to enhanced metastasis of cancer cells mainly by remodeling the tumor matrix. In this study, we found that PN-1 was up-regulated in breast cancer, which promoted cell invasion, migration and stemness. Furthermore, by using specific inhibitors, we discovered that epidermal growth factor (EGF) up-regulated PN-1 in breast cancer cells through cascade activation of epidermal growth factor receptor (EGFR) to the activation of protein kinase Cδ (PKCδ), mitogen-activated protein kinase (MEK) and extracellular signal-related kinase (ERK), which finally led to the up-regulation of early growth response protein 1 (EGR1). Moreover, EGF signaling was further activated as a feedback of PN-1 up-regulation through PN-1 blocking HtrA1. Taken together, our findings revealed a novel signaling axis that up-regulated PN-1 expression in breast cancer cells, and the new mechanism of PN-1-promoted breast cancer metastasis, which may provide new insights into identifying novel therapeutic targets for breast cancer.

## Introduction

Breast cancer is the most commonly diagnosed cancer and the leading cause of cancer-related death among females^1^. Tumor metastasis caused the majority of the deaths from breast cancer instead of the primary tumor^2,3^. Accumulating evidence indicated that the recurrent and distant metastatic tumors are related to breast cancer stem cells (BCSCs), which contribute to the development and overall aggressiveness of the recurrent or metastatic lesions. Therefore, targeting BCSCs might be a novel approach to achieve a breakthrough in the prevention of breast cancer metastasis^4,5^.

The extracellular serine protease inhibitor serpinE2, or protease nexin-1 (PN-1; a 45–50 kDa glycoprotein), plays key roles in thrombosis, cell-matrix reconstruction, and other cell life activities through inhibiting the proteolytic activity of serine proteases by covalently binding to and blocking proteases through an exposed reactive center loop (RCL)^6^. Recent studies showed that increased PN-1 in many cancer types, including breast cancer^7–11^, was associated with enhanced tumor metastasis. Also, PN-1 was reported to predict poor outcome in breast cancer patients with estrogen receptor alpha (ER-α)-negative tumors^12^. Breast tumor-bearing patients with elevated PN-1 levels also showed a higher probability of developing lung metastasis^13^. Although the mechanisms of PN-1 influencing the metastatic and invasive progression of tumors are partially elucidated, such as by remodeling the tumor matrix, polarizing tumor-associated macrophages and promoting angiogenesis^7–9^, the detailed mechanisms in breast cancer, however, still need further investigation.

In the present study, by performing RNA sequencing in MCF-7 cells and MCF-7 spheroid cells enriched in BCSCs^14^, we found PN-1 was the highest up-regulated gene in MCF-7 spheroid cells compared with MCF-7 cells, which was also discovered in the aggressive breast cancer cell line, MDA-MB-231 cells and human breast cancer tissues. Subsequently, we showed that PN-1 could promote breast cancer cell migration, invasion and stemness in vitro. Regarding the regulatory mechanisms of PN-1 expression in breast cancer cells, we elucidated that epidermal growth factor (EGF), could induce PN-1 up-regulation through the activation of EGFR/PKCδ/MEK/ERK signaling pathway in breast cancer cells, resulting in the up-regulation of EGR1, a newly identified PN-1 transcriptional factor (TF). Finally, our data suggested that PN1 was a positive-feedback regulator of EGF signaling in breast cancer cells, which could block HtrA1 and prevent EGF cleavage, and EGF could then stimulate the metastatic spread of mammary tumors through induction of PN-1.

In conclusion, we identified a novel signaling pathway of EGF-mediated up-regulation of PN-1 in breast cancer cells, which requires cascade activation of EGFR/PKCδ/MEK/ERK/EGR1, which was crucial for breast tumor metastasis and BCSC stemness. Our findings would potentially provide novel therapeutic targets for the prevention and treatment of breast cancer metastasis.

## Materials and methods

### Cell line and monolayer culture

Human breast cancer cell lines, T47D, MCF-7, MDA-MB-468, and MDA-MB-231 were purchased from the Institute of Biochemistry and Cell Biology of the Chinese Academy of Sciences (Shanghai, China). T47D, MCF-7 and MDA-MB-468 cells were maintained in DMEM medium (Gibco, USA). MDA-MB-231 cells were maintained in L-15 medium (Gibco). The medium was supplemented with 10% fetal bovine serum (FBS, Gibco), 100U/ml penicillin and 100 mg/ml streptomycin (Gibco). All cells were cultured at 37 °C with 5% CO_2_ in a humidified incubator.

### 3D semi-solid spheroids culture

3000 single cells were seeded into 24-well Ultra-Low Attachment Microplates (Corning, USA) in serum-free DMEM/F12 (Invitrogen, USA), supplemented with B27 (1:50, Invitrogen, USA), 20 ng/ml EGF (Peprotech, USA), 10 ng/ml bFGF (Invitrogen), 4 μg/ml insulin (Sigma, USA) and 20% methylcellulose (Sigma, USA). Spheres were collected 7 days afterwards.

### RNA extraction and qRT-PCR analyses

Total RNA was extracted from cultured cells using the TRIzol reagent (Invitrogen) according to the manufacturer’s instructions. For qRT-PCR, a portion (2ug) RNA was reverse transcribed to cDNA using a Reverse Transcription Kit (Vazyme, China) in a final volume of 20 μL. The reaction steps were as follows: 25 °C for 10 min, 50 °C for 30 min and 85 °C for 5 min. Real-time PCR analyses were performed with SYBR Green (Vazyme). Results were normalized to the expression of GAPDH. The rest of primers were listed in Tab. S1.

### mRNA transcriptome sequencing

Three batches of control MCF-7 cells and MCF-7 spheroid cells were harvested separately. Before the mRNA transcriptome sequencing, three batches of each group of cells were pooled together and mixed, then the total RNAs were extracted. All sequencing and data analysis were conducted by Genergy Inc (Shanghai, China). The original datasets of mRNA transcriptome sequencing were provided in Tab. S2.

### Western blotting and immunofluorescence

The protein levels were quantified by western blotting analysis of cell extracts using antibodies below: anti-GAPDH (1:2000, Santa Cruz, USA), anti-PN-1, anti-E-cadherin, anti-Vimentin, anti-Snail, anti-ZEB-1, anti-MMP9, anti-Oct4, anti-Sox2, anti-Nanog, anti-EGFR, anti-ERK1/2, anti-P-ERK1/2, anti-P-PKCδ, anti-PKCδ, anti-EGR1, anti-htrA1, and anti-EGF (1:1000, Abcam, USA). ImageJ software was used for protein bands analysis. Immunofluorescence assay was performed as described elsewhere^15^, in which the monoclonal antibodies, including anti- PKCδ (1:100, Abcam) and anti-Vimentin (1:200, Abcam) antibodies were used. Cell images were captured using a laser scanning confocal microscopy (Olympus, Japan).

### Human tissue

70 breast tumor tissues were collected from patients during surgical procedures at the First Affiliated Hospital of Nanjing Medical University (Nanjing, China). All the tumor tissues were confirmed histologically. All the patients provided written consent, and the experiments were approved by the Ethics Committee of China Pharmaceutical University, (Nanjing, China). The clinical features of the patients are listed in TableS3.

### Migration and invasion assays

Wound healing assay and transwell assays with or without Matrigel (Corning) were used for migration and invasion assays according to the published methods^16–18^. Both adherent cell and spheroid cells were collected by gentle centrifugation and trypsinized to single cells in Trypsin-EDTA solution. Then the cells were suspended in serum-free medium and 1 × 10^5^ cells were added to the upper chamber for invasion assay, and migration assay without matrigel membrane. A complete medium was added to the lower chamber and incubated for another 12 h. The medium was removed and the upper side of the filter was wiped, the migrating cells on the bottom side of the filter were fixed with 4% formaldehyde and stained with crystal violet. The cell count was done under the microscope (×200).

### Plasmid construction, siRNA interference assay, and virus infection

A mammalian expression plasmid designed to especially express ORF of human PN-1 was cloned into pcDNA3.1 vector. For negative control, an empty pcDNA3.1 plasmid was used. The siRNA targeting human PN-1 was cloned into pLVX-shRNA2 vector. An empty pLVX-shRNA2 plasmid was used as their negative control. For siRNA interference assay, Lipofectamine 2000 Reagent (Invitrogen) following the manufacturer’s protocol, the siRNAs of PN-1, EGFR, ERK1/2, PKCδ, EGR1, and HtrA1 were purchased from GenePharma (Shanghai, China). For virus infection, the siRNA targeting human PN-1 was cloned into pLVX-shRNA2 vector. The overexpression plasmid or siRNA of PN-1 were transfected into MCF-7 cells using Lipofectamine 2000 (Invitrogen, USA). Total RNA or protein was isolated 48 h after transfection. Lentivirus encoding PN-1 or sh-PN-1 were imported into MCF-7 cells as previously described^15^. The clones with the stable PN-1 or sh-PN-1 expression were selected by green fluorescence protein (GFP) expression.

### Cytokines and cell stimulation

Cells were seeded into 6-well plates at the density of 2 × 10^5^ cells/well, and starved for 24 h with fresh serum-free DMEM when the cells reached 80% confluence. Then cells were stimulated with 100 ng/mL EGF for 24 h or 1 μM PMA for 12 h. For inhibitor studies, cells were pretreated with inhibitors for 4 h before EGF or PMA stimulation, and the concentration of inhibitors were as follows according to the reported lectures: 10 μM EGFR inhibitor AG1478^19^ (MedChemExpress, USA),10 μM PKC inhibitor Go6983^20^, 5 μM MEK inhibitor U0126^21^, 20 μM PI3K inhibitor LY294002^22^, 10 μM PLC inhibitor U73122^23^, 10 μM JAK inhibitor Ruxolitinib^24^, 5 μM SCH772984^25^, 10 μM BAPTA-AM^26^, 20 μM Rottlerin^27^ (Selleck, USA), 20 μM Mithramycin A^28^(APExBIO, USA). For EGF stimulation in vitro, EGF was added into culture medium at the concentration of 50 ng/ml. For in vivo metastasis experiment, different groups of transfected cells were treated with 50 ng/ml EGF for 3 h and then injected into the tail vein of immunocompromised mice. Subsequently, EGF was injected intraperitoneally at 10 μg/kg body weight every 3 days.

### Flow cytometry

To detect the BCSCs subpopulations, the following antibodies were applied: anti-CD44-APC, anti-CD24-PE, IgG1-PE, IgG1-APC (BD). Human breast specimens were mechanically dissociated and incubated with 200 U/ml Liberase Blendzyme 4 (Roche, USA) for 2 h to obtain single cell suspensions. Then cell staining and flow cytometry were performed as described previously^16^. Cells were sorted on a flow cytometer (FACSAriaII, BD, USA) and analyzed on another flow cytometer (C6, BD, USA) with BD FACS Diva software.

### Chromatin immunoprecipitation

Chromatin immunoprecipitation (ChIP) assays were performed using EZ-CHIP KIT (Millipore, USA), according to the manufacturer’s instructions. Briefly, the cultured MCF-7 cells were treated with 1% formaldehyde and incubated for 10 min to generate cross-links of protein-DNA. Cell lysates were then sonicated to generate chromatin fragments of 200–300 bp and immunoprecipitated with anti-EGR1 pAb (Abcam) or normal rabbit IgG as control. The primer sequences used for ChIP were listed in Table S4. DNAs eluted from the ChIP assay were amplified by qPCR. Normal rabbit IgG was used as negative control. ChIP data were analyzed and represented as the percentage relative to the input DNA amount by the equation 2^[Input Ct- Target Ct]^ × 0.1 × 100.

### Coimmunoprecipitation assay

The cells were lysed using Western and IP lysis buffer (Beyotime Biotechnology) and incubated with 40 mL of protein-A/G Agarose beads (Millipore), 1 mg of rabbit anti-PN-1 (Santa Cruz Biotechnology), and anti-TCF-4 (Abcam) at 4 °C overnight. After washing three times with RIPA buffer, the samples were analyzed by Western blot analysis.

### Xenograft assays in nude mice

Six-week-old female BALB/c nude mice were purchased from the Model Animal Research Center at Nanjing University (Nanjing, China) and maintained under specific pathogen-free conditions at Nanjing University. The mice were randomly divided into 6 groups and were injected intravenously through the tail vein with modified MCF-7 cells infected with control lentivirus, cells infected with control lentivirus and incubated with EGF, cells infected with PN-1 lentivirus or sh-PN-1 lentivirus and incubated with EGF, or MDA-MB-231 cells infected with control lentivirus or sh-PN-1 lentivirus (1 × 10^6^ cells per mouse, six mice per group). For EGF-treated groups, MCF-7 cells were treated with 50 ng/ml EGF for 3 h after infected with lentivirus and then injected into the tail vein of immunocompromised mice. Subsequently, EGF was injected intraperitoneally at 10 μg/kg body weight every 3 days. The number of tumor nodules in lung was counted after 40 days. Each tissue was excised and embedded in paraffin for histopathological examination. All animal experiments were approved by the Ethics Committee of China Pharmaceutical University. Permit Number: 2162326.

### EGF Enzyme-linked immunosorbent assay

Quantitation of the amount of secreted EGF in the culture medium was achieved by an Enzyme-linked immunosorbent assay (ELISA) according to the manufacturer’s (R&D Systems).

### Statistical analysis

All experimental data were expressed as the mean and standard deviation (mean ± SD). Statistical analysis was performed using student independent *t*-test via GraphPad Prism 5 software between experimental groups. The level of significance was set at *P* < 0.05(*).

## Results

### PN-1 is up-regulated in breast cancer and predicts poor prognosis of breast cancer patients

**Fig. 1:**
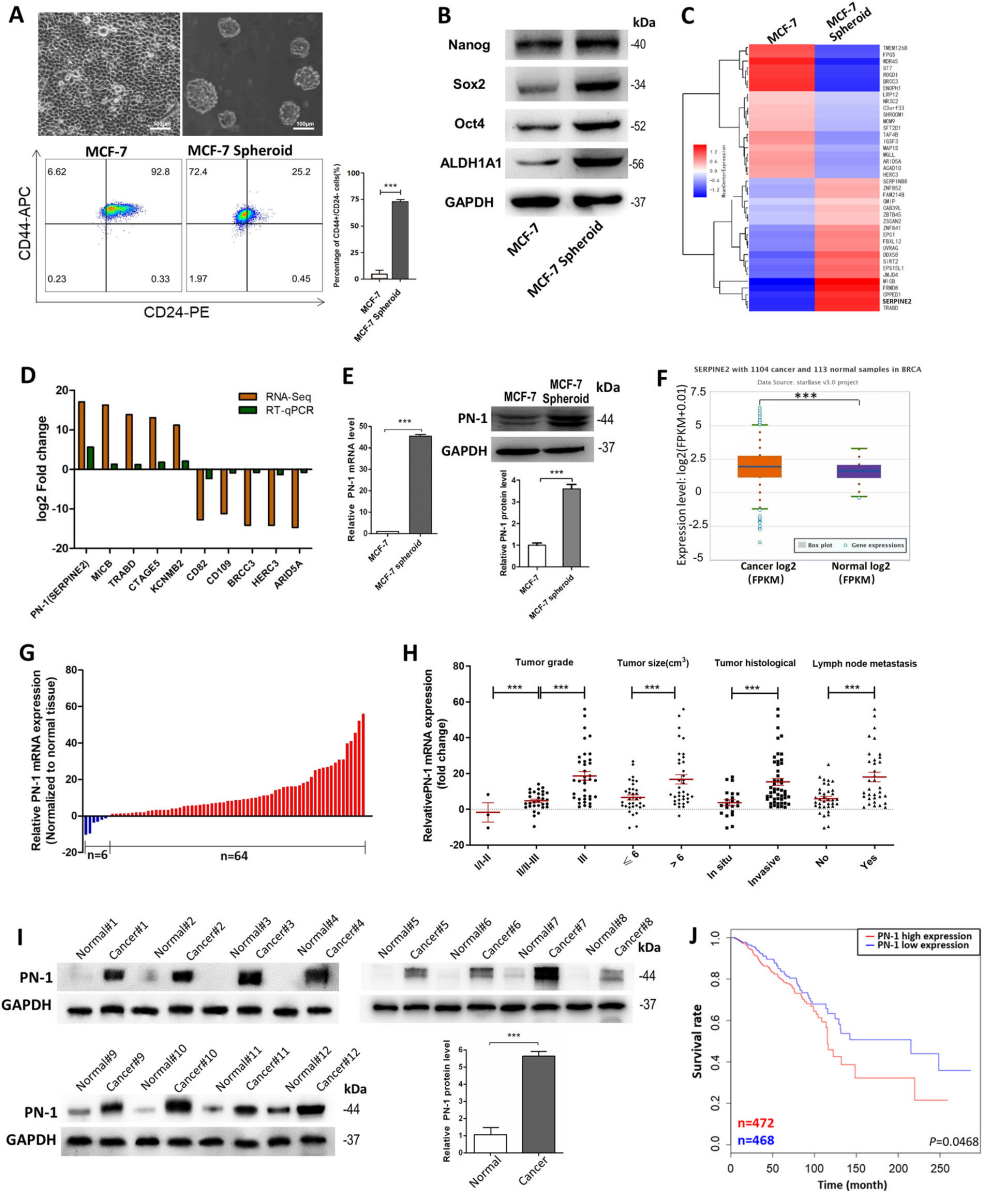
PN-1 is up-regulated in breast cancer cells. **a** Bright field images (upper) and CD44+/CD24− population percentage (lower) of MCF-7 cells formation under monolayer culture condition and 3D culture condition (scale bar: 100 μm), respectively. **b** Protein levels of BCSC surface markers and pluripotency-maintaining markers in MCF-7 parental and spheroid cells. **c** The heat maps of top-20 up- or down-regulated mRNAs between MCF-7 cells and MCF-7 spheroid cells. Each group contains three batches of individual samples, which were pooled and mixed. **d** Verification of the top-5 up-regulated and down-regulated mRNAs by qRT-PCR in MCF-7 spheroid cells compared with MCF-7 cells. **e** PN-1 mRNA(left) and protein(right) levels in MCF-7 cells and MCF-7 spheroid cells. **f** Expression levels of PN-1 in 1104 breast cancer tissues and 103 normal breast tissues in starBase public database from TCGA project (*P* < 0.05). **g** PN-1 mRNA levels were detected in 70 pairs of human breast cancer tissues and corresponding distal non-cancerous tissues by qRT-PCR. **h** PN-1 mRNA levels in breast cancer patients with different tumor grades, sizes and histological, and in patients with different tumor size, with (indicated with “yes”) or without (indicated with “no”) lymph node metastasis. **i** PN-1 protein levels were detected in 12 pairs of human breast cancer tissues (indicated by “Cancer # number of patient”) and corresponding distal non-cancerous tissues (indicated by “Normal # number of patient”) by western blotting. **j** The TCGA database indicated that patients with high levels of PN-1 expression showed reduced survival rate compared with patients with low levels of PN-1 expression by Kaplan-Meier survival analysis. (*P* *<* 0.05, log-rank test). ***P < 0.005.

We cultured MCF-7 spheroid cells by using the 3D semi-solid system^14^, in which the cells grew into homogeneous non-adherent spheroids, which were enriched in CD44+/CD24− MCF-7 cells (BCSCs) (Fig. 1a) and had higher expression of stem cell markers, such as aldehyde dehydrogenase 1 family member A1 (ALDH1A1), SRY-box 2 (SOX2), octamer-binding transcription factor 4 (OCT4), and Nanog (Fig. 1b). Then the mRNA transcriptome sequencing was performed with MCF-7 cells and MCF-7 spheroid cells to help identify the crucial genes involved in BCSCs properties^14^. In all, 588 mRNAs that were significantly changed in MCF-7 spheroids cells compared with MCF-7 cells (Table S2). Figure 1c shows the top 20 up-regulated and down-regulated mRNAs.

We first verified the top five up-regulated or down-regulated mRNAs. Among them, PN-1 changed the most (Fig. 1d), and its protein level was also up-regulated in MCF-7 spheroid cells (Fig. 1e). Furthermore, the up-regulation of PN-1 mRNA were demonstrated in 1104 breast cancer tissues and 103 normal breast tissues (StarBase database; Fig. 1f), and in 70-paired breast cancer tissues by qPCR (Fig. 1g). We further divided the 70 samples into high (above the median, *n* = 35) and low (below the median, *n* = 35) PN-1 expression groups, and explored the correlation between PN-1 expression and the clinicopathological factors, and found the PN-1 mRNA level was positively associated with tumor grade, tumor size, tumor histological, and lymph node metastasis (Table S4; Fig. 1h). The PN-1 protein levels were also increased in 12-paired breast cancer tissues compared with their corresponding distal non-cancerous tissues (Fig. 1i). We further evaluated the correlation between PN-1 expression and clinical outcomes (TCGA database), and found PN-1 high expression predicts a poor prognosis in breast cancer patients (*P* < 0.05; Fig. 1j). Taken together, these data confirmed that PN-1 is increased in breast cancer and could be used as an independent prognostic factor.

### PN-1 promotes breast cancer cell migration, invasion and stemness in vitro

**Fig. 2:**
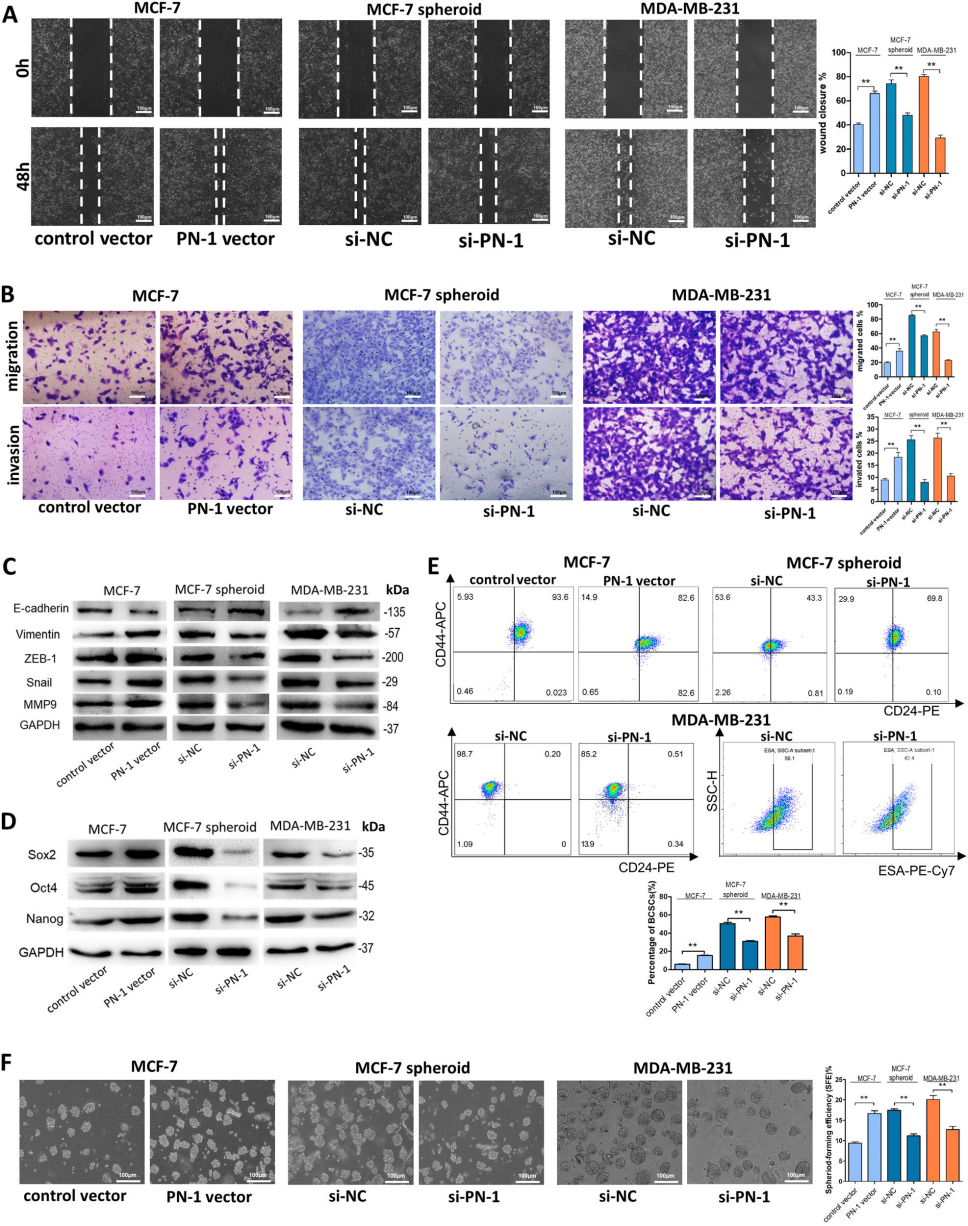
PN-1 promotes breast cancer cells migration, invasion and stemness *in vitro*. **a**, **b** Representative images and quantitative analysis of migration and invasion of MCF-7 cells transfected with control vector or PN-1 vector, MCF-7 spheroid cells transfected with si-NC or si-PN-1, and MDA-MB-231 cells transfected with si-NC or si-PN-1 detected by wound healing assay (scale bar: 100 μm) (**a**), transwell migration assay and transwell invasion assay (scale bar: 100 μm) (**b**). **c** E-cadherin, Vimentin, ZEB-1, Snail, and MMP-9 protein levels in MCF-7 cells transfected with control vector or PN-1 vector, MCF-7 spheroid cells transfected with si-NC or si-PN-1, and MDA-MB-231 cells transfected with si-NC or si-PN-1. **d** Sox2, Oct4, and Nanog protein levels in MCF-7 cells transfected with control vector or PN-1 vector, MCF-7 spheroid cells transfected with si-NC or si-PN-1, and MDA-MB-231 cells transfected with si-NC or si-PN-1. **e** The percentage of CD44+/CD24− population in MCF-7 cells transfected with control vector or PN-1 vector, MCF-7 spheroid cells transfected with si-NC or si-PN-1, and MDA-MB-231 cells transfected with si-NC or si-PN-1. **f** Sphere-formation efficiency of MCF-7 cells transfected with control vector or PN-1 vector, MCF-7 spheroid cells transfected with si-NC or si-PN-1, and MDA-MB-231 cells transfected with si-NC or si-PN-1. (scale bar: 100 μm) **P < 0.01, ***P < 0.005.

We then explored the role of PN-1 in breast cancer progress. The wound healing and transwell assays showed that the migration and invasion capacities of MCF-7 cells was significantly enhanced by overexpression of PN-1, while the migration and invasion capacities of MCF-7 spheroid cells and MDA-MB-231 cells was remarkably reduced by depleting PN-1 (Fig. 2a, b). As an extracellular matrix-secreted component, PN-1 was reported to play an important role in tumor cell and stroma interaction in breast cancer by up-regulating matrix metalloproteinase (MMP)-9^13^. We therefore examined the MMP-9 and other epithelial-mesenchymal transition (EMT)-related proteins in PN-1 overexpressed MCF-7 cells and PN-1 knocked-down MCF-7 spheroid cells and MDA-MB-231 cells. We found that the PN-1-overexpressed MCF-7 cells significantly underwent EMT process, whereas knockdown of PN-1 inhibited the EMT process in MCF-7 spheroid cells and MDA-MB-231 cells (Fig. 2c).

We also studied the effects of PN-1 on breast cancer cell stemness. Nanog, Sox-2, OCT4, ALDH1A1 and the percentage of CD44+/CD24− (MCF-7) or CD44+/CD24−/ESA+ (MDA-MB-231) cells were increased in PN-1-overexpressed MCF-7 cells while decreased in PN-1-knocked-down MCF-7 spheroid cells and MDA-MB-231 cells (Fig. 2e). Meanwhile, the PN-1-overexpressed MCF-7 cells showed higher sphere-formation abilities, which was suppressed in PN-1-knocked-down MCF-7 spheroid cells and MDA-MB-231 cells (Fig. 2f).

We further compared the proliferation and drug resistance capacities of PN-1. Results showed that PN-1 only slightly affected the proliferation rate and paclitaxel-resistance of breast cancer cells (Fig. S2A–C). The above results indicated that PN-1 mainly promote migration, invasion and stemness of breast cancer cells and BCSCs rather than proliferation and drug resistance.

### EGF up-regulates PN-1 expression in breast cancer lines

**Fig. 3:**
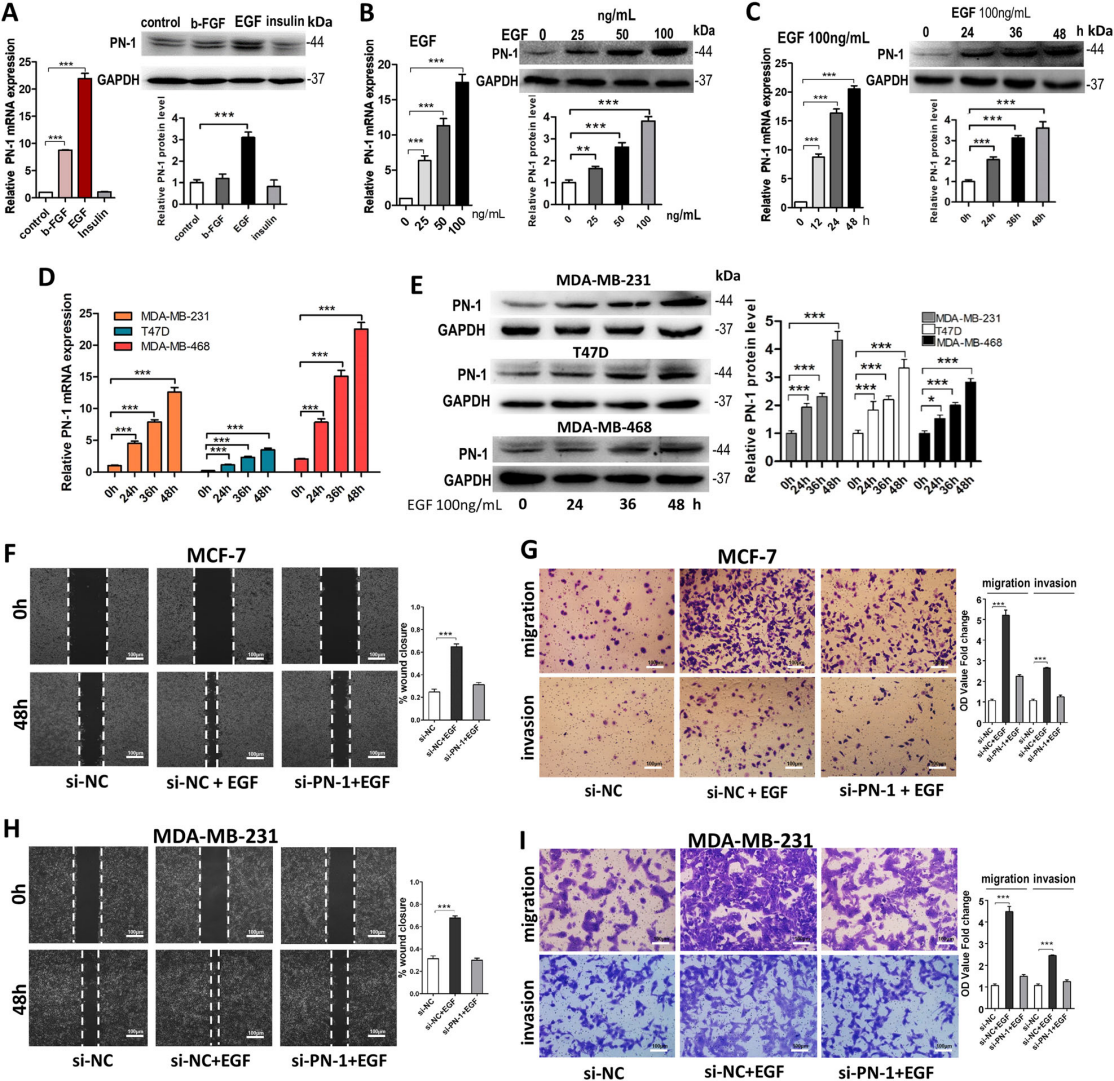
EGF up-regulates PN-1 expression in breast cancer lines. **a** PN-1 mRNA (left) and protein (right) levels in MCF-7 cells under stimulation of bFGF, EGF, and insulin. **b**, **c** PN-1 mRNA(left) and protein(right) levels in MCF-7 cells under stimulation of EGF at different doses(**b**) and for different time durations (**c**). **d**, **e** PN-1 mRNA(D) and protein (**e**) levels in MDA-MB-231 cells, T47D cells and MDA-MB-468 cells under stimulation of EGF for different time durations. **f–i** Migration and invasion of MCF-7 cells (**f**, **g**) and MDA-MB-231 (**h**, **i**) transfected with si-NC, transfected with si-NC and stimulated by EGF, or transfected with si-PN-1 and stimulated by EGF. detected by wound healing assay, transwell migration assay and transwell invasion assay. (scale bar: 100 μm) *P < 0.05, ***P < 0.005.

Next, we explored the mechanisms of PN-1 upregulation in breast cancer. EGF, b-fibroblast growth factors (b-FGF) and insulin are the three major components in our sphere-formation pelletizing system, which also exist in the tumor microenvironment^29,30^. We therefore exposed MCF-7 cells to EGF, bFGF or insulin separately and found PN-1 expression could be up-regulated by bFGF and EGF, with EGF induced most (Fig. 3a). Thus, we focused on EGF, and found EGF-induced PN-1 expression in a dose- and time-dependent manner in MCF-7, MDA-MB-231, T47D and MDA-MB-468 cells (Fig. 3b–e). Also, knockdown of PN-1 significantly suppressed the migration and invasion induced by EGF in MCF-7 and MDA-MB-231 cells (Fig. 3f–i). These results suggested that EGF could induce PN-1 up-regulation in breast cancer cells.

### EGF up-regulates PN-1 expression through binding with EGFR

**Fig. 4:**
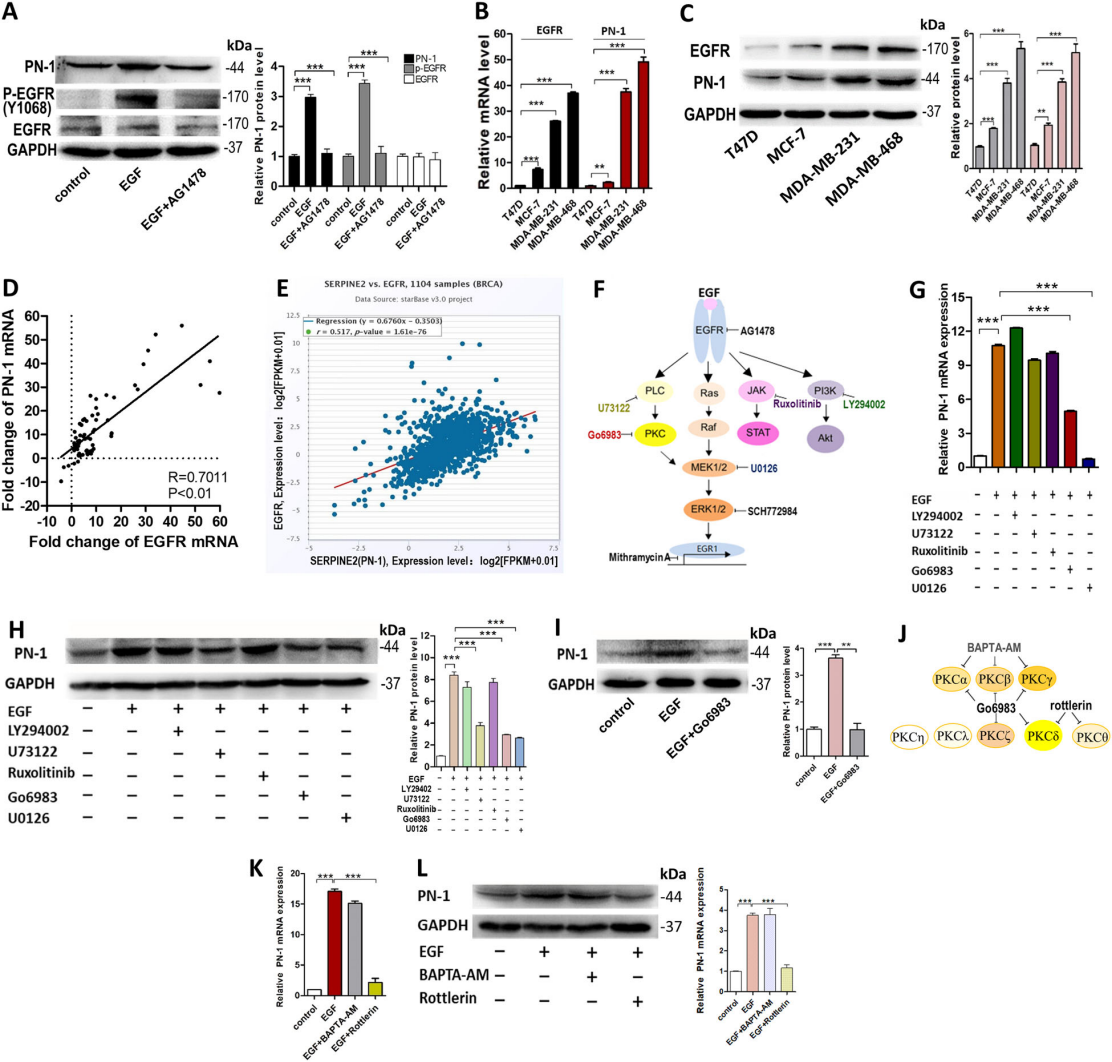
EGF induces PN-1 expression through EGFR and PKC activation. **a** PN-1, P-EGFR, and EGFR protein levels in control MCF-7 cells, MCF-7 cells treated with EGF, and MCF-7 cells treated with EGF and AG1478 (EGFR inhibitor). **b** PN-1 and EGFR mRNA levels in MCF-7 cells, MDA-MB-231 cells, T47D cells and MDA-MB-468 cells. **c** PN-1 and EGFR protein levels in MCF-7 cells, MDA-MB-231 cells, T47D cells and MDA-MB-468 cells. **d** Spearman correlation analysis of the fold change of EGFR mRNA and PN-1 mRNA in human breast cancer tissues. **e** Spearman correlation analysis of the fold change of EGFR mRNA and PN-1 mRNA in 1104 human breast cancer tissues in starBase public database from TCGA project. **f** Schematic diagram shows specific inhibitors of different proteins involved in EGF/EGFR signaling pathway. **g**, **h** PN-1 mRNA(I) and protein(J) levels in control MCF-7 cells, MCF-7 cells treated with EGF, MCF-7 cells treated with EGF and inhibitors of PI3K(LY294002), PLC(U73122), JAK(Ruxolitinib), MEK(U0126) or PKC(Go6983). **i** PN-1 protein levels in control MCF-7 cells, MCF-7 cells treated with EGF, MCF-7 cells treated with EGF and Go6983. **j** Schematic diagram shows the inhibitors of different PKC isoforms. **k**, **l** PN-1 mRNA(M) and protein(N) levels in control MCF-7 cells, MCF-7 cells treated with EGF, MCF-7 cells treated with EGF and BAPT-AM (Calcium inhibitor) or rottlerin (PKCδ and PKCθ inhibitor). ***P < 0.005.

EGFR, a receptor for EGF belonging to the ErbB family which has tyrosine kinase activity, is also known as human epidermal growth factor receptor-1 (HER1)^31^. The activated EGFR by EGF can interact with different downstream signaling molecules. Some other receptors, such as HER2 and the estrogen receptor (ER), can also be activated by EGF^32^. However, MCF-7, MDA-MB-231 and MDA-MB-468 cells are both HER2 and ER-negative. We thus determined whether EGF could up-regulate PN-1 expression through EGFR. We found AG1478, a selective EGFR tyrosine kinase inhibitor, abolished EGF-dependent inductions of PN-1 (Fig. 4a). Furthermore, we investigated the relationships between PN-1 and EGFR in breast cancer cell lines and tissues. We found the trend of EGFR expression was the same as PN-1 expression in cell lines (Fig. 4b, c) and breast cancer tissues (Fig. S3A, B). The positive correlation between EGFR and PN-1 expression levels was further illustrated in 70-paired breast cancer tissues from the hospital (Fig. 4d) and 1104 breast cancer tissues from StarBase database (Fig. 4e). We also observed a positive correlation between P-EGFR and PN-1 expression levels (Fig. S3B, C). These data indicated that the activation of EGFR is involved in PN-1 up-regulation.

### EGF up-regulates PN-1 expression through PKCδ activation

EGFR can activate many downstream kinase cascades, including PI3K/Akt, MAPK/MEK/ERK, JAK/STAT, PLCγ, and PKC pathways^33^. Specific chemical inhibitors of PKC (Go6983), MEK (U0126), JAK (Ruxolitinib), PI3K (LY294002), and PLCγ (U73122) were used to determine which signaling pathway was involved in EGF-induced PN-1 up-regulation (Fig. 4f). We found U73122, Go6983 and U0126 significantly suppressed EGF-induced PN-1 up-regulation (Fig. 4g, h), and Go6983, a ubiquitous PKC inhibitor that can inhibit PKCα/β/γ/δ/ζ, could lead to a significantly inhibited EGF-induced PN-1 up-regulation (Fig. 4i), which suggested that PLCγ/PKC pathway could be involved in PN-1 up-regulation in breast cancer cells.

We next explored which specific PKC isoform (including PKCα, PKCβ, PKCγ, PKCδ, PKCθ, PKCη, PKCζ, and PKCλ^34^) was involved in EGF-induced PN-1 up-regulation. Activation of PKCα/β/γ depends on both diacylglycerol (DAG) and calcium, while PKCδ/θ/η depend purely on DAG, and PKCζ/λ require neither calcium nor DAG^35^. We next used BAPTA-AM (calcium inhibitor) and rottlerin (a specific inhibitor for PKCδ/θ) to treat MCF-7 cells for 4 h (Fig. 4j), and found rottlerin, rather than BAPTA-AM, abolished the up-regulation (Fig. 4k, l). These data indicated that PKCδ was involved in the up-regulation. Moreover, enhanced phosphorylation of PKCδ on Thr505 was observed by using isoform-specific phospho-PKC antibody after EGF treatment for 24 h. Data showed that AG1478, Go6983, or Rottlerin could independently block the activation of PKCδ (Fig. S4A). Since the activation of PKCδ is usually accompanied by its translocation to the plasma membrane, we performed immunofluorescence assay to confirm that EGF could induce PKCδ translocation by using PMA (PKC activator), EGF and AG1478. We found both PMA and EGF treatment resulted in the translocation of PKCδ from the cytosol to the membrane, while AG1478 blocked the translocation (Fig. S4B). The results above suggested that PKCδ could be activated by EGFR activation and involved in EGF-induced PN-1 up-regulation.

### MAPK pathway is involved in the EGF-induced PN-1 up-regulation

**Fig. 5:**
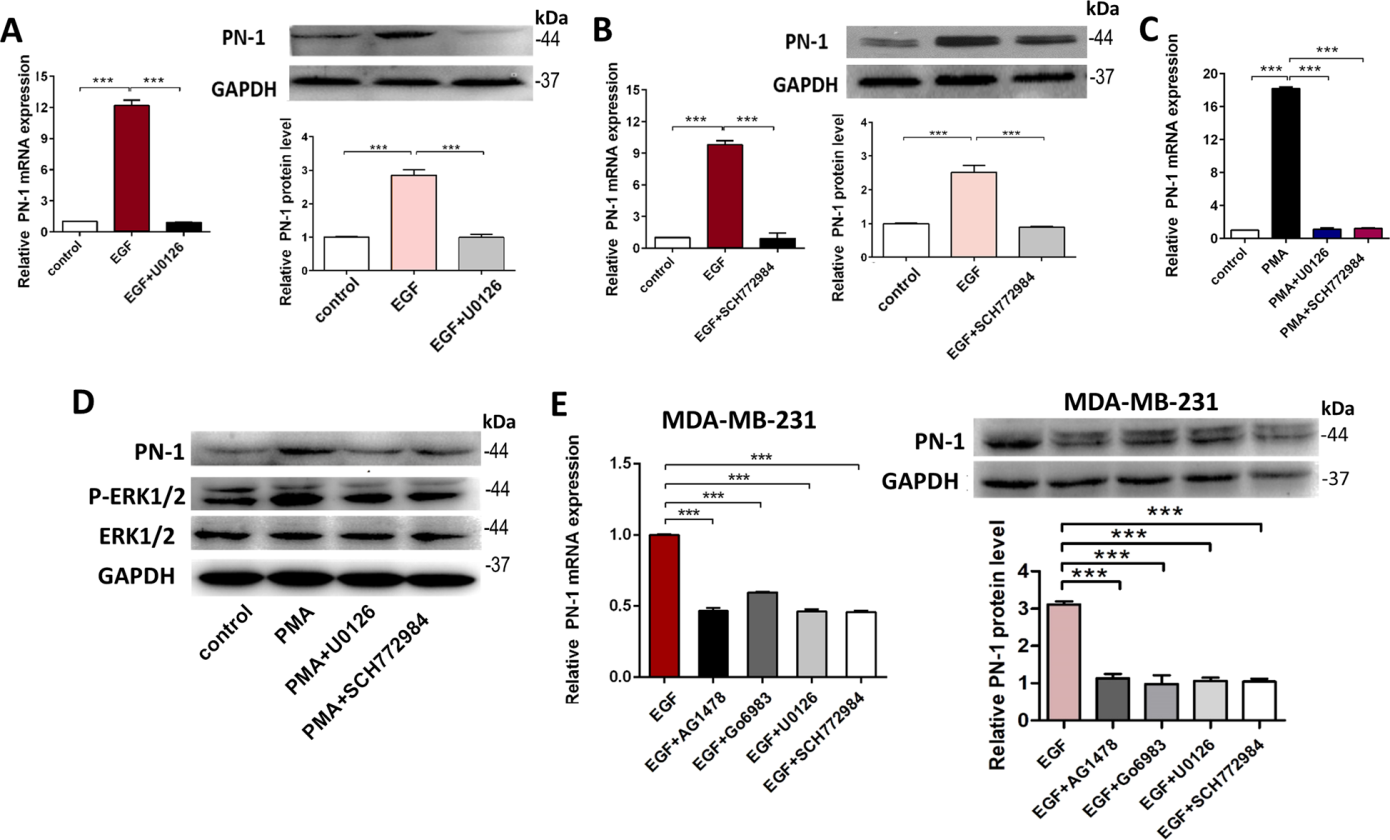
EGF-EGFR-PKC-MAPK pathway is responsible for the EGF-induced PN-1 overexpression. **a** PN-1 mRNA(left) and protein(right) levels in control MCF-7 cells, MCF-7 cells treated with EGF, and MCF-7 cells treated with EGF and U0126 (MEK inhibitor). **b** PN-1 mRNA (left) and protein (right) levels in control MCF-7 cells, MCF-7 cells treated with EGF, and MCF-7 cells treated with EGF and SCH772984 (ERK inhibitor). **c**, **d** PN-1 mRNA levels(F) and PN-1, P-ERK1/2 and ERK1/2 protein levels(G) in control MCF-7 cells, MCF-7 cells treated with PMA, and MCF-7 cells treated with PMA and U0126 or SCH772984. **e** PN-1 mRNA (left) and protein (right) levels in MDA-MB-231cells treated with EGF, MDA-MB-231 cells treated with EGF and AG1478, Go6983, U0126, or SCH772984. ***P < 0.005.

We further determined whether MAPK pathway is downstream of PKC involved in the EGF-induced PN-1 up-regulation. Firstly, we verified that 5 μM U0126 could block the EGF-induced PN-1 overexpression (Fig. 5a), and SCH772984 (ERK inhibitor) displayed the same trend in the alteration of EGF-induced PN-1 (Fig. 5b). The P-ERK was detected after EGF stimulation, which was increased in a time- and dose-dependent manner (Fig. S4C, D), and inhibited by AG1478 (Fig. S4E). These data indicated that the MAPK pathway was involved in the EGF-induced PN-1 overexpression.

Furthermore, we used PMA to certify whether PKC is upstream of the MAPK pathway. PKC activation induced PN-1 and P-ERK up-regulation in MCF-7 cells were abolished by treating with U0126 or SCH772984 for 4 h (Fig. 5c, d). Besides, 20 μM Go6983 abolished EGF-induced ERK activation (Fig. S4F), which further indicated that PKC was the upstream kinase of MAPK pathway involved in the PN-1 up-regulation. Moreover, in MDA-MB-231 cells, PN-1 overexpression could also be blocked by AG1478, Go6983, U0126 and SCH772984 (Fig. 5e). The above results demonstrated that the EGFR-PKC-MAPK pathway could contribute to the EGF-induced PN-1 up-regulation in breast cancer cells.

### Transcription factor EGR1 is involved in EGF-activated PN-1 up-regulation by binding to the PN-1 promoter

**Fig. 6:**
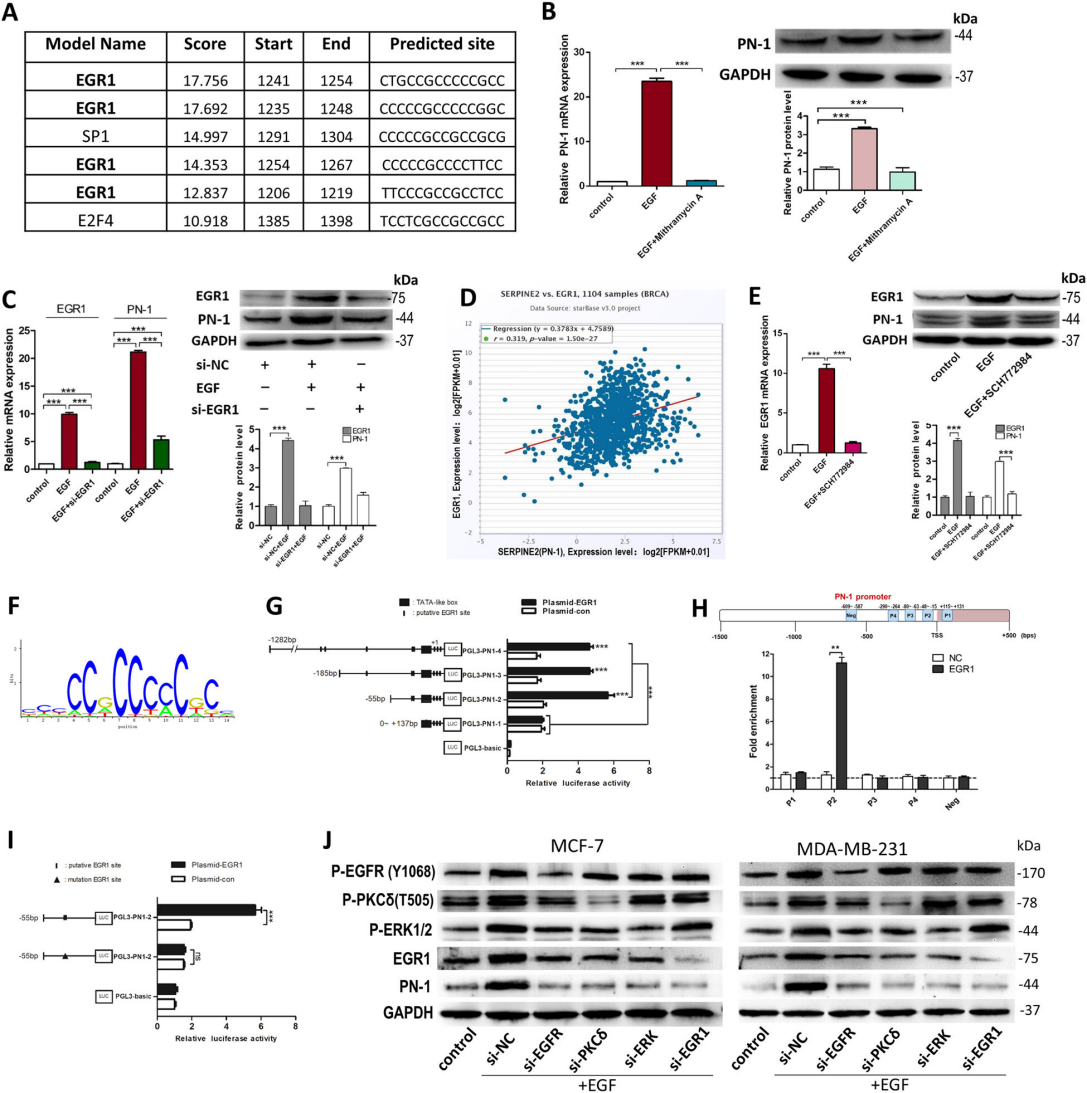
Transcription factor EGR1 was involved in EGF-activated PN-1 up-regulation. **a** Predicted binding sites of top-3 ranked transcription factors in PN-1 promoter region by JASPAR database. **b** PN-1 mRNA(left) and protein(right) levels in control MCF-7 cells, MCF-7 cells treated with EGF, MCF-7 cells treated with EGF and mithramycin A (GC-rich-binding agent). **c** EGR1 and PN-1 mRNA(left) and protein(right) levels in MCF-7 cells transfected with si-NC, transfected with si-NC and stimulated by EGF, or transfected with si-EGR1 and stimulated by EGF. **d** Spearman correlation analysis of the fold change of EGR1 mRNA and PN-1 mRNA in 1104 human breast cancer tissues in starBase public database from TCGA project. **e** EGR1 mRNA(left), EGR1 and PN-1 protein(right) levels in in control MCF-7 cells, MCF-7 cells treated with EGF, and MCF-7 cells treated with EGF and SCH772984. **f** Sequence logo of EGR1 from JASPAR database. **g** The verification of EGR1 binding to the promoter region of PN-1 by luciferase reporter assay. Plasmid-con, Plasmid-EGR1, PGL3-basic and PGL3-PN-1 vectors which included different regions of PN-1 promoter were transfected into MCF-7 cells for 48 h, then luciferase was checked. **h** Putative EGR1-binding sites on the promoter region of PN-1 by JASPAR (upper) and the enrichment of EGR1 on PN-1 promoter relative to IgG in MCF-7 cells detected by ChIP assay (lower). A random region (Neg) without DNA binding elements (DBEs) of EGR1 served as a negative control. **i** Mutagenesis in the putative binding site (−49~−16 bp fragment of the PN-1 promoter) abrogated the induction activity of EGR1 in the MCF-7 cells. **j** P-EGFR, PKCδ, P-ERK1/2, EGR1, and PN-1 protein levels in control MCF-7 cells (left) or MDA-MB-231 cells (right), MCF-7 cells (left) or MDA-MB-231 cells (right) transfected with si-NC, si-EGFR, si- PKCδ, si-ERK or si-EGR1 and treated with EGF. **P < 0.01, ***P < 0.005.

To determine the TF of PN-1 activated by EGF/PKC/ERK signaling axis, JASPAR database was used to predict the TFs binding to the PN-1 promoter (Fig. 6a), and we found their predicted binding sites are all in the high-GC-content regions, and EGR1 has the highest potential. EGF-induced PN-1 overexpression was completely inhibited by mithramycin A (a GC-rich binding agent; Fig. 6b), indicating the potential TF could interact with the high-GC-content regions in the PN-1 promoter. Furthermore, we observed that EGF induced the up-regulation of EGR1 (Fig. 6c) but not SP1 or EF4 (data note shown), and si-EGR1 significantly inhibited the EGF-induced PN-1 up-regulation in MCF-7 cells. The positive correlation between EGR1 and PN-1 protein levels was supported by using Spearman correlation analysis in 1104 breast cancer tissues (Fig. 6d; StarBase database). Moreover, SCH772984 suppressed the EGF-induced EGR1 and PN-1 up-regulation (Fig. 6e). Also, we identified the binding sites between EGR1 and PN-1 promoter. Sequence analyses of the PN-1 promoter region showed eight putative EGR1-binding sites (Fig. 5f, g). Luciferase reporter assays showed EGR1 significantly increased the luciferase level by binding to a promoter region within 55 bp upstream of the transcription start point of PN-1 (Fig. 6g). Chromatin immunoprecipitation (ChIP) assay confirmed the −49~−16 bp fragment of the PN-1 promoter is sufficient for the binding of EGR1 (Fig. 6h, lower). Bioinformatics analysis of the promoter region of PN-1 predicted four high scores DNA binding elements (DBEs) for EGR1 (Fig. 6h, upper). Mutagenesis in the P2 site abrogated the induction activity of EGR1 in the MCF-7 cells (Fig. 6i).

Finally, we detected the protein levels of P-EGFR, P-PKCδ, P-ERK, EGR1, and PN-1 wa in EGF-treated and EGFR, PKCδ, ERK or EGR1 knocked-down MCF-7 and MDA-MB-231 cells (Fig. S1C–F and Fig. 6i). We also confirmed that inhibition of the PKC/MAPK/EGR1 signaling pathway interfered with the ability of PN-1 to modulate invasion, migration and stemness (Fig. S5). The above results demonstrated that EGF could induce PN-1 up-regulation through activation of EGF/PKC/MAPK/EGR1 signaling pathway.

### EGF-induced PN-1 up-regulation promotes breast tumor cell metastasis in mouse model

**Fig. 7:**
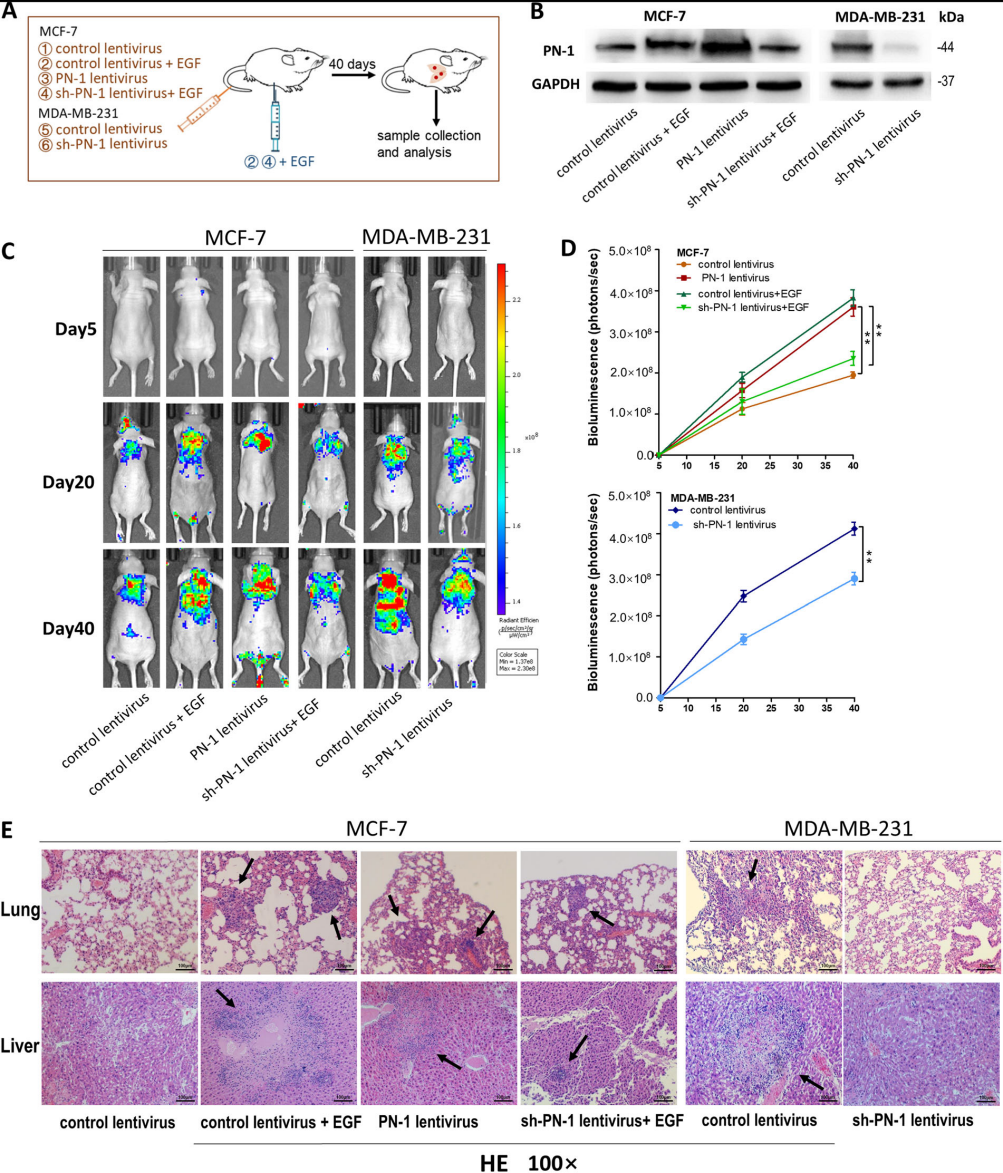
Effects of EGF-induced PN-1 on the lung colonization of breast cancer cells xenografts in mice. **a** Experimental design. Immunocompromised mice were injected through tail vein with either MCF-7 cells transfected with control lentivirus, transfected with control lentivirus and treated with EGF, transfected with PN-1 lentivirus, transfected with sh-PN-1 lentivirus and treated with EGF, or MDA-MB-231 cells transfected with control lentivirus or sh-PN-1 lentivirus. For group 2 and 4, EGF was also injected intraperitoneally at 10 μg/kg body weight every 3 days. **b** PN-1 protein levels in MCF-7 cells transfected with control lentivirus, transfected with control lentivirus and treated with EGF, transfected with PN-1 lentivirus, transfected with sh-PN-1 lentivirus and treated with EGF, and in MDA-MB-231 cells transfected with control lentivirus or sh-PN-1 lentivirus. **c** Representative BLI images of four groups. The BLI was performed on day 5, 20, and 40 after injection. The intensity of BLI is represented by the color. **d** Whole body bioluminescence (photons/second) following tail vein injection of breast cancer cells in mice. **e** Mice lungs (upper) and livers (lower) were subjected to H&E staining respectively. (Scale bar:100 μm) ***P < 0.005.

We first generated four types of modified MCF-7 cells and two types of modified MDA-MB-231 cells, and injected these cells into female nude mice (six weeks, 22–24 g) via the tail vein (Fig. 7a, b). The bioluminescent imaging (BLI) assay indicated that the GFP-labeled migrating cells were mainly distributed in the lungs and livers (Fig. 7c, d). The fluorescent intensities of lung and liver were significantly stronger in EGF-treated MCF-7 cells group and PN-1-overexpressing MCF-7 cells group while unchanged in EGF-treated and PN-1 knocked-down MCF-7 cells group. Accordingly, the fluorescent intensities were weaker in PN-1 knocked-down MDA-MB-231 cells group.

After 40 days, mice were sacrificed and the whole lung and liver tissues were harvested, and subjected to H&E staining for evaluating tumor metastasis. In mice injected with EGF-treated or PN-1-overexpressing MCF-7 cells, bigger sized tumors with clear boundaries (arrows) were found in the lungs and livers, while only small tumor masses were scattered in the lungs and livers of EGF-treated and PN-1 knocked-down MCF-7 cells group and control group. Less tumors were observed in PN-1 knocked-down MDA-MB-231 cells group than control group (Fig. 7e). These results supported the role of EGF-induced PN-1 up-regulation in promoting breast cancer cells metastasis in mice.

### PN1 is a positive-feedback regulator of EGF/ERK/EGR1 signaling through blocking HtrA1 and up-regulating extracellular EGF level

**Fig. 8:**
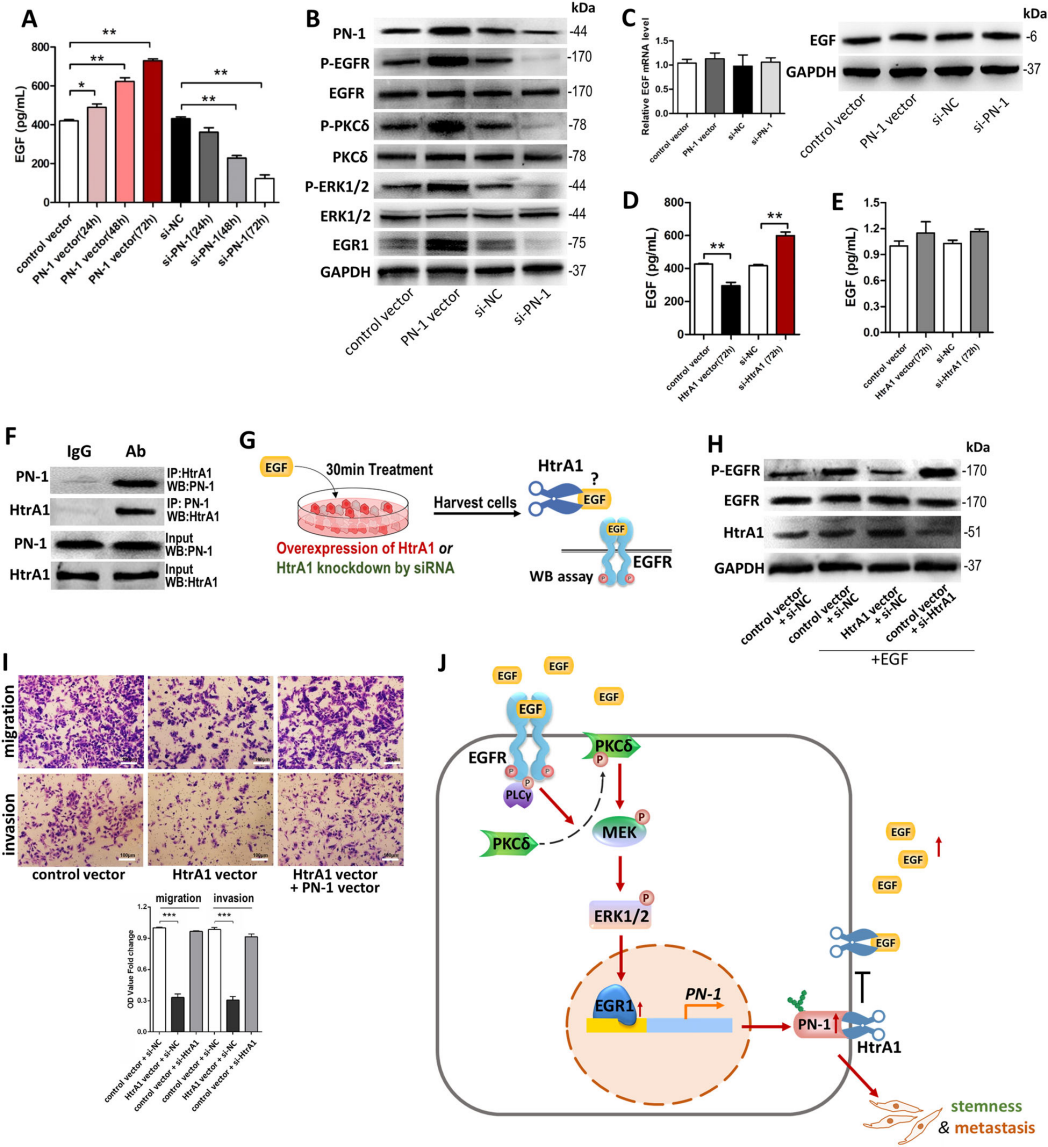
PN1 acts as a positive-feedback regulator of EGF/ERK/EGR1 signaling through binding and inhibiting HtrA1. **a** Concentration of EGF (pg/mL) in culture medium of MCF-7 cells transfected with control vector or PN-1 vector, si-NC or si-PN-1 for 24, 48 or 72 h detected by ELISA. **b** p-EGFR, EGFR, p-PKCδ, PKCδ, p-ERK1/2, ERK1/2 and EGR1 protein levels in MCF-7 cells transfected with control vector, PN-1 vector, si-NC or si-PN-1 for 48 h. **c** EGF mRNA(left) and protein(right) levels in MCF-7 cells transfected with control vector or PN-1 vector, si-NC or si-PN-1. **d** Concentration of EGF (pg/mL) in culture medium of MCF-7 cells transfected with control vector or HtrA1 vector, si-NC or si- HtrA1 for 72 h detected by ELISA. **e** EGF mRNA levels in MCF-7 cells transfected with control vector or HtrA1 vector, si-NC or si- HtrA1. **f** HtrA1 co-immunoprecipitates with PN1, and PN1 in turn co-immunoprecipitates with HtrA1 in MCF-7 cells. **g** Schematic diagram of the assay designed to assess the effect of extracellular HtrA1 on the regulation of the EGF signaling pathway. MCF-7 cells, which were transfected with control vector or PN-1 vector, si-NC or si-PN-1, were treated with the EGF for 30 min. The effect of the enzyme-substrate pair HtrA1 and EGF on the EGF signaling pathway in the extracellular region was assessed by the extent of P-EGFR. **h** P-EGFR, EGFR and HtrA1 protein levels in control MCF-7 cells and MCF-7 cells transfected with control vector or PN-1 vector, si-NC or si-PN-1, and treated with the EGF. **i** Migration and invasion of MCF-7 cells transfected with control vector, HtrA1 vector, or HtrA1 vector plus PN-1 vector detected by transwell migration assay and transwell invasion assay. **j** A working model for the regulation of PN-1 by EGF/EGFR/PKCδ/MEK/ERK/EGR1 signaling pathway in breast cancer cells. During breast tumorigenesis, EGF is up-regulated in tumor microenvironment and binds with EGFR, which leading to the activation of the downstream kinase cascades including PKCδ, MEK, and ERK, finally induces the expression of PN-1 via up-regulation of its transcription factor, EGR1. PN-1 promotes migration, invasion and stemness of breast cancer cells and further provides a positive-feedback towards the activation of EGF signals through preventing EGF cleavage by HtrA1. *P < 0.05, ** *P* < 0.01, ***P < 0.005.

ELISA results showed that more EGF had been secreted to the extracellular after overexpression of PN-1 while PN-1 knockdown decreased this level (Fig. 8a). Western blotting exhibited PN-1 activated EGFR, P-ERK1/2 and PKCδ, and up-regulated EGR1 protein levels. Reciprocally, PN-1 depletion suppressed the activation of EGF/EGFR/ERK/EGR1 signaling (Fig. 8b). The above results provided preliminary evidence for feedback regulation of EGF/EGFR/ERK/EGR1 signaling by PN-1.

Furthermore, we investigated the underlying mechanisms of PN-1 regulating EGF/EGFR signaling. Firstly, we detected that EGF levels in MCF-7 cells ware not altered by PN-1 (Fig. 8c), indicating PN-1 did not affect the endogenous EGF expression. Since PN-1 could inhibit target serine proteases, such as HtrA1, and HtrA1 was reported to play a role in the degradation and inhibition of secreted growth factors in the extracellular region^36,37^. We speculated that EGF may serve as a cleavage target for HtrA1, which could be blocked by PN-1. By detecting extracellular and cellular EGF levels, we found that HtrA1 negatively regulated extracellular EGF levels (Fig. 8d), but did not affect cellular levels (Fig. 8e). Co-IP assay showed PN1 and HtrA1 could co-immunoprecipitated with each other (Fig. 8f). We further assessed the effects of HtrA1 on the regulation of EGF signaling, MCF-7 cells, which were transfected with control vector or PN-1 vector, si-NC or si-PN-1, were treated with the EGF for 30 min. The effect of the enzyme-substrate pair HtrA1 and EGF on the EGF signaling pathway in the extracellular region was assessed by the extent of P-EGFR (Fig. 8g). We found HtrA1 overexpression abolished the EGF-induced EGFR phosphorylation while HtrA1 knockdown promoted (Fig. 8h). Furthermore, we observed HtrA1 overexpression inhibited the migration and invasion of MCF-7 cells and PN-1 overexpression abolish the inhibitory effect (Fig. 8i). Therefore, our data demonstrated that PN-1 provides a positive-feedback towards the activation of EGF signals through preventing EGF cleavage by HtrA1.

## Discussion

Tumor metastasis is the main reason for breast cancer-related mortality^38^. PN-1 is becoming increasingly recognized as an essential player in malignant progression and metastasis, which is overexpressed in the breast cancer;^13^ however, the mechanisms remain largely unclear. In this study, we suggest that PN-1 expression could be positively regulated by EGF/EGFR/PKCδ/MEK/ERK/EGR1 signaling pathway in breast cancer cells, and the study has been summarized in Fig. 8j.

Proteases and their inhibitors are key regulators for extracellular matrix remodeling, a process that contributes to metastasis. In this study, we demonstrated the PN-1 expression was elevated in BCSCs and revealed its role in BCSCs for the first time, and elevated PN-1 levels significantly reduce the overall survival rate. Overexpression of PN-1 promotes migration, invasion and stemness of breast cancer cells and BCSCs. Our results supported the role of PN-1 as an oncogene in malignant progression and metastasis of breast cancer, and suggested that PN-1 might be involved in BCSC stemness maintenance.

PN-1 has been shown to promote metastasis in various human cancers by multiple mechanisms such as by remodeling the tumor matrix and polarizing tumor-associated macrophages^39^, activating glycogen synthesis kinase 3β (GSK-3β) signaling pathway^40^, and activating P38 signaling pathways^41^. PN-1 was reported to be up-regulated by oncogenic activation of Ras, BRAF and MEK and contributes to pro-neoplastic actions of ERK signaling in colorectal cancer cells^42^. However, how PN-1 is regulated in breast cancer cells remains largely unclear. It is known that PN-1 secretion could be stimulated in normal cell lines by many different cytokines such as FGF^43^ and TGF-β^32^. Here we showed EGF had a significant effect on PN-1 up-regulation, which was once reported to up-regulate PN-1 in astrocytoma U373-MG cells^44^. PN-1 knockdown significantly suppressed the EGF-triggered enhancement in migration and invasion abilities both in vitro and in vivo, which indicated that PN-1 participated in the pro-metastasis effect of EGF. We also found the overexpression of EGFR was positively correlated with PN-1 expression in breast cancer cells and tissues. EGFR activation via EGF binding could activate various pathways that ultimately resulting in carcinogenesis^45^. Aberrant expression or activity of EGFR has been strongly linked to the etiology of breast cancer^46^.

EGFR regulates the downstream signaling pathways such as the PI3K/AKT, Ras/Raf/MEK/ERK, and PLCγ1/PKC pathways^47^. Activation of MEK/ERK has been reported to induce PN-1 genes in colorectal cancer cells^9^ and *Xenopus* embryonic cells^42^. In this study, we screened out a non-classical PKCδ/MAPK/ERK signaling pathway involved in EGF-induced PN-1 up-regulation in breast cancer cells, first provided the evidence that PN-1 could be up-regulated by EGF/EGFR/PKCδ/MEK/ERK signaling pathway. We also identified EGR1 could serve as a TF of PN-1 activated by EGF signaling pathway. The roles of EGR1 in cancer development are ambiguous since EGR1 may act as either oncogene or tumor suppressor gene in different cancer types. EGR1 promotes cell motility in various cancer cells including breast cancer^48–50^, while inhibits EMT in non-small-cell lung cancer cells and bladder cancer cells^51,52^. EGR1 expression mediates an EGF-ERK signaling cascade was reported in prostate cancer cells and breast cancer cells^53,54^, which contributes to the migration of cancer cells. Our data support the finding that EGR1 could serve as oncogene in the breast cancer and first provide the evidence that it links to EGF, ERK, EGR1, PN-1 and cell migration.

Finally, we uncovered PN-1 engaged in a positive feedforward loop that causes amplification of EGF/ERK/EGR1 signal, which might enhance the pro-metastasis effect of PN-1. PN-1 has recently been reported to stimulate ERK signaling by binding low-density lipoprotein receptor-related protein-1 receptor in mouse breast cancer 4T1 cells^13^ or transmembrane glycoprotein syndecan-1 in mouse embryonic fibroblasts cells^55^. We further investigated the underlying mechanisms of the activation of EGF signaling by PN-1 in breast cancer cells and demonstrated that PN-1 could prevent extracellular EGF proteolytic cleavage by HtrA1 through binding and blocking HtrA1. HtrA1 is a secreted enzyme that closely related to the degradation of extracellular matrix and secreted growth factors^56^. The emerging evidence has demonstrated that HtrA1 participates in the inhibition of cancer cell apoptosis, invasion and metastasis, and down-regulation of HtrA1 protein is associated with poor survival in mesothelioma, hepatocellular carcinoma and breast cancer^57–59^. Herein, we illustrated a novel mechanism of PN-1 promoting breast cancer metastasis by binding and blocking HtrA1, which could cleave extracellular EGF and suppress cancer cell EMT.

In conclusion, our results suggested that PN-1, which is up-regulated in breast cancer cells and BCSCs through EGF/PKC/MAPK/EGR1 signaling, is related to poor prognosis and could serve as a positive-feedback regulator in breast cancer cells metastasis and stemness. Hence, the EGF/EGFR/PKC/MEK/ERK/EGR1/PN1/HtrA1 signaling axis might be a potential therapeutic target for breast cancer treatment.

## Supplementary information

Supplementary Figure Legends.

Supplementary Figure S1.

Supplementary Figure S2.

Supplementary Figure S3.

Supplementary Figure S4.

Supplementary Figure S5.

Supplementary Table S1.

Supplementary Table S3.

Supplementary Table S3.

Supplementary Table S4.

## Data Availability

All data generated or analyzed during this study have been included in this published article and its supplementary information files.
