# Correction: Protease Nexin I is a feedback regulator of EGF/ PKC/MAPK/EGR1 signaling in breast cancer cells metastasis and stemness

**DOI:** 10.1038/s41419-019-2211-z

**Published:** 2020-01-06

**Authors:** Tingting Tang, Qinhua Zhu, Xinping Li, Gaole Zhu, Siwei Deng, Yingshan Wang, Lingyu Ni, Xinyuan Chen, Yanfeng Zhang, Tiansong Xia, Ke Zen, Yi Pan, Liang Jin

**Affiliations:** 1 State Key Laboratory of Natural Medicines, Jiangsu Key Laboratory of Druggability of Biopharmaceuticals, School of Life Science and Technology, China Pharmaceutical University, 24 Tongjiaxiang, Nanjing, Jiangsu Province People’s Republic of China; 2 Department of Breast Surgery, Breast Disease Center of Jiangsu Province, First Affiliated Hospital of Nanjing Medical University, 300 Guangzhou Road, Nanjing, Jiangsu Province People’s Republic of China; 3 Jiangsu Engineering Research Center for microRNA Biology and Biotechnology, State Key Laboratory of Pharmaceutical Biotechnology, School of Life Sciences, Nanjing University, 22 Hankou Road, Nanjing, Jiangsu Province People’s Republic of China

**Keywords:** Breast cancer, Cancer stem cells

**Correction to: Cell Death and Disease (2019)**

10.1038/s41419-019-1882-9, published online 9 September 2019.

Since online publication of this article, the authors noticed that there was an error in the images used to compile Figs. 2 and 7. An incorrect MCF-7 spheroid GAPDH image was used in Fig. 2c, and incorrect MCF-7 and MCF-7 spheroid flow charts were used in Fig. 2e. In addition, an incorrect image was used for the Day 20 PN-1 lentivirus BLI mouse image for Fig. 7c. The corrected images are provided below. The authors confirm that these errors do not affect the results and conclusions of the study.

**Figure 1:**
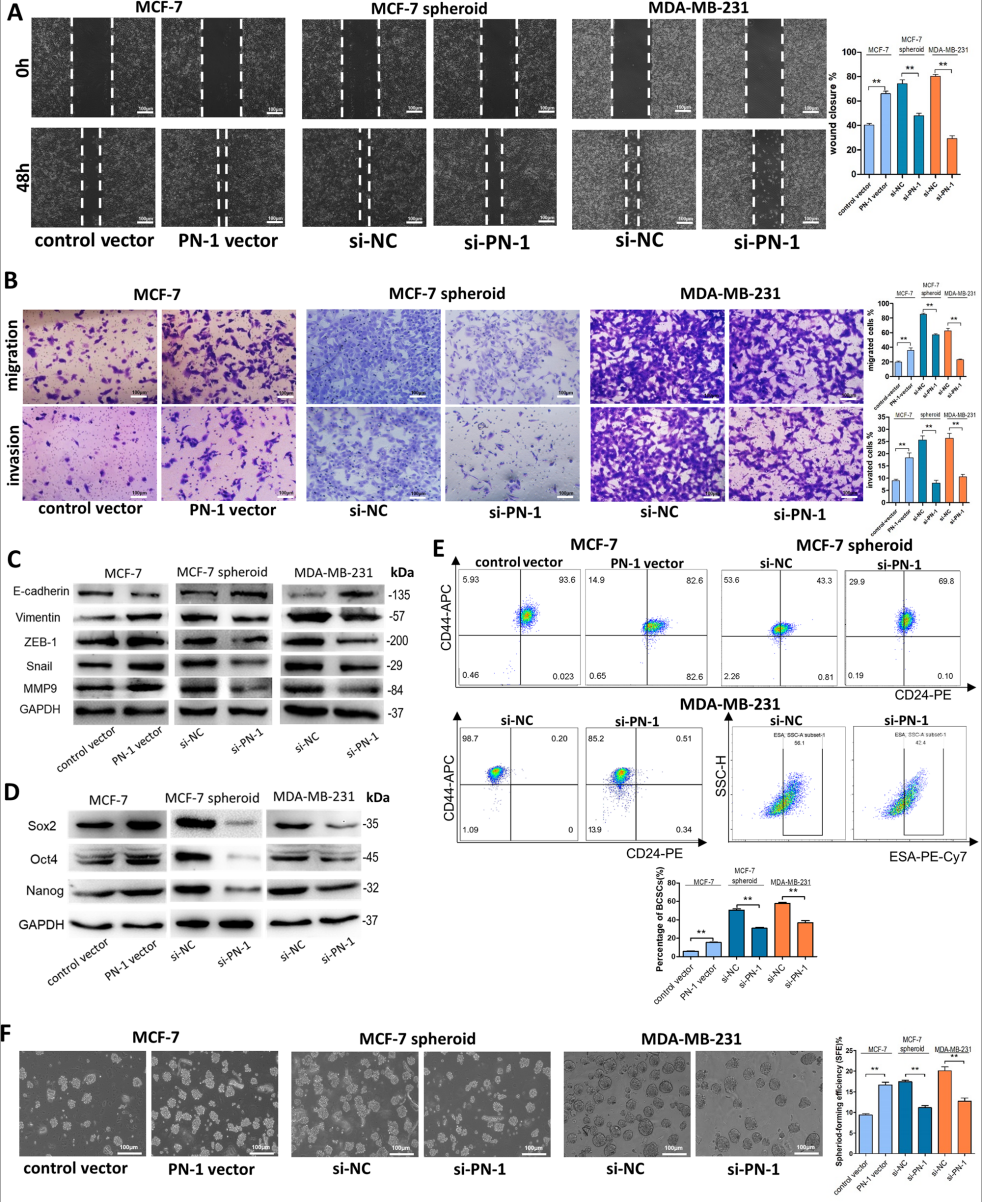
Fig. 2

**Figure 2:**
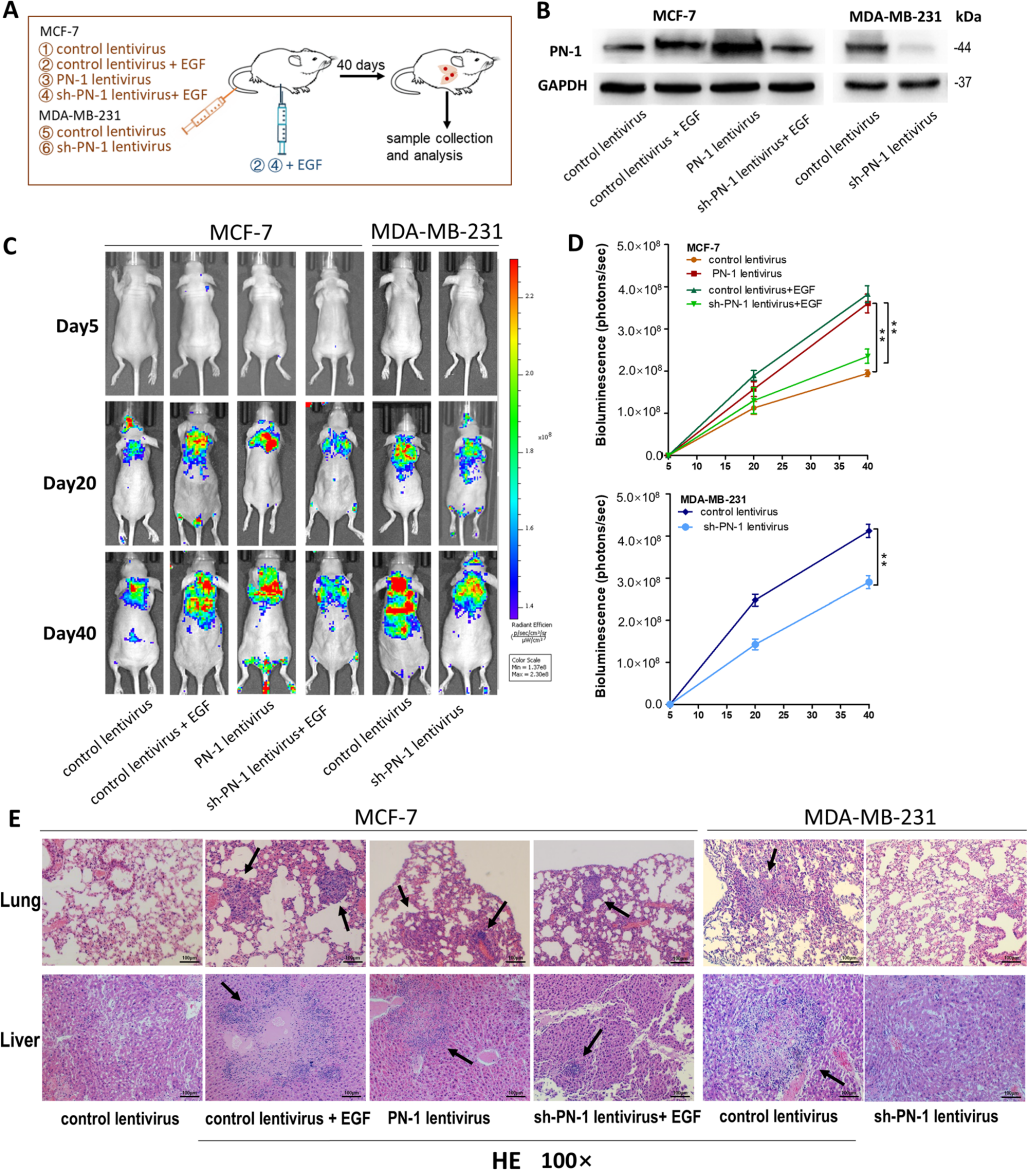
Fig. 7

This has been corrected in the PDF and HTML versions of the article.

The authors apologize for any inconvenience caused.

