# Supplementary material for: Protease Nexin I is a feedback regulator of EGF/PKC/MAPK/EGR1 signaling in breast cancer cells metastasis and stemness
Source: Cell Death Dis. 2019 Sep 9;10(9):649. doi: 10.1038/s41419-019-1882-9 (PMC6733841; doi:10.1038/s41419-019-1882-9)
Supplement: Supplementary file 1 — Supplementary Figure Legends. [file 41419_2019_1882_MOESM1_ESM.docx]

**Figure S1. Effects of PN-1 vector, si-PN-1 and si-EGR1 on mRNA and protein expression in breast cancer cells**

**(A)** PN-1 mRNA(left) and protein(right) levels in MCF-7 cells transfected with control vector or PN-1 vector. **(B)** PN-1 mRNA(left) and protein(right) levels in MCF-7 cells transfected with si-NC or si-PN-1. **(C)** EGR1 mRNA(left) and protein(right) levels in MCF-7 cells transfected with si-NC or si-EGR1. **(D)** EGFR mRNA(left) and protein(right) levels in MCF-7 cells transfected with si-NC or si-EGFR. **(E)** PKCδ mRNA(left) and protein(right) levels in MCF-7 cells transfected with si-NC or si-PKCδ. **(F)** ERK mRNA(left) and protein(right) levels in MCF-7 cells transfected with si-NC or si-ERK. ****P*<0.005.

**Figure S2. PN-1 slightly promotes breast cancer cells proliferation and drug resistance to paclitaxel *in vitro***

**(A)** Proliferation rate of MCF-7 cells transfected with control vector or PN-1 vector detected by CCK8 assay. **(B)** Drug resistance ability of MCF-7 cells transfected with control vector or PN-1 vector. the IC50 values (ng/ml) of chemotherapy drugs paclitaxel, adriamycin and gemcitabine. **P*<0.05.

**Figure S3. EGFR is up-regulated in breast cancer tissues**

**(A)** EGFR mRNA levels were detected in 70 pairs of human breast cancer tissues and corresponding distal non-cancerous tissues by qRT-PCR. **(B)** EGFR and P-EGFR protein levels were detected in 12 pairs of human breast cancer tissues (Cancer) and corresponding distal non-cancerous tissues (Normal) by western blotting. **(C)** P-EGFR protein levels were detected in four breast cancer cell lines. ****P*<0.005.

**Figure S4. PKCδ and ERK1/2 were involved in EGF-induced PN-1 up-regulation.**

**(A)** P-PKCδ and PKCδ protein levels in control MCF-7 cells, MCF-7 cells treated with EGF, MCF-7 cells treated with EGF and AG1478, Go6983, or rottlerin. **(B)** PKCδ protein distribution in control MCF-7 cells, MCF-7 cells treated with PMA (PKC activator), MCF-7 cells treated with EGF, MCF-7 cells treated with EGF and AG1478 (Scale bar: 20μm). **(C, D)** P-ERK1/2 and ERK1/2 protein levels in MCF-7 cells under stimulation of EGF at different doses (C) and for different time durations (D). **(E)** P-ERK1/2 and ERK1/2 protein levels in control MCF-7 cells, MCF-7 cells treated with EGF, and MCF-7 cells treated with EGF and AG1478. **(F)** P-ERK1/2 and ERK1/2 protein levels in control MCF-7 cells, MCF-7 cells treated with EGF, and MCF-7 cells treated with EGF and 10 μM or 20 μM Go6983. ****P*<0.005.

**Figure S5.** **Inhibition of the PKC/MAPK/EGR1 signaling pathway interfere with the ability of PN-1 to modulate invasion, migration and stemness.**

**(A)** P-PKC, P-ERK1/2, EGR1 and PN-1 protein levels in control MCF-7 cells, MCF-7 cells treated with EGF, MCF-7 cells treated with EGF and Go6983, MCF-7 cells treated with EGF and Go6983 and transfected with PN-1 vector. **(B)** Representative images and quantitative analysis of migration and invasion of four different treated MCF-7 cells (scale bar: 100μm). **(C)** The percentage of CD44+/CD24- population in four different treated MCF-7 cells. **(D)** Representative images and quantitative analysis of migration and invasion of four different treated MDA-MB-231 cells (scale bar: 100μm). **(E)** The percentage of CD44+/CD24- population in four different treated MDA-MB-231 cells. ***P*<0.01, ****P*<0.005.
