# Supplementary figures and images for: Protease Nexin I is a feedback regulator of EGF/PKC/MAPK/EGR1 signaling in breast cancer cells metastasis and stemness
Source: Cell Death Dis. 2019 Sep 9;10(9):649. doi: 10.1038/s41419-019-1882-9 (PMC6733841; doi:10.1038/s41419-019-1882-9)

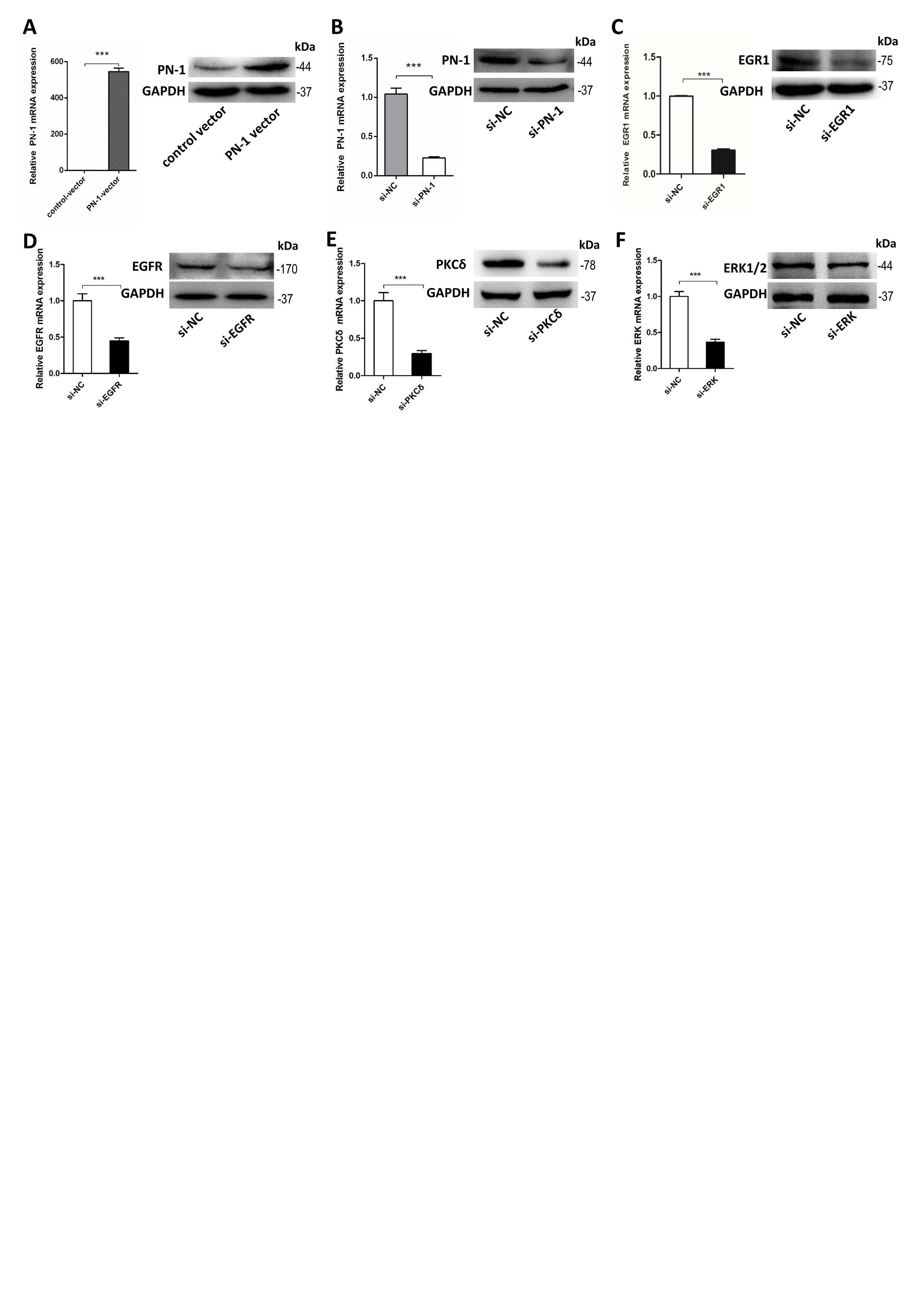

Supplement: Supplementary file 2 — Supplementary Figure S1. [file 41419_2019_1882_MOESM2_ESM.tif]

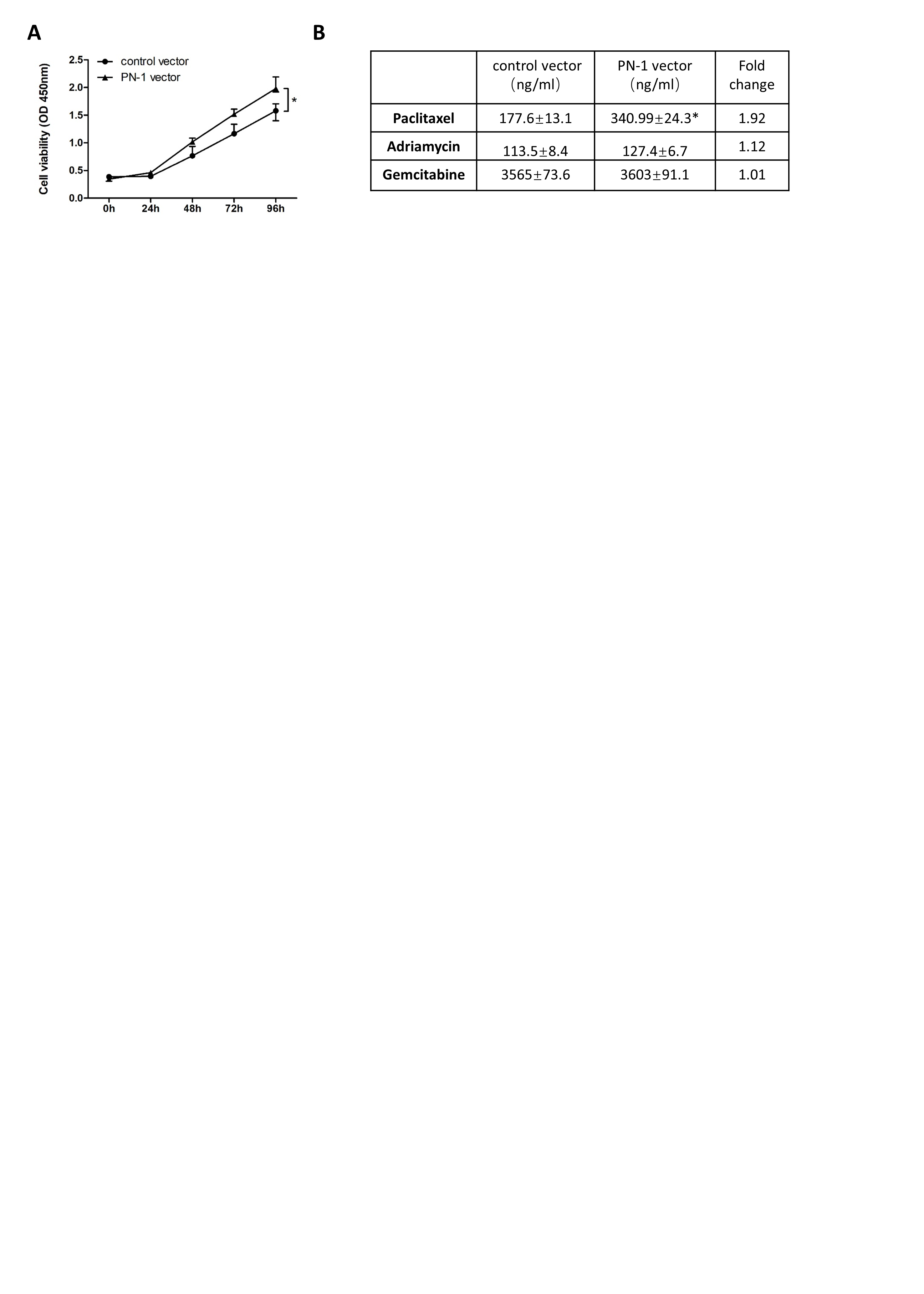

Supplement: Supplementary file 3 — Supplementary Figure S2. [file 41419_2019_1882_MOESM3_ESM.tif]

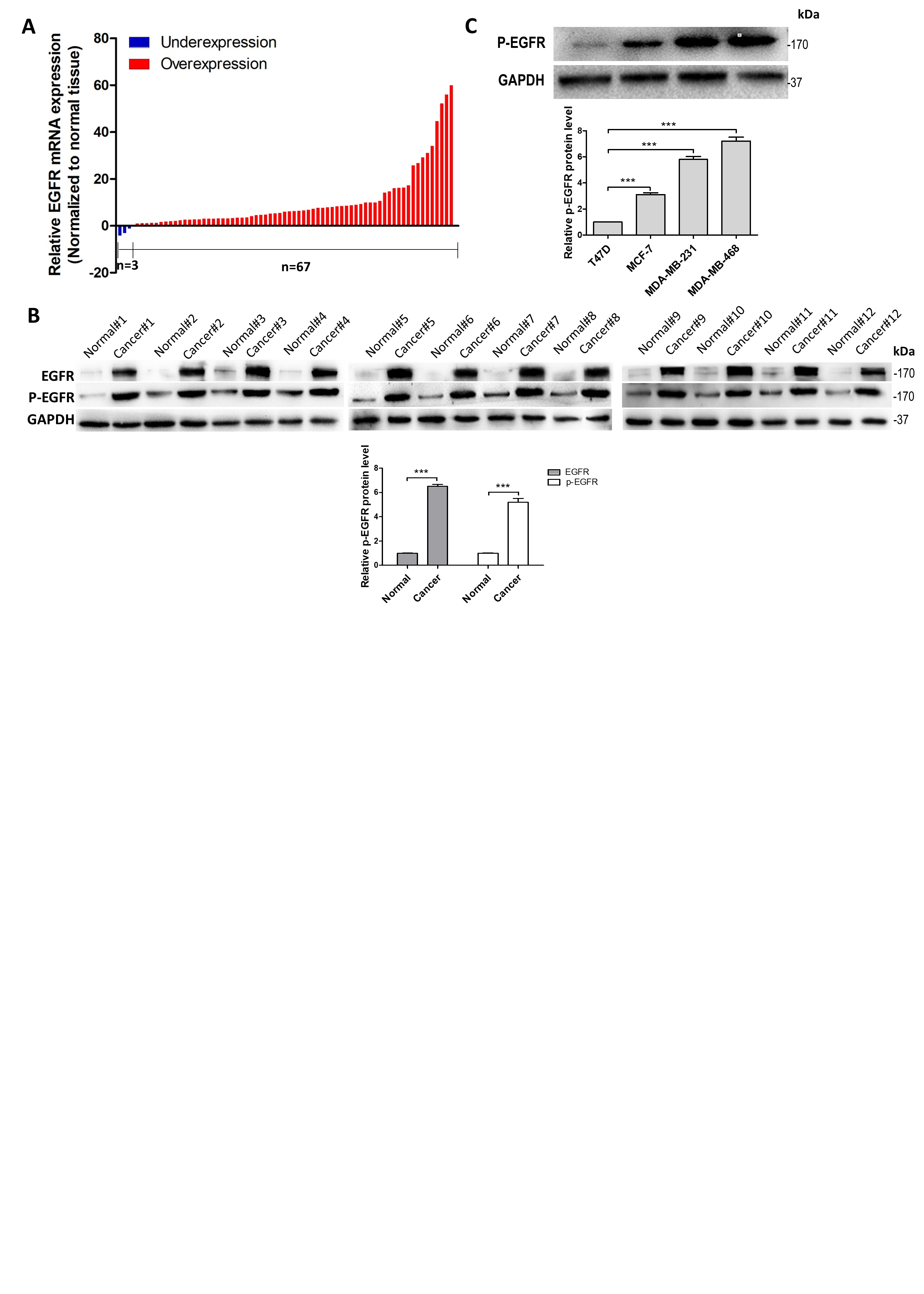

Supplement: Supplementary file 4 — Supplementary Figure S3. [file 41419_2019_1882_MOESM4_ESM.tif]

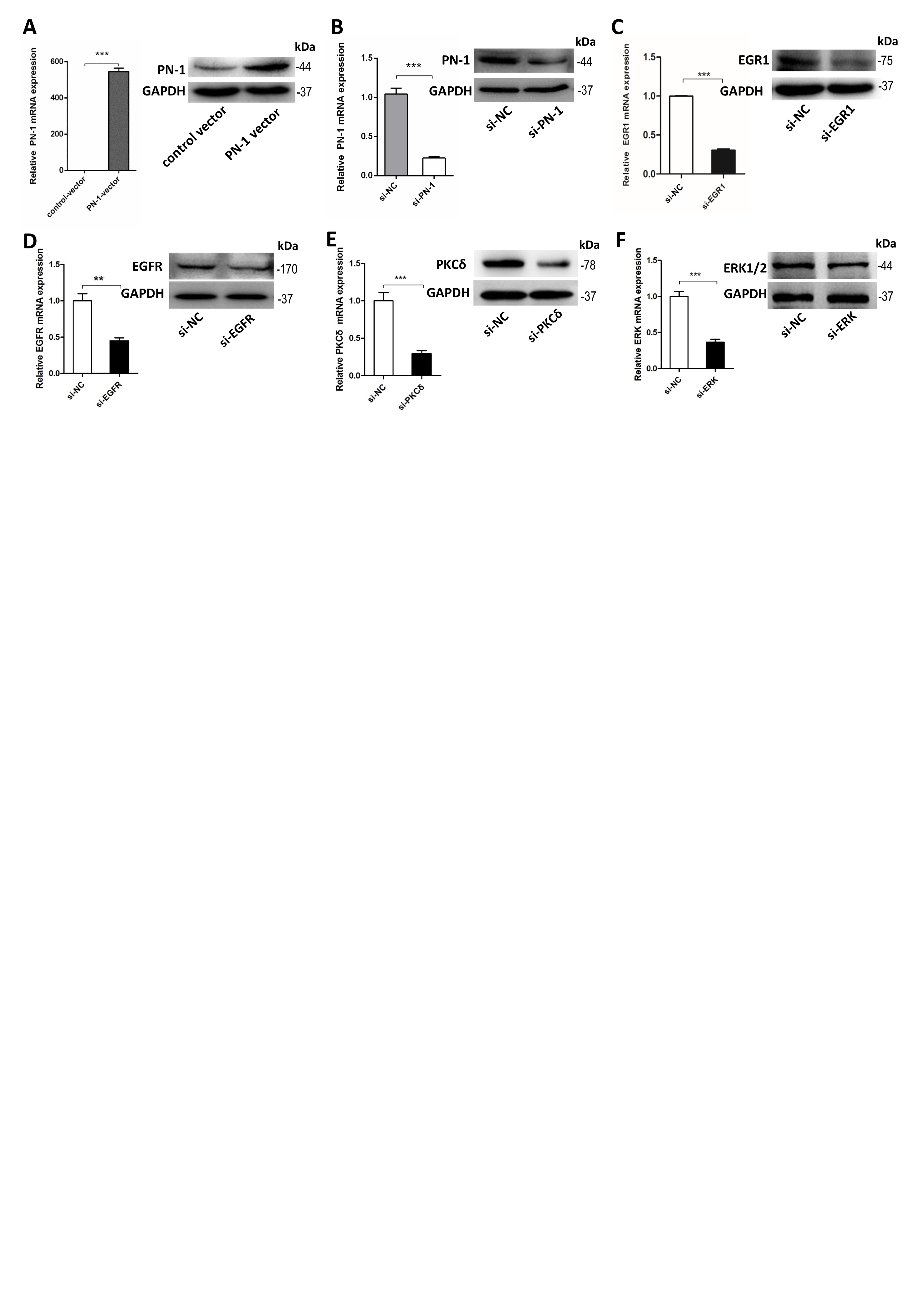

Supplement: Supplementary file 5 — Supplementary Figure S4. [file 41419_2019_1882_MOESM5_ESM.tif]

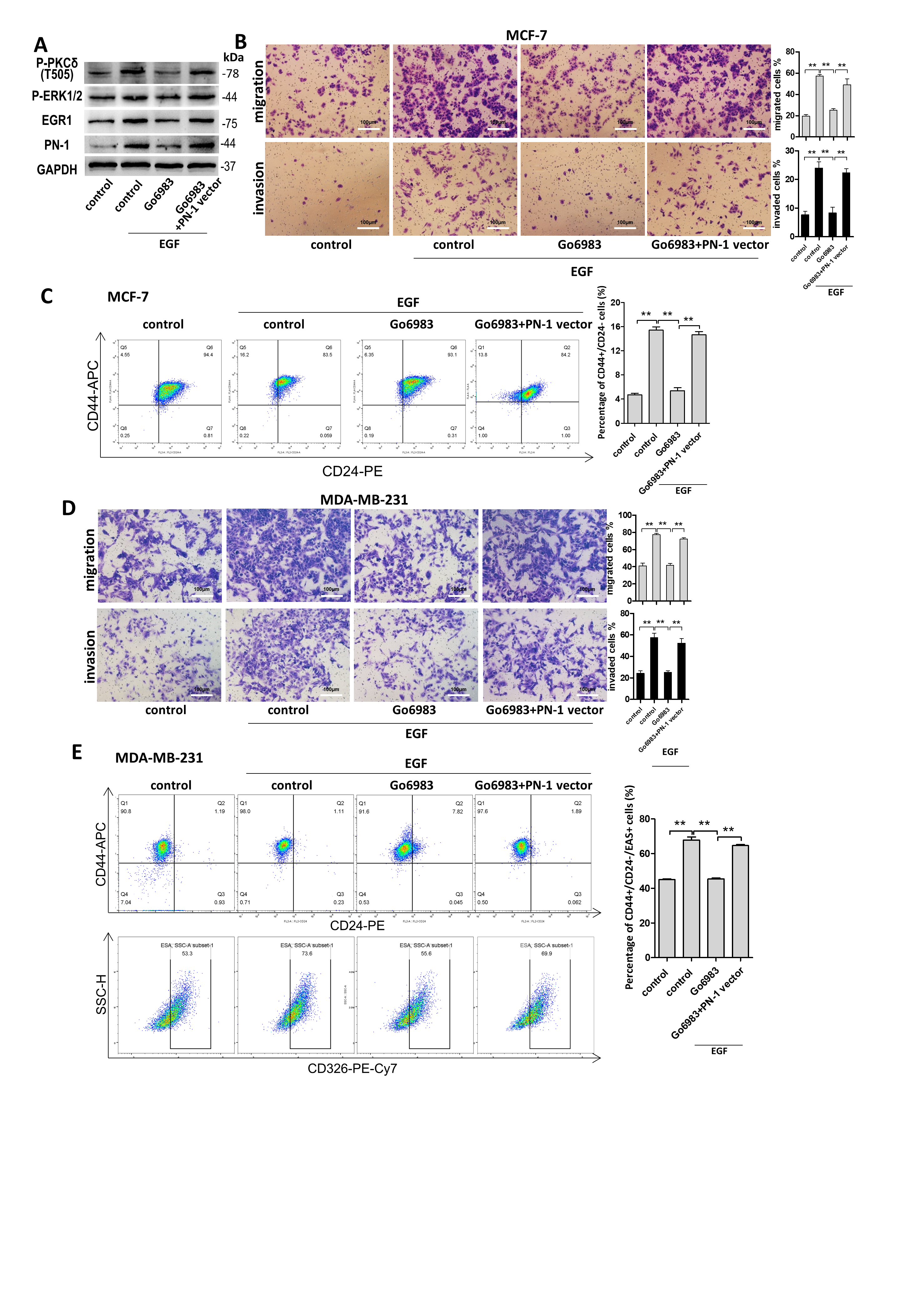

Supplement: Supplementary file 6 — Supplementary Figure S5. [file 41419_2019_1882_MOESM6_ESM.tif]
