# Supplementary material for: Protease Nexin I is a feedback regulator of EGF/PKC/MAPK/EGR1 signaling in breast cancer cells metastasis and stemness
Source: Cell Death Dis. 2019 Sep 9;10(9):649. doi: 10.1038/s41419-019-1882-9 (PMC6733841; doi:10.1038/s41419-019-1882-9)
Supplement: Supplementary file 7 — Supplementary Table S1. [file 41419_2019_1882_MOESM7_ESM.docx]

**Supplementary Table S1. The primers used for qPCR of target genes.**

| Primer name | Sequence（5’ to 3’） |
| --- | --- |
| GAPDH-F | GGAGCGAGATCCCTCCAAAAT |
| GAPDH-R | GGCTGTTGTCATACTTCTCATGG |
| MICB-F | TGGAGACTCAAGAATCGACAGT |
| MICB-R | CTGCATAGCGCGATAGTGTG |
| TRABD-F | GCAAGAGGGACGTTGTGAAGA |
| TRABD-R | GGACACACGATATTGGCAGAG |
| CTAGE5-F | GAGGCCCATTCTTGAGAAGAGG |
| CTAGE5-R | TTGAAGGTGGAATCAAACCTGAG |
| KCNMB2-F | GAGGACCGAGCTATTCTCCTG |
| KCNMB2-R | TGTTTCCGTGATGGACGCATT |
| CD82-F | GCTCATTCGAGACTACAACAGC |
| CD82-R | GTGACCTCAGGGCGATTCA |
| CD109-F | GTAGCATGGCAGTTCATAGTCTG |
| CD109-R | ACCACCAACTCAAAAGGCGAT |
| BRCC3-F | GAGTCTGACGCTTTCCTCGTT |
| BRCC3-R | TGTATCATCGTTCAACTCCCCT |
| HERC3-F | TGTTGGGGATATTGGTCTCTGG |
| HERC3-R | CCCTTGGTGTTCAAACCACAT |
| ARID5A-F | CTGGCAAGCAGAACGGAATC |
| ARID5A-R | GCGTGTGTCGCTCCTTCAT |
| PN-1-F | TGGTGATGAGATACGGCGTAA |
| PN-1-R | GTTAGCCACTGTCACAATGTCTT |
| EGR1-F | GGTCAGTGGCCTAGTGAGC |
| EGR1-R | GTGCCGCTGAGTAAATGGGA |
| EGFR-F | TTGCCGCAAAGTGTGTAACG |
| EGFR-R | GTCACCCCTAAATGCCACCG |
| PKCδ-F | GTCCACAAGAGGTGCCATGAA |
| PKCδ-R | AAGGTGGGGCTTCCGTAAGT |
| ERK-F | TACACCAACCTCTCGTACATCG |
| ERK-R | CATGTCTGAAGCGCAGTAAGATT |
| HTRA1-F | TCCCAACAGTTTGCGCCATAA |
| HTRA1-R | TCCCAACAGTTTGCGCCATAA |
| EGF-F | TGGATGTGCTTGATAAGCGG |
| EGF-R | ACCATGTCCTTTCCAGTGTGT |
