# Supplementary material for: Protease Nexin I is a feedback regulator of EGF/PKC/MAPK/EGR1 signaling in breast cancer cells metastasis and stemness
Source: Cell Death Dis. 2019 Sep 9;10(9):649. doi: 10.1038/s41419-019-1882-9 (PMC6733841; doi:10.1038/s41419-019-1882-9)
Supplement: Supplementary file 8 — Supplementary Table S3. [file 41419_2019_1882_MOESM8_ESM.pdf]

Supplementary Table S2. All the significantly (>2.0-fold; P < 0.05) regulated mRNAs in MCF-7 cells compared to MCF-7 spheroid cells.

gene_id	gene	locus	Database_source	sample_1	sample_2	value_1	value_2	log2 (fold_change)	test_stat	p_value	q_value
ENSG00000001630	CYP51A1	7:92112150-92180725	Protein_coding_from_Ensembl	MCF-7 cells	MCF-7 spheroid cells	33.1042	146.141	2.14227	3.61541	0.0004	0.642964
ENSG00000004799	PDK4	7:95583498-95596491	Protein_coding_from_Ensembl	MCF-7 cells	MCF-7 spheroid cells	12.1666	54.5561	2.16482	3.68768	0.0002	0.432338
ENSG00000006282	SPATA20	17:50532542-50555852	Protein_coding_from_Ensembl	MCF-7 cells	MCF-7 spheroid cells	10.8438	2.57074	-2.07662	-2.25553	0.03915	0.983395
ENSG00000019549	SNAI2	8:48917689-48921740	Protein_coding_from_Ensembl	MCF-7 cells	MCF-7 spheroid cells	20.4769	5.46076	-1.90682	-2.81254	0.00575	0.983395
ENSG00000059804	SLC2A3	12:7919229-7936275	Protein_coding_from_Ensembl	MCF-7 cells	MCF-7 spheroid cells	74.2819	285.773	1.94379	3.00351	0.0034	0.983395
ENSG00000070404	FSTL3	19:676364-683399	Protein_coding_from_Ensembl	MCF-7 cells	MCF-7 spheroid cells	113.227	28.1686	-2.00706	-3.0725	0.00235	0.983395
ENSG00000065308	TRAM2	6:52497401-52576915	Protein_coding_from_Ensembl	MCF-7 cells	MCF-7 spheroid cells	9.18272	3.63977	-1.33507	-2.21022	0.03355	0.983395
ENSG00000068912	ERLEC1	2:53532671-53860160	Protein_coding_from_Ensembl	MCF-7 cells	MCF-7 spheroid cells	15.9084	52.0622	1.71045	2.09209	0.04525	0.983395
ENSG00000092929	UNC13D	17:75827224-75844717	Protein_coding_from_Ensembl	MCF-7 cells	MCF-7 spheroid cells	3.22089	0.619231	-2.37891	-2.22446	0.0458	0.983395
ENSG00000100078	PLA2G3	22:31134808-31140607	Protein_coding_from_Ensembl	MCF-7 cells	MCF-7 spheroid cells	0.156171	1.93387	3.6303	3.08609	0.0232	0.983395
ENSG00000100292	HMOX1	22:35380360-35394214	Protein_coding_from_Ensembl	MCF-7 cells	MCF-7 spheroid cells	2.50023	8.4431	1.75571	2.12593	0.04195	0.983395
ENSG00000100883	SRP54	14:34920857-35029567	Protein_coding_from_Ensembl	MCF-7 cells	MCF-7 spheroid cells	25.1946	58.879	1.22464	2.00636	0.04675	0.983395
ENSG00000100889	PCK2	14:24080106-24132849	Protein_coding_from_Ensembl	MCF-7 cells	MCF-7 spheroid cells	28.5165	5.87476	-2.2792	-2.25602	0.0306	0.983395
ENSG00000100906	NFKBIA	14:35401510-35404749	Protein_coding_from_Ensembl	MCF-7 cells	MCF-7 spheroid cells	29.5862	108.63	1.87642	2.78612	0.00675	0.983395
ENSG00000101057	MYBL2	20:43667018-43716496	Protein_coding_from_Ensembl	MCF-7 cells	MCF-7 spheroid cells	43.9676	13.3955	-1.71469	-2.87423	0.0042	0.983395
ENSG00000101160	CTSZ	20:58995184-59007247	Protein_coding_from_Ensembl	MCF-7 cells	MCF-7 spheroid cells	412.297	88.9894	-2.21198	-3.84001	0.00025	0.505556
ENSG00000101187	SLCO4A1	20:62640718-62685785	Protein_coding_from_Ensembl	MCF-7 cells	MCF-7 spheroid cells	15.9578	69.4082	2.12084	3.15521	0.00085	0.983395
ENSG00000101311	FERMT1	20:6074844-6123544	Protein_coding_from_Ensembl	MCF-7 cells	MCF-7 spheroid cells	6.14164	2.21793	-1.46941	-2.16891	0.02735	0.983395
ENSG00000102265	TIMP1	X:47561099-47619853	Protein_coding_from_Ensembl	MCF-7 cells	MCF-7 spheroid cells	178.667	835.265	2.22496	3.07096	0.0039	0.983395
ENSG00000103175	WFDC1	16:84294645-84329851	Protein_coding_from_Ensembl	MCF-7 cells	MCF-7 spheroid cells	1.35302	23.5227	4.1198	4.27705	0.0032	0.983395
ENSG00000079459	FDFT1	8:11795572-11839309	Protein_coding_from_Ensembl	MCF-7 cells	MCF-7 spheroid cells	8.55251	58.5438	2.7751	2.7002	0.0136	0.983395
ENSG00000076641	PAG1	8:80967809-81112068	Protein_coding_from_Ensembl	MCF-7 cells	MCF-7 spheroid cells	0.489085	3.27593	2.74375	3.64455	0.00045	0.671668
ENSG00000104368	PLAT	8:42175232-42207724	Protein_coding_from_Ensembl	MCF-7 cells	MCF-7 spheroid cells	1.23683	11.7678	3.25013	3.80505	0.0038	0.983395
ENSG00000128510	CPA4	7:130293133-130324180	Protein_coding_from_Ensembl	MCF-7 cells	MCF-7 spheroid cells	9.1403	0.730941	-3.64441	-3.6729	0.00665	0.983395
ENSG00000105835	NAMPT	7:106248284-106286326	Protein_coding_from_Ensembl	MCF-7 cells	MCF-7 spheroid cells	54.0318	160.581	1.57142	2.02114	0.0439	0.983395
ENSG00000105855	ITGB8	7:20328298-20415754	Protein_coding_from_Ensembl	MCF-7 cells	MCF-7 spheroid cells	5.75979	20.1901	1.80956	2.99032	0.003	0.983395
ENSG00000105971	CAV2	7:116209233-116508541	Protein_coding_from_Ensembl	MCF-7 cells	MCF-7 spheroid cells	37.6157	9.21379	-2.02947	-2.18342	0.03435	0.983395
ENSG00000106025	TSPAN12	7:120787319-120858402	Protein_coding_from_Ensembl	MCF-7 cells	MCF-7 spheroid cells	3.18819	10.1755	1.67429	2.12663	0.04285	0.983395
ENSG00000106080	FKBP14	7:29920102-30026684	Protein_coding_from_Ensembl	MCF-7 cells	MCF-7 spheroid cells	5.04642	12.7665	1.33903	2.10613	0.03455	0.983395
ENSG00000109072	VTN	17:28357580-28407197	Protein_coding_from_Ensembl	MCF-7 cells	MCF-7 spheroid cells	30.3274	6.17732	-2.29557	-1.87782	0.04775	0.983395
ENSG00000085117	CD82	11:44564426-44620363	Protein_coding_from_Ensembl	MCF-7 cells	MCF-7 spheroid cells	4.7519	0.701808	-2.75936	-2.72132	0.01925	0.983395
ENSG00000110042	DTX4	11:59171429-59212951	Protein_coding_from_Ensembl	MCF-7 cells	MCF-7 spheroid cells	1.83784	0.125946	-3.86714	-3.15327	0.03135	0.983395
ENSG00000111711	GOLT1B	12:21501780-21518408	Protein_coding_from_Ensembl	MCF-7 cells	MCF-7 spheroid cells	16.0442	51.5065	1.6827	2.63966	0.00855	0.983395
ENSG00000112414	ADGRG6	6:142301853-142446266	Protein_coding_from_Ensembl	MCF-7 cells	MCF-7 spheroid cells	23.604	8.75704	-1.43052	-2.29278	0.0238	0.983395
ENSG00000114315	HES1	3:194136144-194138732	Protein_coding_from_Ensembl	MCF-7 cells	MCF-7 spheroid cells	7.11913	21.0531	1.56426	2.20026	0.0316	0.983395
ENSG00000021826	CPS1	2:210477681-210679107	Protein_coding_from_Ensembl	MCF-7 cells	MCF-7 spheroid cells	435.278	122.27	-1.83187	-2.58564	0.0128	0.983395
ENSG00000115540	MOB4	2:197486580-197553699	Protein_coding_from_Ensembl	MCF-7 cells	MCF-7 spheroid cells	0	1.10499	1.07381338	NA	0.03995	0.983395
ENSG00000115596	WNT6	2:218859820-218874233	Protein_coding_from_Ensembl	MCF-7 cells	MCF-7 spheroid cells	2.0647	0.209773	-3.29903	-2.70413	0.0421	0.983395
ENSG00000116741	RGS2	1:192809038-192812283	Protein_coding_from_Ensembl	MCF-7 cells	MCF-7 spheroid cells	18.2365	67.3304	1.88443	2.99382	0.00285	0.983395
ENSG00000119938	PPP1R3C	10:91628441-91633054	Protein_coding_from_Ensembl	MCF-7 cells	MCF-7 spheroid cells	33.7908	4.65953	-2.85837	-4.21191	0.0003	0.537334
ENSG00000120137	PANK3	5:168548494-168579600	Protein_coding_from_Ensembl	MCF-7 cells	MCF-7 spheroid cells	15.532	39.6928	1.35363	2.4003	0.0152	0.983395
ENSG00000120738	EGR1	5:138465489-138469315	Protein_coding_from_Ensembl	MCF-7 cells	MCF-7 spheroid cells	6.71087	1.95168	-1.78178	-2.35829	0.0191	0.983395
ENSG00000120875	DUSP4	8:29333063-29350668	Protein_coding_from_Ensembl	MCF-7 cells	MCF-7 spheroid cells	4.19413	17.0063	2.01962	3.25006	0.00115	0.983395
ENSG00000121966	CXCR4	2:136114348-136118165	Protein_coding_from_Ensembl	MCF-7 cells	MCF-7 spheroid cells	25.6374	68.9023	1.4263	2.39272	0.01785	0.983395
ENSG00000122862	SRGN	10:69088105-69104811	Protein_coding_from_Ensembl	MCF-7 cells	MCF-7 spheroid cells	47.7739	267.031	2.48271	4.24902	5.00E-05	0.174136
ENSG00000123610	TNFAIP6	2:151357591-151380048	Protein_coding_from_Ensembl	MCF-7 cells	MCF-7 spheroid cells	0	1.45054	1.293099696	NA	0.00015	0.335834
ENSG00000124466	LYPD3	19:43460786-43465660	Protein_coding_from_Ensembl	MCF-7 cells	MCF-7 spheroid cells	9.00668	32.4823	1.85059	2.76517	0.00915	0.983395
ENSG00000124762	CDKN1A	6:36676459-36687339	Protein_coding_from_Ensembl	MCF-7 cells	MCF-7 spheroid cells	11.852	84.4779	2.83344	4.39389	0.0001	0.241112
ENSG00000125148	MT2A	16:56608198-56609497	Protein_coding_from_Ensembl	MCF-7 cells	MCF-7 spheroid cells	27.4088	299.857	3.45156	5.14323	5.00E-05	0.174136
ENSG00000125657	TNFSF9	19:6530998-6535928	Protein_coding_from_Ensembl	MCF-7 cells	MCF-7 spheroid cells	17.5579	74.7005	2.089	3.48978	0.0013	0.983395
ENSG00000125730	C3	19:6677703-6737603	Protein_coding_from_Ensembl	MCF-7 cells	MCF-7 spheroid cells	29.006	6.75489	-2.10235	-2.54877	0.0142	0.983395
ENSG00000127527	EPS15L1	19:16355238-16472085	Protein_coding_from_Ensembl	MCF-7 cells	MCF-7 spheroid cells	0.000125	3.08485	14.5912	0.063693	0.0315	0.983395
ENSG00000128283	CDC42EP1	22:37560446-37569405	Protein_coding_from_Ensembl	MCF-7 cells	MCF-7 spheroid cells	51.5654	22.9458	-1.16817	-2.01928	0.04485	0.983395
ENSG00000128564	VGF	7:101162508-101165593	Protein_coding_from_Ensembl	MCF-7 cells	MCF-7 spheroid cells	0.410025	4.1608	3.34308	3.41805	0.0059	0.983395
ENSG00000100867	DHRS2	14:23630114-23645639	Protein_coding_from_Ensembl	MCF-7 cells	MCF-7 spheroid cells	0.633981	3.68663	2.53979	2.32235	0.04105	0.983395
ENSG00000112379	ARFGEF3	6:138161920-138344663	Protein_coding_from_Ensembl	MCF-7 cells	MCF-7 spheroid cells	0.347709	1.85564	2.41597	3.19843	0.0039	0.983395
ENSG00000204516	MICB	6:31494880-31511124	Protein_coding_from_Ensembl	MCF-7 cells	MCF-7 spheroid cells	0.00024	19.7303	16.3298	0.112822	0.04615	0.983395
ENSG00000130477	UNC13A	19:17601327-17688365	Protein_coding_from_Ensembl	MCF-7 cells	MCF-7 spheroid cells	0.271654	5.31246	4.28954	3.9721	0.0053	0.983395
ENSG00000130508	PXDN	2:1631886-1744852	Protein_coding_from_Ensembl	MCF-7 cells	MCF-7 spheroid cells	9.17448	3.70572	-1.30787	-2.10405	0.0354	0.983395
ENSG00000130513	GDF15	19:18374730-18389176	Protein_coding_from_Ensembl	MCF-7 cells	MCF-7 spheroid cells	35.8673	627.758	4.12947	6.88191	5.00E-05	0.174136
ENSG00000130638	ATXN10	22:45657018-45845307	Protein_coding_from_Ensembl	MCF-7 cells	MCF-7 spheroid cells	17.6428	4.52503	-1.96308	-2.12926	0.0393	0.983395
ENSG00000131408	NR1H2	19:50311936-50382982	Protein_coding_from_Ensembl	MCF-7 cells	MCF-7 spheroid cells	9.11931	23.628	1.3735	1.97893	0.04845	0.983395
ENSG00000131459	GFPT2	5:180300689-180353387	Protein_coding_from_Ensembl	MCF-7 cells	MCF-7 spheroid cells	16.4289	44.3362	1.43225	2.37687	0.0174	0.983395
ENSG00000131791	PRKAB2	1:147155105-147172550	Protein_coding_from_Ensembl	MCF-7 cells	MCF-7 spheroid cells	8.43767	3.38868	-1.31612	-2.06485	0.04225	0.983395
ENSG00000132471	WBP2	17:75845698-75856507	Protein_coding_from_Ensembl	MCF-7 cells	MCF-7 spheroid cells	12.1454	35.8863	1.56302	2.28351	0.02335	0.983395
ENSG00000134070	IRAK2	3:10164864-10243743	Protein_coding_from_Ensembl	MCF-7 cells	MCF-7 spheroid cells	1.01158	7.35961	2.86303	3.59446	0.00235	0.983395
ENSG00000134107	BHLHE40	3:4896808-4985323	Protein_coding_from_Ensembl	MCF-7 cells	MCF-7 spheroid cells	25.2718	65.3868	1.37147	2.40418	0.01815	0.983395
ENSG00000134243	SORT1	1:109309567-109397951	Protein_coding_from_Ensembl	MCF-7 cells	MCF-7 spheroid cells	6.17325	1.63131	-1.92	-2.5261	0.01845	0.983395
ENSG00000134363	FST	5:53480408-53487134	Protein_coding_from_Ensembl	MCF-7 cells	MCF-7 spheroid cells	0.689209	4.03921	2.55106	2.40662	0.04285	0.983395
ENSG00000134508	CABLES1	18:23134563-23437961	Protein_coding_from_Ensembl	MCF-7 cells	MCF-7 spheroid cells	4.63605	1.07582	-2.10746	-2.09551	0.0495	0.983395
ENSG00000134825	TMEM258	11:61680432-61867354	Protein_coding_from_Ensembl	MCF-7 cells	MCF-7 spheroid cells	0	4.64073	2.495881882	NA	0.0414	0.983395
ENSG00000135318	NT5E	6:85449583-85495791	Protein_coding_from_Ensembl	MCF-7 cells	MCF-7 spheroid cells	3.89531	41.886	3.42666	4.95622	5.00E-05	0.174136
ENSG00000135919	SERPINE2	2:223975111-224039319	Protein_coding_from_Ensembl	MCF-7 cells	MCF-7 spheroid cells	4.56E-05	6.32269	17.0822	0.019965	0.03005	0.983395
ENSG00000136379	ABHD17C	15:80679683-80755621	Protein_coding_from_Ensembl	MCF-7 cells	MCF-7 spheroid cells	12.7151	44.7079	1.81398	2.86155	0.00445	0.983395
ENSG00000137177	KIF13A	6:17759182-17987623	Protein_coding_from_Ensembl	MCF-7 cells	MCF-7 spheroid cells	26.7653	7.22023	-1.89025	-2.13471	0.03825	0.983395
ENSG00000137331	IER3	6:30742928-30744554	Protein_coding_from_Ensembl	MCF-7 cells	MCF-7 spheroid cells	40.6022	601.679	3.88937	5.90781	5.00E-05	0.174136
ENSG00000052802	MSMO1	4:165327622-165343160	Protein_coding_from_Ensembl	MCF-7 cells	MCF-7 spheroid cells	3.42807	68.1762	4.3138	5.39033	5.00E-05	0.174136
ENSG00000103257	SLC7A5	16:87830022-87869488	Protein_coding_from_Ensembl	MCF-7 cells	MCF-7 spheroid cells	596.026	175.406	-1.76468	-2.34414	0.02195	0.983395
ENSG00000128833	MYO5C	15:52115104-52295798	Protein_coding_from_Ensembl	MCF-7 cells	MCF-7 spheroid cells	18.6844	3.19234	-2.54914	-3.0267	0.0087	0.983395
ENSG00000138613	APH1B	15:63276017-63309126	Protein_coding_from_Ensembl	MCF-7 cells	MCF-7 spheroid cells	2.28876	0.519139	-2.14037	-2.30933	0.03575	0.983395
ENSG00000041353	RAB27B	18:54717859-54898083	Protein_coding_from_Ensembl	MCF-7 cells	MCF-7 spheroid cells	8.39928	18.615	1.14813	1.99251	0.04165	0.983395
ENSG00000186951	PPARA	22:46150520-46243756	Protein_coding_from_Ensembl	MCF-7 cells	MCF-7 spheroid cells	0.619427	3.45033	2.47773	2.25245	0.0486	0.983395
ENSG00000109991	P2RX3	11:57338373-57370600	Protein_coding_from_Ensembl	MCF-7 cells	MCF-7 spheroid cells	2.06336	0.303891	-2.76337	-2.37727	0.03975	0.983395
ENSG00000023445	BIRC3	11:102317449-10233940	Protein_coding_from_Ensembl	MCF-7 cells	MCF-7 spheroid cells	10.1196	28.3512	1.48626	2.56193	0.01185	0.983395
ENSG00000094975	SUCO	1:172532348-172611833	Protein_coding_from_Ensembl	MCF-7 cells	MCF-7 spheroid cells	4.36795	12.1344	1.47408	2.14803	0.02795	0.983395
ENSG00000114166	KAT2B	3:20040022-20154404	Protein_coding_from_Ensembl	MCF-7 cells	MCF-7 spheroid cells	4.63237	1.33761	-1.79209	-2.37234	0.0195	0.983395
ENSG00000115963	RND3	2:150468194-150539011	Protein_coding_from_Ensembl	MCF-7 cells	MCF-7 spheroid cells	17.5613	43.0931	1.29506	2.02066	0.04215	0.983395
ENSG00000115520	COQ10B	2:197453422-197475308	Protein_coding_from_Ensembl	MCF-7 cells	MCF-7 spheroid cells	5.23598	15.6144	1.57635	2.18579	0.03065	0.983395
ENSG00000079257	LXN	3:158644277-158692575	Protein_coding_from_Ensembl	MCF-7 cells	MCF-7 spheroid cells	164.296	21.4619	-2.93645	-3.11544	0.0069	0.983395
ENSG00000138641	HERC3	4:88520984-88708542	Protein_coding_from_Ensembl	MCF-7 cells	MCF-7 spheroid cells	1.30217	7.29E-05	-14.1245	-0.05992	0.03695	0.983395
ENSG00000138650	PCDH10	4:133149314-133208606	Protein_coding_from_Ensembl	MCF-7 cells	MCF-7 spheroid cells	0.600811	0.043647	-3.78296	-3.17958	0.02465	0.983395
ENSG00000114200	BCHE	3:165772903-165837472	Protein_coding_from_Ensembl	MCF-7 cells	MCF-7 spheroid cells	4.97538	0.747821	-2.73404	-2.7194	0.01985	0.983395
ENSG00000040731	CDH10	5:24487099-24644978	Protein_coding_from_Ensembl	MCF-7 cells	MCF-7 spheroid cells	61.3928	6.8296	-3.1682	-4.59972	5.00E-05	0.174136
ENSG00000090339	ICAM1	19:10251900-10289019	Protein_coding_from_Ensembl	MCF-7 cells	MCF-7 spheroid cells	22.7747	66.5872	1.54781	2.32299	0.02185	0.983395
ENSG00000109586	GALNT7	4:173131927-173329694	Protein_coding_from_Ensembl	MCF-7 cells	MCF-7 spheroid cells	26.6908	4.6739	-2.51364	-3.73765	0.0004	0.642964
ENSG00000074416	MGLL	3:127689061-127823250	Protein_coding_from_Ensembl	MCF-7 cells	MCF-7 spheroid cells	1.4921	0.000112	-13.6952	-0.08725	0.04965	0.983395
ENSG00000114859	CLCN2	3:184134018-184684758	Protein_coding_from_Ensembl	MCF-7 cells	MCF-7 spheroid cells	1.87913	0	-1.52563293	NA	0.03935	0.983395
ENSG00000105810	CDK6	7:92604920-92917187	Protein_coding_from_Ensembl	MCF-7 cells	MCF-7 spheroid cells	25.4126	9.73961	-1.38361	-2.41313	0.0154	0.983395
ENSG00000104549	SQLE	8:124998496-125022283	Protein_coding_from_Ensembl	MCF-7 cells	MCF-7 spheroid cells	12.5047	96.4243	2.94693	4.88513	5.00E-05	0.174136
ENSG00000100234	TIMP3	22:32512551-33058372	Protein_coding_from_Ensembl	MCF-7 cells	MCF-7 spheroid cells	18.8413	5.78832	-1.70268	-2.83121	0.0052	0.983395
ENSG00000139832	RAB20	13:110523065-11056173	Protein_coding_from_Ensembl	MCF-7 cells	MCF-7 spheroid cells	36.0357	15.7775	-1.19156	-1.93068	0.0498	0.983395

ENSG00000140044	JDP2	14:75423682-75474111	Protein_coding_from_Ensembl	MCF-7 cells	MCF-7 spheroid cells	23.4246	6.82127	-1.77991	-2.21648	0.03465	0.983395
ENSG00000141384	TAF4B	18:26225935-26391685	Protein_coding_from_Ensembl	MCF-7 cells	MCF-7 spheroid cells	1.80386	9.34E-05	-14.2369	-0.08579	0.0254	0.983395
ENSG00000141458	NPC1	18:23503469-23586898	Protein_coding_from_Ensembl	MCF-7 cells	MCF-7 spheroid cells	15.6922	109.331	2.80059	4.022	0.0001	0.241112
ENSG00000142552	RCN3	19:49527617-49546962	Protein_coding_from_Ensembl	MCF-7 cells	MCF-7 spheroid cells	25.5965	79.4605	1.63429	2.53013	0.01355	0.983395
ENSG00000163191	S100A11	1:151994530-152047907	Protein_coding_from_Ensembl	MCF-7 cells	MCF-7 spheroid cells	492.956	1168.58	1.24522	2.13084	0.0345	0.983395
ENSG00000143816	WNT9A	1:227918655-227947898	Protein_coding_from_Ensembl	MCF-7 cells	MCF-7 spheroid cells	2.16021	0.31147	-2.794	-2.92224	0.00975	0.983395
ENSG00000144136	SLC20A1	2:112645856-112663827	Protein_coding_from_Ensembl	MCF-7 cells	MCF-7 spheroid cells	29.1199	69.4368	1.2537	2.05861	0.0369	0.983395
ENSG00000145569	FAM105A	5:14581774-14615007	Protein_coding_from_Ensembl	MCF-7 cells	MCF-7 spheroid cells	22.0033	3.69619	-2.57361	-3.9003	0.00045	0.671668
ENSG00000147155	EBP	X:48521157-48528716	Protein_coding_from_Ensembl	MCF-7 cells	MCF-7 spheroid cells	90.432	216.37	1.2586	2.07396	0.03245	0.983395
ENSG00000147872	PLIN2	9:19108374-19149290	Protein_coding_from_Ensembl	MCF-7 cells	MCF-7 spheroid cells	15.5611	55.1218	1.82468	3.02154	0.00175	0.983395
ENSG00000221968	FADS3	11:61873518-61892051	Protein_coding_from_Ensembl	MCF-7 cells	MCF-7 spheroid cells	31.2511	9.37655	-1.73678	-2.08971	0.0462	0.983395
ENSG00000149573	MPZL2	11:118253402-11826453	Protein_coding_from_Ensembl	MCF-7 cells	MCF-7 spheroid cells	2.4127	14.228	2.56002	3.18812	0.00505	0.983395
ENSG00000152270	PDE3B	11:14643722-14872044	Protein_coding_from_Ensembl	MCF-7 cells	MCF-7 spheroid cells	7.24549	2.76731	-1.3886	-1.99821	0.04005	0.983395
ENSG00000213949	ITGA1	5:52675192-53094779	Protein_coding_from_Ensembl	MCF-7 cells	MCF-7 spheroid cells	17.7516	83.006	2.22527	2.20767	0.04785	0.983395
ENSG00000057149	SERPINB3	18:63637258-63726432	Protein_coding_from_Ensembl	MCF-7 cells	MCF-7 spheroid cells	3.81344	48.0226	3.65455	4.60769	0.0001	0.241112
ENSG00000154127	UBASH3B	11:122655674-12281447	Protein_coding_from_Ensembl	MCF-7 cells	MCF-7 spheroid cells	4.02014	10.5294	1.38911	2.24304	0.02305	0.983395
ENSG00000085662	AKR1B1	7:134442349-134459284	Protein_coding_from_Ensembl	MCF-7 cells	MCF-7 spheroid cells	40.976	375.258	3.19503	5.22108	5.00E-05	0.174136
ENSG00000156535	CD109	6:73693902-73828316	Protein_coding_from_Ensembl	MCF-7 cells	MCF-7 spheroid cells	17.732	7.7189	-1.19989	-1.99257	0.0447	0.983395
ENSG00000113161	HMGCR	5:75320154-75362104	Protein_coding_from_Ensembl	MCF-7 cells	MCF-7 spheroid cells	6.94957	26.7197	1.94291	2.9107	0.0062	0.983395
ENSG00000158050	DUSP2	2:96143165-96145440	Protein_coding_from_Ensembl	MCF-7 cells	MCF-7 spheroid cells	18.4152	3.51022	-2.39127	-3.02612	0.00855	0.983395
ENSG00000158104	HPD	12:121839526-12186359	Protein_coding_from_Ensembl	MCF-7 cells	MCF-7 spheroid cells	62.8411	5.97893	-3.39375	-4.84438	0.0001	0.241112
ENSG00000159167	STC1	8:23841914-23854807	Protein_coding_from_Ensembl	MCF-7 cells	MCF-7 spheroid cells	0.19461	43.2486	7.79592	7.614	0.004	0.983395
ENSG00000162878	PKDCC	2:42048019-42058528	Protein_coding_from_Ensembl	MCF-7 cells	MCF-7 spheroid cells	13.3141	1.61855	-3.04018	-2.99248	0.0116	0.983395
ENSG00000163520	FBLN2	3:13549130-13638422	Protein_coding_from_Ensembl	MCF-7 cells	MCF-7 spheroid cells	15.6133	1.64649	-3.24531	-4.41348	0.0003	0.537334
ENSG00000163993	S100P	4:6693068-6697170	Protein_coding_from_Ensembl	MCF-7 cells	MCF-7 spheroid cells	103.156	43.3323	-1.25131	-1.94425	0.04775	0.983395
ENSG00000164171	ITGA2	5:52675192-53094779	Protein_coding_from_Ensembl	MCF-7 cells	MCF-7 spheroid cells	11.8268	86.6602	2.87331	2.16657	0.04175	0.983395
ENSG00000164188	RANBP3L	5:36248433-36302114	Protein_coding_from_Ensembl	MCF-7 cells	MCF-7 spheroid cells	0.156839	3.41873	4.4461	3.91393	0.01245	0.983395
ENSG00000164211	STARD4	5:111496032-112017309	Protein_coding_from_Ensembl	MCF-7 cells	MCF-7 spheroid cells	4.04055	15.2915	1.92011	2.24054	0.0367	0.983395
ENSG00000146072	TNFRSF21	6:47231531-47309905	Protein_coding_from_Ensembl	MCF-7 cells	MCF-7 spheroid cells	3.68339	15.9775	2.11693	3.25283	0.0025	0.983395
ENSG00000164976	KIAA1161	9:34366669-34376853	Protein_coding_from_Ensembl	MCF-7 cells	MCF-7 spheroid cells	2.09675	0.284477	-2.88177	-3.16194	0.01035	0.983395
ENSG00000165507	C10orf10	10:44811023-44995891	Protein_coding_from_Ensembl	MCF-7 cells	MCF-7 spheroid cells	0.60785	4.94864	3.02524	2.56523	0.0401	0.983395
ENSG00000166106	ADAMTS1	11:130448973-13047664	Protein_coding_from_Ensembl	MCF-7 cells	MCF-7 spheroid cells	1.39547	0.247777	-2.49363	-2.59423	0.0159	0.983395
ENSG00000111670	GNPTAB	12:101745496-10183093	Protein_coding_from_Ensembl	MCF-7 cells	MCF-7 spheroid cells	11.2505	26.8383	1.25431	2.15128	0.0301	0.983395
ENSG00000167107	ACSF2	17:50426157-50474845	Protein_coding_from_Ensembl	MCF-7 cells	MCF-7 spheroid cells	11.7705	3.78269	-1.63769	-2.12383	0.0336	0.983395
ENSG00000103335	PIEZO1	16:88706462-88785211	Protein_coding_from_Ensembl	MCF-7 cells	MCF-7 spheroid cells	26.854	7.37059	-1.86528	-2.84193	0.0059	0.983395
ENSG00000186832	KRT16	17:41609777-41615899	Protein_coding_from_Ensembl	MCF-7 cells	MCF-7 spheroid cells	1.18573	14.5531	3.61748	3.38072	0.0143	0.983395
ENSG00000168077	SCARA3	8:27633867-27676776	Protein_coding_from_Ensembl	MCF-7 cells	MCF-7 spheroid cells	11.3878	1.21371	-3.22999	-4.07205	0.0006	0.85485
ENSG00000169174	PCSK9	1:55039547-55064852	Protein_coding_from_Ensembl	MCF-7 cells	MCF-7 spheroid cells	12.4761	38.2581	1.61659	2.76251	0.00655	0.983395
ENSG00000169439	SDC2	8:96493350-96611780	Protein_coding_from_Ensembl	MCF-7 cells	MCF-7 spheroid cells	11.5751	2.67694	-2.11237	-2.546	0.01755	0.983395
ENSG00000171444	MCC	5:113022098-113488830	Protein_coding_from_Ensembl	MCF-7 cells	MCF-7 spheroid cells	4.7478	0.905385	-2.39065	-3.21467	0.00375	0.983395
ENSG00000168685	IL7R	5:35852694-35879603	Protein_coding_from_Ensembl	MCF-7 cells	MCF-7 spheroid cells	0.797138	3.92457	2.29963	2.80486	0.0101	0.983395
ENSG00000168374	ARF4	3:57571362-57600927	Protein_coding_from_Ensembl	MCF-7 cells	MCF-7 spheroid cells	47.2235	151.1	1.67793	2.74625	0.0064	0.983395
ENSG00000277075	HIST1H2A	6:26216974-26217483	Protein_coding_from_Ensembl	MCF-7 cells	MCF-7 spheroid cells	736.596	189.901	-1.95563	-3.42598	0.00105	0.983395
ENSG00000171551	ECEL1	2:232479826-232487828	Protein_coding_from_Ensembl	MCF-7 cells	MCF-7 spheroid cells	111.766	34.1307	-1.71134	-2.84238	0.006	0.983395
ENSG00000169330	KIAA1024	15:79432515-79472290	Protein_coding_from_Ensembl	MCF-7 cells	MCF-7 spheroid cells	0.529571	2.69426	2.347	2.71629	0.0151	0.983395
ENSG00000168874	ATOH8	2:85751343-85788066	Protein_coding_from_Ensembl	MCF-7 cells	MCF-7 spheroid cells	12.4172	0.869434	-3.83612	-4.25763	0.0012	0.983395
ENSG00000244405	ETV5	3:186046307-186362237	Protein_coding_from_Ensembl	MCF-7 cells	MCF-7 spheroid cells	5.9112	16.3032	1.46363	2.23401	0.02565	0.983395
ENSG00000169710	FASN	17:82078332-82098332	Protein_coding_from_Ensembl	MCF-7 cells	MCF-7 spheroid cells	14.8299	89.2026	2.58857	4.21127	5.00E-05	0.174136
ENSG00000171928	TVP23B	17:18780994-18806714	Protein_coding_from_Ensembl	MCF-7 cells	MCF-7 spheroid cells	7.95641	27.9402	1.81215	2.06995	0.0472	0.983395
ENSG00000169249	ZRSR2	X:15790471-15823260	Protein_coding_from_Ensembl	MCF-7 cells	MCF-7 spheroid cells	9.99441	30.681	1.61815	2.38675	0.0214	0.983395
ENSG00000139318	DUSP6	12:89347231-89353271	Protein_coding_from_Ensembl	MCF-7 cells	MCF-7 spheroid cells	0.496843	10.3433	4.37976	3.30053	0.03395	0.983395
ENSG00000173320	STOX2	4:183853430-184023526	Protein_coding_from_Ensembl	MCF-7 cells	MCF-7 spheroid cells	9.61504	3.97666	-1.27374	-2.08771	0.0325	0.983395
ENSG00000173546	CSPG4	15:75674321-75712848	Protein_coding_from_Ensembl	MCF-7 cells	MCF-7 spheroid cells	4.86321	1.34159	-1.85797	-2.63504	0.0122	0.983395
ENSG00000168404	MLKL	16:74671854-74700960	Protein_coding_from_Ensembl	MCF-7 cells	MCF-7 spheroid cells	6.3226	17.6051	1.4774	2.12814	0.03535	0.983395
ENSG00000010704	HFE	6:26043226-26123926	Protein_coding_from_Ensembl	MCF-7 cells	MCF-7 spheroid cells	1.57839	0	-1.3664705	NA	0.0281	0.983395
ENSG00000173391	OLR1	12:10158300-10191801	Protein_coding_from_Ensembl	MCF-7 cells	MCF-7 spheroid cells	39.3978	14.9296	-1.39994	-2.25668	0.0224	0.983395
ENSG00000174951	FUT1	19:48748010-48755390	Protein_coding_from_Ensembl	MCF-7 cells	MCF-7 spheroid cells	3.05363	0.884996	-1.78678	-2.11972	0.0383	0.983395
ENSG00000100285	NEFH	22:29480219-29491390	Protein_coding_from_Ensembl	MCF-7 cells	MCF-7 spheroid cells	8.74619	2.13056	-2.03742	-2.85062	0.00595	0.983395
ENSG00000173706	HEG1	3:124965709-125055958	Protein_coding_from_Ensembl	MCF-7 cells	MCF-7 spheroid cells	11.1925	4.17248	-1.42356	-2.33948	0.0193	0.983395
ENSG00000136205	TNS3	7:47275153-47582558	Protein_coding_from_Ensembl	MCF-7 cells	MCF-7 spheroid cells	11.0673	2.31852	-2.25502	-3.40838	0.0019	0.983395
ENSG00000128422	KRT17	17:41619436-41624842	Protein_coding_from_Ensembl	MCF-7 cells	MCF-7 spheroid cells	133.653	544.408	2.02619	3.17714	0.00165	0.983395
ENSG00000173083	HPSE	4:83292460-83335153	Protein_coding_from_Ensembl	MCF-7 cells	MCF-7 spheroid cells	5.157	0.991052	-2.3795	-2.84597	0.01115	0.983395
ENSG00000168453	HR	8:22114414-22133384	Protein_coding_from_Ensembl	MCF-7 cells	MCF-7 spheroid cells	5.97877	0.467607	-3.67648	-3.7996	0.00485	0.983395
ENSG00000167767	KRT80	12:52168995-52192000	Protein_coding_from_Ensembl	MCF-7 cells	MCF-7 spheroid cells	12.1351	1.92027	-2.6598	-2.61853	0.01475	0.983395
ENSG00000181649	PHLDA2	11:2928272-2929455	Protein_coding_from_Ensembl	MCF-7 cells	MCF-7 spheroid cells	15.4678	79.1369	2.35508	3.60653	0.00135	0.983395
ENSG00000066735	KIF26A	14:104138722-10418089	Protein_coding_from_Ensembl	MCF-7 cells	MCF-7 spheroid cells	5.67522	1.52004	-1.90056	-2.69814	0.0119	0.983395
ENSG00000166147	FBN1	15:48408305-48645849	Protein_coding_from_Ensembl	MCF-7 cells	MCF-7 spheroid cells	25.8382	8.66319	-1.57653	-2.45204	0.014	0.983395
ENSG00000153714	LURAP1L	9:12685438-12822131	Protein_coding_from_Ensembl	MCF-7 cells	MCF-7 spheroid cells	7.35669	22.6633	1.62323	2.26533	0.02555	0.983395
ENSG00000120616	EPC1	10:32266288-32378798	Protein_coding_from_Ensembl	MCF-7 cells	MCF-7 spheroid cells	0.000169	2.13875	13.6244	0.11386	0.0421	0.983395
ENSG00000164403	SHROOM1	5:132822140-132830898	Protein_coding_from_Ensembl	MCF-7 cells	MCF-7 spheroid cells	0.813055	0.000104	-12.9332	-0.05129	0.03285	0.983395
ENSG00000099256	PRTFDC1	10:24848606-24952604	Protein_coding_from_Ensembl	MCF-7 cells	MCF-7 spheroid cells	14.9042	2.59405	-2.52244	-2.98176	0.0113	0.983395
ENSG00000153071	DAB2	5:39284261-39462300	Protein_coding_from_Ensembl	MCF-7 cells	MCF-7 spheroid cells	29.4215	10.0063	-1.55595	-2.25771	0.02645	0.983395
ENSG00000067836	ROGDI	16:4796967-4802950	Protein_coding_from_Ensembl	MCF-7 cells	MCF-7 spheroid cells	5.21918	5.83E-05	-16.4507	-0.01882	0.0346	0.983395
ENSG00000111799	COL12A1	6:75084325-75206051	Protein_coding_from_Ensembl	MCF-7 cells	MCF-7 spheroid cells	45.0866	5.52876	-3.02767	-4.97725	5.00E-05	0.174136
ENSG00000005238	FAM214B	9:35104111-35116341	Protein_coding_from_Ensembl	MCF-7 cells	MCF-7 spheroid cells	8.10E-05	1.17012	13.8184	0.040575	0.04995	0.983395
ENSG00000189306	RRP7A	22:42500578-42519802	Protein_coding_from_Ensembl	MCF-7 cells	MCF-7 spheroid cells	12.6969	5.24793	-1.27466	-2.02411	0.04145	0.983395
ENSG00000004866	ST7	7:116952445-117230103	Protein_coding_from_Ensembl	MCF-7 cells	MCF-7 spheroid cells	5.02331	0.000178	-14.788	-0.06515	0.0436	0.983395
ENSG00000107554	DNMBP	10:99875576-100009919	Protein_coding_from_Ensembl	MCF-7 cells	MCF-7 spheroid cells	9.47037	4.20134	-1.17257	-1.93949	0.04205	0.983395
ENSG00000044574	HSPA5	9:125234852-125241330	Protein_coding_from_Ensembl	MCF-7 cells	MCF-7 spheroid cells	80.9268	191.135	1.2399	2.12896	0.03265	0.983395
ENSG00000112972	HMGCS1	5:43287600-43313512	Protein_coding_from_Ensembl	MCF-7 cells	MCF-7 spheroid cells	5.27265	29.4475	2.48155	2.75865	0.00665	0.983395
ENSG00000186831	KRT17P2	17:18426860-18431848	OtherRNA_from_Ensembl	MCF-7 cells	MCF-7 spheroid cells	1.31803	5.4883	2.05798	2.10831	0.04635	0.983395
ENSG00000197766	CFD	19:859642-863630	Protein_coding_from_Ensembl	MCF-7 cells	MCF-7 spheroid cells	56.9109	19.4354	-1.55002	-2.50578	0.01195	0.983395
ENSG00000086061	DNAJA1	9:33025210-33039907	Protein_coding_from_Ensembl	MCF-7 cells	MCF-7 spheroid cells	79.7941	188.342	1.239	2.16798	0.031	0.983395
ENSG00000100311	PDGFB	22:39223358-39244751	Protein_coding_from_Ensembl	MCF-7 cells	MCF-7 spheroid cells	3.90131	0.931412	-2.06647	-2.28256	0.03355	0.983395
ENSG00000184260	HIST2H2A	1:149886974-149887364	Protein_coding_from_Ensembl	MCF-7 cells	MCF-7 spheroid cells	1064.57	412.889	-1.36645	-2.42156	0.0198	0.983395
ENSG00000135480	KRT7	12:52232519-52309163	Protein_coding_from_Ensembl	MCF-7 cells	MCF-7 spheroid cells	325.881	84.2914	-1.95089	-2.89933	0.0052	0.983395
ENSG00000087086	FTL	19:48965300-48966878	Protein_coding_from_Ensembl	MCF-7 cells	MCF-7 spheroid cells	611.689	2340.54	1.93597	3.09781	0.0029	0.983395
ENSG00000182585	EPGN	4:74308472-74315307	Protein_coding_from_Ensembl	MCF-7 cells	MCF-7 spheroid cells	3.03453	20.7569	2.77405	2.24992	0.0385	0.983395
ENSG00000185432	METTL7A	12:50923471-50932517	Protein_coding_from_Ensembl	MCF-7 cells	MCF-7 spheroid cells	16.8872	5.26223	-1.68218	-2.52279	0.01435	0.983395
ENSG00000276368	HIST1H2A	6:27814353-27814740	Protein_coding_from_Ensembl	MCF-7 cells	MCF-7 spheroid cells	284.768	53.9853	-2.39915	-3.61007	0.00075	0.983395
ENSG00000182459	TEX19	17:82359246-82363776	Protein_coding_from_Ensembl	MCF-7 cells	MCF-7 spheroid cells	4.4396	14.8766	1.74454	2.43718	0.01665	0.983395
ENSG00000183111	ARHGEF3	5:149551946-149634968	Protein_coding_from_Ensembl	MCF-7 cells	MCF-7 spheroid cells	1.91565	0.454942	-2.07408	-2.18342	0.0415	0.983395
ENSG00000132906	CASP9	1:15456727-15526534	Protein_coding_from_Ensembl	MCF-7 cells	MCF-7 spheroid cells	24.8862	6.44883	-1.94823	-2.38215	0.0206	0.983395
ENSG00000167815	PRDX2	19:12763002-12874951	Protein_coding_from_Ensembl	MCF-7 cells	MCF-7 spheroid cells	4.12082	0	-2.35637485	NA	0.0475	0.983395
ENSG00000109066	TMEM104	17:74776482-74839779	Protein_coding_from_Ensembl	MCF-7 cells	MCF-7 spheroid cells	1.12943	4.4811	1.98825	2.49674	0.0211	0.983395
ENSG00000165629	ATP5C1	10:7788128-7807815	Protein_coding_from_Ensembl	MCF-7 cells	MCF-7 spheroid cells	0	12.0843	3.709764838	NA	0.0421	0.983395
ENSG00000179119	SPTY2D1	11:18599786-18634791	Protein_coding_from_Ensembl	MCF-7 cells	MCF-7 spheroid cells	3.68461	11.1035	1.59144	2.31133	0.0215	0.983395
ENSG00000197780	TAF13	1:109062485-109076002	Protein_coding_from_Ensembl	MCF-7 cells	MCF-7 spheroid cells	24.4669	73.2773	1.58253	2.16797	0.0311	0.983395
ENSG00000124107	SLPI	20:45252238-45254564	Protein_coding_from_Ensembl	MCF-7 cells	MCF-7 spheroid cells	139.965	445.294	1.66969	2.94281	0.0052	0.983395
ENSG00000109654	TRIM2	4:153152341-153339320	Protein_coding_from_Ensembl	MCF-7 cells	MCF-7 spheroid cells	8.19894	2.37465	-1.78772	-2.61122	0.011	0.983395
ENSG00000054793	ATP9A	20:51596513-51768634	Protein_coding_from_Ensembl	MCF-7 cells	MCF-7 spheroid cells	7.39429	2.50248	-1.56305	-2.53485	0.01095	0.983395
ENSG00000153234	NR4A2	2:156324431-156342348	Protein_coding_from_Ensembl	MCF-7 cells	MCF-7 spheroid cells	0.977284	9.15429	3.2276	3.38027	0.0083	0.983395
ENSG00000124635	HIST1H2E	6:27125896-27132750	Protein_coding_from_Ensembl	MCF-7 cells	MCF-7 spheroid cells	455.948	194.604	-1.22833	-2.04508	0.0351	0.983395
ENSG00000188505	NCCRP1	19:39196960-39201884	Protein_coding_from_Ensembl	MCF-7 cells	MCF-7 spheroid cells	2.76915	0.202815	-3.77121	-3.17142	0.0239	0.983395
ENSG00000174928	C3orf33	3:155762616-155806351	Protein_coding_from_Ensembl	MCF-7 cells	MCF-7 spheroid cells	0.798848	0.000132	-12.5686	-0.03558	0.0335	0.983395

ENSG00000135047	CTSL	9:87725518-87731393	Protein_coding_from_Ensembl	MCF-7 cells	MCF-7 spheroid cells	102.736	646.681	2.65411	3.78538	0.00025	0.505556
ENSG00000186480	INSIG1	7:155297775-155310235	Protein_coding_from_Ensembl	MCF-7 cells	MCF-7 spheroid cells	6.64955	88.2385	3.73008	5.84727	5.00E-05	0.174136
ENSG00000041982	TNC	9:115019577-115118257	Protein_coding_from_Ensembl	MCF-7 cells	MCF-7 spheroid cells	0.234899	3.28961	3.80781	3.47695	0.00795	0.983395
ENSG00000151500	THYN1	11:134248278-13425337(	Protein_coding_from_Ensembl	MCF-7 cells	MCF-7 spheroid cells	0.00061	4.32063	12.7892	0.111194	0.04935	0.983395
ENSG00000137699	TRIM29	11:120111274-12018552{	Protein_coding_from_Ensembl	MCF-7 cells	MCF-7 spheroid cells	16.3351	0.617821	-4.72464	-3.3034	0.03275	0.983395
ENSG00000145220	LYAR	4:4267700-4290169	Protein_coding_from_Ensembl	MCF-7 cells	MCF-7 spheroid cells	28.2698	72.3586	1.3559	2.22693	0.02575	0.983395
ENSG00000118898	PPL	16:4882506-5097808	Protein_coding_from_Ensembl	MCF-7 cells	MCF-7 spheroid cells	30.1994	8.12301	-1.89443	-2.67579	0.01025	0.983395
ENSG00000102547	CAB39L	13:49308649-49444126	Protein_coding_from_Ensembl	MCF-7 cells	MCF-7 spheroid cells	3.15E-05	0.891297	14.7873	0.018873	0.04325	0.983395
ENSG00000164045	CDC25A	3:48157145-48188402	Protein_coding_from_Ensembl	MCF-7 cells	MCF-7 spheroid cells	0.00012	1.09194	13.1548	0.069827	0.04115	0.983395
ENSG00000115252	PDE1A	2:182140035-182523192	Protein_coding_from_Ensembl	MCF-7 cells	MCF-7 spheroid cells	20.3229	3.81499	-2.41335	-2.55448	0.01865	0.983395
ENSG00000130707	ASS1	9:130444928-130501274	Protein_coding_from_Ensembl	MCF-7 cells	MCF-7 spheroid cells	757.752	259.431	-1.54637	-2.30496	0.02155	0.983395
ENSG00000166401	SERPINB8	18:63949300-64005667	Protein_coding_from_Ensembl	MCF-7 cells	MCF-7 spheroid cells	4.47E-05	1.04412	14.5107	0.026494	0.0469	0.983395
ENSG00000096384	HSP90AB1	6:44246165-44253888	Protein_coding_from_Ensembl	MCF-7 cells	MCF-7 spheroid cells	0	6.5979	2.925600724	NA	0.04705	0.983395
ENSG00000122566	HNRNPA2	7:26189926-26213356	Protein_coding_from_Ensembl	MCF-7 cells	MCF-7 spheroid cells	0	1.58038	1.36758354	NA	0.0465	0.983395
ENSG00000168079	SCARA5	8:27869881-27992727	Protein_coding_from_Ensembl	MCF-7 cells	MCF-7 spheroid cells	2.9082	0.602174	-2.27188	-2.448	0.02745	0.983395
ENSG00000197238	HIST1H4J	6:27824107-27824480	Protein_coding_from_Ensembl	MCF-7 cells	MCF-7 spheroid cells	111.828	35.6458	-1.64948	-2.22728	0.0301	0.983395
ENSG00000197405	C5AR1	19:47290022-47322066	Protein_coding_from_Ensembl	MCF-7 cells	MCF-7 spheroid cells	3.50468	0.277758	-3.65738	-3.41355	0.0164	0.983395
ENSG00000187720	THSD4	15:71096951-71783383	Protein_coding_from_Ensembl	MCF-7 cells	MCF-7 spheroid cells	3.08828	9.95215	1.6882	2.54545	0.01185	0.983395
ENSG00000172893	DHCR7	11:71428192-71452868	Protein_coding_from_Ensembl	MCF-7 cells	MCF-7 spheroid cells	3.22984	25.0239	2.95377	3.76396	0.00175	0.983395
ENSG00000119899	SLC17A5	6:73593378-73654155	Protein_coding_from_Ensembl	MCF-7 cells	MCF-7 spheroid cells	8.14179	20.2462	1.31424	2.18832	0.02965	0.983395
ENSG00000101166	PRELID3B	20:59033144-59042909	Protein_coding_from_Ensembl	MCF-7 cells	MCF-7 spheroid cells	240.092	88.4378	-1.44085	-2.39266	0.01855	0.983395
ENSG00000126391	FRMD8	11:65386598-65413525	Protein_coding_from_Ensembl	MCF-7 cells	MCF-7 spheroid cells	0.000197	7.68064	15.2473	0.127773	0.0485	0.983395
ENSG00000196878	LAMB3	1:209614869-209652466	Protein_coding_from_Ensembl	MCF-7 cells	MCF-7 spheroid cells	9.72023	30.7681	1.66237	2.6018	0.01005	0.983395
ENSG00000277224	HIST1H2E	6:26199519-26200715	Protein_coding_from_Ensembl	MCF-7 cells	MCF-7 spheroid cells	440.893	113.9	-1.95266	-3.42036	0.0015	0.983395
ENSG00000198681	MAGEA1	X:153179284-153183880	Protein_coding_from_Ensembl	MCF-7 cells	MCF-7 spheroid cells	0.299725	2.86362	3.25613	2.86794	0.02435	0.983395
ENSG00000096060	FKBP5	6:35573584-35728583	Protein_coding_from_Ensembl	MCF-7 cells	MCF-7 spheroid cells	15.8629	6.12303	-1.37333	-2.14897	0.0302	0.983395
ENSG00000072422	RHOBTB1	10:60869437-61001440	Protein_coding_from_Ensembl	MCF-7 cells	MCF-7 spheroid cells	8.24555	2.02467	-2.02593	-2.26009	0.04475	0.983395
ENSG00000196747	HIST1H2A	6:27808198-27808701	Protein_coding_from_Ensembl	MCF-7 cells	MCF-7 spheroid cells	397.323	116.458	-1.77051	-3.044	0.0036	0.983395
ENSG00000225697	SLC26A6	3:48625722-48635493	Protein_coding_from_Ensembl	MCF-7 cells	MCF-7 spheroid cells	0.000211	2.16203	13.3243	0.091124	0.04975	0.983395
ENSG00000164930	FZD6	8:103121031-103332866	Protein_coding_from_Ensembl	MCF-7 cells	MCF-7 spheroid cells	24.3835	8.20693	-1.57099	-2.32323	0.0248	0.983395
ENSG00000196177	ACADSB	10:123008978-12305831	Protein_coding_from_Ensembl	MCF-7 cells	MCF-7 spheroid cells	4.45127	0.701344	-2.66602	-3.30291	0.0041	0.983395
ENSG00000138347	MYPN	10:68106116-68212017	Protein_coding_from_Ensembl	MCF-7 cells	MCF-7 spheroid cells	27.2404	4.92882	-2.46643	-3.39648	0.00125	0.983395
ENSG00000068903	SIRT2	19:38878554-38908893	Protein_coding_from_Ensembl	MCF-7 cells	MCF-7 spheroid cells	0.000189	2.63574	13.7687	0.057325	0.02495	0.983395
ENSG00000172572	PDE3A	12:20361731-20684381	Protein_coding_from_Ensembl	MCF-7 cells	MCF-7 spheroid cells	41.3777	15.7588	-1.39269	-2.25381	0.02655	0.983395
ENSG00000196787	HIST1H2A	6:27133041-27135291	Protein_coding_from_Ensembl	MCF-7 cells	MCF-7 spheroid cells	150.711	52.6466	-1.51737	-2.68651	0.0076	0.983395
ENSG00000278677	HIST1H2A	6:27890381-27893149	Protein_coding_from_Ensembl	MCF-7 cells	MCF-7 spheroid cells	958.917	306.463	-1.64569	-2.71661	0.00615	0.983395
ENSG00000072274	TFRC	3:196027182-196082189	Protein_coding_from_Ensembl	MCF-7 cells	MCF-7 spheroid cells	254.941	72.7163	-1.80981	-2.79003	0.00725	0.983395
ENSG00000131069	ACSS2	20:34844719-34927962	Protein_coding_from_Ensembl	MCF-7 cells	MCF-7 spheroid cells	4.5911	36.1032	2.97522	4.30478	5.00E-05	0.174136
ENSG00000161011	SQSTM1	5:179797596-179907859	Protein_coding_from_Ensembl	MCF-7 cells	MCF-7 spheroid cells	73.5279	388.229	2.40054	2.66525	0.0127	0.983395
ENSG00000198888	MT-ND1	MT:3306-4262	Protein_coding_from_Ensembl	MCF-7 cells	MCF-7 spheroid cells	182.673	400.562	1.13276	2.0228	0.0448	0.983395
ENSG00000075945	KIFAP3	1:169921325-170085208	Protein_coding_from_Ensembl	MCF-7 cells	MCF-7 spheroid cells	17.0709	6.84469	-1.31848	-2.03923	0.03965	0.983395
ENSG00000188153	COL4A5	X:108439843-108697545	Protein_coding_from_Ensembl	MCF-7 cells	MCF-7 spheroid cells	9.90527	3.3048	-1.58363	-2.11502	0.03925	0.983395
ENSG00000198796	ALPK2	18:58481246-58628957	Protein_coding_from_Ensembl	MCF-7 cells	MCF-7 spheroid cells	5.71496	1.0436	-2.45318	-3.05246	0.0057	0.983395
ENSG00000198879	SFMBT2	10:7158623-7411486	Protein_coding_from_Ensembl	MCF-7 cells	MCF-7 spheroid cells	9.04377	3.51321	-1.36413	-2.15479	0.029	0.983395
ENSG00000165915	SLC39A13	11:47383147-47416501	Protein_coding_from_Ensembl	MCF-7 cells	MCF-7 spheroid cells	0.00023	1.72445	12.8696	0.079868	0.0498	0.983395
ENSG00000116574	RHOU	1:228735076-228746669	Protein_coding_from_Ensembl	MCF-7 cells	MCF-7 spheroid cells	9.47091	2.67506	-1.82393	-2.71123	0.0087	0.983395
ENSG00000182827	ACBD3	1:226144678-226186730	Protein_coding_from_Ensembl	MCF-7 cells	MCF-7 spheroid cells	32.3442	71.4905	1.14424	2.01225	0.0476	0.983395
ENSG00000152104	PTPN14	1:214344171-214552449	Protein_coding_from_Ensembl	MCF-7 cells	MCF-7 spheroid cells	11.6649	5.00905	-1.21957	-1.91564	0.0496	0.983395
ENSG00000123843	C4BPB	1:207088841-207099993	Protein_coding_from_Ensembl	MCF-7 cells	MCF-7 spheroid cells	80.2737	9.99824	-3.00518	-2.57973	0.02945	0.983395
ENSG00000073756	PTGS2	1:186671790-186680427	Protein_coding_from_Ensembl	MCF-7 cells	MCF-7 spheroid cells	1.50085	11.6997	2.96261	3.56343	0.00205	0.983395
ENSG00000162783	IER5	1:181088711-181092899	Protein_coding_from_Ensembl	MCF-7 cells	MCF-7 spheroid cells	5.67207	20.5168	1.85486	3.07411	0.0032	0.983395
ENSG00000162733	DDR2	1:162631372-162787400	Protein_coding_from_Ensembl	MCF-7 cells	MCF-7 spheroid cells	41.444	11.6245	-1.83399	-3.12333	0.0023	0.983395
ENSG00000158769	F11R	1:160995210-161038990	Protein_coding_from_Ensembl	MCF-7 cells	MCF-7 spheroid cells	6.28284	1.97827	-1.66718	-2.23024	0.0302	0.983395
ENSG00000146376	ARHGAP1	6:129576131-129710225	Protein_coding_from_Ensembl	MCF-7 cells	MCF-7 spheroid cells	4.55943	1.28283	-1.82952	-2.40433	0.0193	0.983395
ENSG00000196154	S100A4	1:153543612-153550136	Protein_coding_from_Ensembl	MCF-7 cells	MCF-7 spheroid cells	109.812	32.268	-1.76687	-2.26306	0.03105	0.983395
ENSG00000163220	S100A9	1:153357853-153361027	Protein_coding_from_Ensembl	MCF-7 cells	MCF-7 spheroid cells	0.989831	51.3325	5.69655	5.26093	0.0172	0.983395
ENSG00000203785	SPRR2E	1:153093134-153106184	Protein_coding_from_Ensembl	MCF-7 cells	MCF-7 spheroid cells	0	2.51758	1.814583235	NA	0.0003	0.537334
ENSG00000143416	SELENBP	1:151364301-151372733	Protein_coding_from_Ensembl	MCF-7 cells	MCF-7 spheroid cells	42.7718	13.0368	-1.71407	-2.48571	0.0146	0.983395
ENSG00000278828	HIST1H3H	6:27810063-27811300	Protein_coding_from_Ensembl	MCF-7 cells	MCF-7 spheroid cells	146.512	28.3866	-2.36774	-4.03467	0.0001	0.241112
ENSG00000185515	BRCC3	X:155061621-155156503	Protein_coding_from_Ensembl	MCF-7 cells	MCF-7 spheroid cells	5.55646	0.000313	-14.1153	-0.14925	0.03625	0.983395
ENSG00000143061	IGSF3	1:116574398-116667755	Protein_coding_from_Ensembl	MCF-7 cells	MCF-7 spheroid cells	1.70297	6.89E-05	-14.593	-0.0913	0.0244	0.983395
ENSG00000116815	CD58	1:116514534-116571039	Protein_coding_from_Ensembl	MCF-7 cells	MCF-7 spheroid cells	19.4959	54.3017	1.47783	2.05585	0.04215	0.983395
ENSG00000138166	DUSP5	10:110497837-11051154	Protein_coding_from_Ensembl	MCF-7 cells	MCF-7 spheroid cells	8.4889	88.1611	3.37649	5.4949	5.00E-05	0.174136
ENSG00000107957	SH3PXD2	10:103594026-10385554	Protein_coding_from_Ensembl	MCF-7 cells	MCF-7 spheroid cells	17.6772	5.67783	-1.63848	-2.57991	0.01275	0.983395
ENSG00000214357	NEURL1B	5:172641265-172697720	Protein_coding_from_Ensembl	MCF-7 cells	MCF-7 spheroid cells	1.69762	0.335151	-2.34063	-2.57053	0.0213	0.983395
ENSG00000135338	LCA5	6:79484990-79537458	Protein_coding_from_Ensembl	MCF-7 cells	MCF-7 spheroid cells	1.35266	0.107342	-3.65552	-2.98428	0.04005	0.983395
ENSG00000196924	FLNA	X:154348523-154374638	Protein_coding_from_Ensembl	MCF-7 cells	MCF-7 spheroid cells	100.264	39.6014	-1.34018	-2.00735	0.04445	0.983395
ENSG00000099194	SCD	10:100347123-10036483	Protein_coding_from_Ensembl	MCF-7 cells	MCF-7 spheroid cells	191.754	1900.22	3.30883	3.53494	0.00145	0.983395
ENSG00000079841	RIMS1	6:71886702-72403143	Protein_coding_from_Ensembl	MCF-7 cells	MCF-7 spheroid cells	13.6722	0.666905	-4.35762	-2.93356	0.0473	0.983395
ENSG00000146166	LGSN	6:63275950-63319977	Protein_coding_from_Ensembl	MCF-7 cells	MCF-7 spheroid cells	4.5755	0.349475	-3.71067	-3.89701	0.00505	0.983395
ENSG00000137959	IFI44L	1:78619921-78646145	Protein_coding_from_Ensembl	MCF-7 cells	MCF-7 spheroid cells	0.574033	6.71201	3.54754	3.89746	0.00405	0.983395
ENSG00000124203	ZNF831	20:59191019-59259113	Protein_coding_from_Ensembl	MCF-7 cells	MCF-7 spheroid cells	11.8759	0.992611	-3.58066	-5.3196	5.00E-05	0.174136
ENSG00000087460	GNAS	20:58818918-58911192	Protein_coding_from_Ensembl	MCF-7 cells	MCF-7 spheroid cells	7.14732	0	-3.02632557	NA	0.04105	0.983395
ENSG00000182463	TSHZ2	20:52972406-53504314	Protein_coding_from_Ensembl	MCF-7 cells	MCF-7 spheroid cells	2.20971	0.544298	-2.02139	-2.17764	0.0379	0.983395
ENSG00000130558	OLFM1	9:135075242-135121179	Protein_coding_from_Ensembl	MCF-7 cells	MCF-7 spheroid cells	0.337462	3.0111	3.15749	2.7821	0.0271	0.983395
ENSG00000117016	RIMS3	1:40620678-40665657	Protein_coding_from_Ensembl	MCF-7 cells	MCF-7 spheroid cells	1.04008	0.192821	-2.43136	-2.56455	0.0198	0.983395
ENSG00000064205	WISP2	20:44656450-44810338	Protein_coding_from_Ensembl	MCF-7 cells	MCF-7 spheroid cells	74.4631	4.34558	-4.09891	-4.25612	0.0004	0.642964
ENSG00000149596	JPH2	20:44111694-44187578	Protein_coding_from_Ensembl	MCF-7 cells	MCF-7 spheroid cells	1.69628	0.13614	-3.63921	-3.36462	0.01285	0.983395
ENSG00000122863	CHST3	10:71964364-72013564	Protein_coding_from_Ensembl	MCF-7 cells	MCF-7 spheroid cells	15.3031	6.05755	-1.33702	-2.27773	0.0266	0.983395
ENSG00000136877	FPGS	9:127794596-127814327	Protein_coding_from_Ensembl	MCF-7 cells	MCF-7 spheroid cells	3.71065	0.000188	-14.2681	-0.07126	0.0347	0.983395
ENSG00000244122	UGT1A7	2:233617644-233773310	Protein_coding_from_Ensembl	MCF-7 cells	MCF-7 spheroid cells	0.152143	5.48799	5.17278	3.91308	0.0354	0.983395
ENSG00000185585	OLFML2A	9:124777157-124814885	Protein_coding_from_Ensembl	MCF-7 cells	MCF-7 spheroid cells	2.48129	0.18882	-3.71601	-4.00423	0.0023	0.983395
ENSG00000156711	MAPK13	6:36127808-36144524	Protein_coding_from_Ensembl	MCF-7 cells	MCF-7 spheroid cells	0.427453	1.81085	2.08283	2.28166	0.0366	0.983395
ENSG00000122870	BICC1	10:58513139-58831437	Protein_coding_from_Ensembl	MCF-7 cells	MCF-7 spheroid cells	9.6482	2.88809	-1.74014	-2.64349	0.01225	0.983395
ENSG00000181773	GPR3	1:27392643-27395814	Protein_coding_from_Ensembl	MCF-7 cells	MCF-7 spheroid cells	0.270853	1.67171	2.62574	2.32883	0.04925	0.983395
ENSG00000181634	TNFSF15	9:114784634-114806126	Protein_coding_from_Ensembl	MCF-7 cells	MCF-7 spheroid cells	0.07048	2.3778	5.07628	4.78637	0.013	0.983395
ENSG00000148225	WDR31	9:113313221-113340298	Protein_coding_from_Ensembl	MCF-7 cells	MCF-7 spheroid cells	2.61746	0.481391	-2.44288	-2.25819	0.04925	0.983395
ENSG00000101019	UQCC1	20:35302565-35412141	Protein_coding_from_Ensembl	MCF-7 cells	MCF-7 spheroid cells	1.49954	0.00059	-11.3125	-0.12056	0.0421	0.983395
ENSG00000165124	SVEP1	9:110365250-110579880	Protein_coding_from_Ensembl	MCF-7 cells	MCF-7 spheroid cells	11.7328	2.39203	-2.29423	-3.26477	0.00235	0.983395
ENSG00000117318	ID3	1:23557917-23559794	Protein_coding_from_Ensembl	MCF-7 cells	MCF-7 spheroid cells	109.457	35.0188	-1.64417	-2.72816	0.00735	0.983395
ENSG00000204217	BMPR2	2:202376935-202567751	Protein_coding_from_Ensembl	MCF-7 cells	MCF-7 spheroid cells	3.00206	1.13113	-1.40819	-2.07121	0.0426	0.983395
ENSG00000138356	AOX1	2:200585867-200677064	Protein_coding_from_Ensembl	MCF-7 cells	MCF-7 spheroid cells	1.87726	0.185768	-3.33706	-2.78937	0.03135	0.983395
ENSG00000165029	ABCA1	9:104781001-104928892	Protein_coding_from_Ensembl	MCF-7 cells	MCF-7 spheroid cells	0.063522	0.738187	3.53867	3.25981	0.0195	0.983395
ENSG00000184205	TSPYL2	X:53082366-53088540	Protein_coding_from_Ensembl	MCF-7 cells	MCF-7 spheroid cells	10.215	27.87	1.44802	2.12214	0.03255	0.983395
ENSG00000204386	NEU1	6:31857658-31862906	Protein_coding_from_Ensembl	MCF-7 cells	MCF-7 spheroid cells	9.63405	33.3219	1.79026	2.76089	0.006	0.983395
ENSG00000204388	HSPA1B	6:31827734-31830255	Protein_coding_from_Ensembl	MCF-7 cells	MCF-7 spheroid cells	14.6362	68.3306	2.22299	3.82774	0.0003	0.537334
ENSG00000204389	HSPA1A	6:31815463-31817946	Protein_coding_from_Ensembl	MCF-7 cells	MCF-7 spheroid cells	18.6917	117.855	2.65655	3.37962	0.00125	0.983395
ENSG00000204390	HSPA1L	6:31809618-31815065	Protein_coding_from_Ensembl	MCF-7 cells	MCF-7 spheroid cells	1.30251	6.02512	2.2097	2.6424	0.01295	0.983395
ENSG00000150054	MPP7	10:28050992-28334486	Protein_coding_from_Ensembl	MCF-7 cells	MCF-7 spheroid cells	25.6093	9.97434	-1.36037	-2.18545	0.02695	0.983395
ENSG00000187240	DYNC2H1	11:103109430-10347986	Protein_coding_from_Ensembl	MCF-7 cells	MCF-7 spheroid cells	2.1292	0.181772	-3.55011	-4.09459	0.0025	0.983395
ENSG00000062282	DGAT2	11:75758454-75803415	Protein_coding_from_Ensembl	MCF-7 cells	MCF-7 spheroid cells	1.92374	0.000434	-12.1153	-0.1425	0.0323	0.983395
ENSG00000204637	AC068538	2:100509276-100509612	OtherRNA_from_Ensembl	MCF-7 cells	MCF-7 spheroid cells	0	2.48951	1.803024466	NA	0.01045	0.983395
ENSG00000102265	TIMP1	X:47561099-47619853	Protein_coding_from_Ensembl	MCF-7 cells	MCF-7 spheroid cells	82.2694	556.776	2.75867	2.14011	0.04975	0.983395
ENSG00000136158	SPRY2	13:80335975-80340951	Protein_coding_from_Ensembl	MCF-7 cells	MCF-7 spheroid cells	4.39241	24.4955	2.47943	3.38356	0.00365	0.983395
ENSG00000197279	ZNF165	6:28078791-28089563	Protein_coding_from_Ensembl	MCF-7 cells	MCF-7 spheroid cells	2.14182	8.88999	2.05334	2.49552	0.0165	0.983395

ENSG00000185130	HIST1H2E	6:27807443-27807931	Protein_coding_from_Ensembl	MCF-7 cells	MCF-7 spheroid cells	412.375	135.367	-1.60708	-2.7599	0.00775	0.983395
ENSG00000158406	HIST1H4F	6:26277608-26285638	Protein_coding_from_Ensembl	MCF-7 cells	MCF-7 spheroid cells	436.684	124.236	-1.8135	-2.61999	0.01055	0.983395
ENSG00000158373	HIST1H2E	6:26158145-26184655	Protein_coding_from_Ensembl	MCF-7 cells	MCF-7 spheroid cells	872.756	370.902	-1.23454	-1.95355	0.04925	0.983395
ENSG00000180573	HIST1H2A	6:26124144-26139116	Protein_coding_from_Ensembl	MCF-7 cells	MCF-7 spheroid cells	250.895	44.4146	-2.49798	-3.2663	0.0055	0.983395
ENSG00000125864	BFSP1	20:17493904-17569220	Protein_coding_from_Ensembl	MCF-7 cells	MCF-7 spheroid cells	0.591441	3.58156	2.59828	2.58009	0.0164	0.983395
ENSG00000124942	AHNAK	11:62433541-62556235	Protein_coding_from_Ensembl	MCF-7 cells	MCF-7 spheroid cells	90.9934	32.7223	-1.47549	-2.31049	0.02245	0.983395
ENSG00000111802	TDP2	6:24649976-24667033	Protein_coding_from_Ensembl	MCF-7 cells	MCF-7 spheroid cells	25.67	61.5118	1.26078	2.14737	0.0271	0.983395
ENSG00000049130	KITLG	12:88492792-88598218	Protein_coding_from_Ensembl	MCF-7 cells	MCF-7 spheroid cells	26.775	11.3336	-1.24027	-1.99481	0.0452	0.983395
ENSG00000198947	DMD	X:31097676-33339441	Protein_coding_from_Ensembl	MCF-7 cells	MCF-7 spheroid cells	22.8139	53.5327	1.23051	1.97377	0.0452	0.983395
ENSG00000134463	ECHDC3	10:11742365-11764070	Protein_coding_from_Ensembl	MCF-7 cells	MCF-7 spheroid cells	7.91078	1.33638	-2.56549	-2.79336	0.01325	0.983395
ENSG00000130066	SAT1	X:23783172-23786226	Protein_coding_from_Ensembl	MCF-7 cells	MCF-7 spheroid cells	46.1376	329.851	2.8378	3.44312	0.00075	0.983395
ENSG00000078401	EDN1	6:12290362-12297194	Protein_coding_from_Ensembl	MCF-7 cells	MCF-7 spheroid cells	2.89909	0.467291	-2.63321	-2.45136	0.03675	0.983395
ENSG00000111859	NEDD9	6:11173451-11382348	Protein_coding_from_Ensembl	MCF-7 cells	MCF-7 spheroid cells	14.9384	2.83746	-2.39636	-3.3821	0.0022	0.983395
ENSG00000107201	DDX58	9:32455704-32526324	Protein_coding_from_Ensembl	MCF-7 cells	MCF-7 spheroid cells	0.000114	3.46847	14.898	0.084206	0.03495	0.983395
ENSG00000164292	RHOBTB3	5:95701248-95824383	Protein_coding_from_Ensembl	MCF-7 cells	MCF-7 spheroid cells	43.4425	10.1043	-2.10414	-2.75307	0.0141	0.983395
ENSG00000138778	CENPE	4:103105805-103198409	Protein_coding_from_Ensembl	MCF-7 cells	MCF-7 spheroid cells	18.1796	6.5772	-1.46678	-2.21733	0.02905	0.983395
ENSG00000132256	TRIM5	11:5663556-5938619	Protein_coding_from_Ensembl	MCF-7 cells	MCF-7 spheroid cells	4.30978	12.8848	1.57998	2.04841	0.0461	0.983395
ENSG00000120694	HSPH1	13:31134973-31162388	Protein_coding_from_Ensembl	MCF-7 cells	MCF-7 spheroid cells	8.14227	40.9726	2.33116	2.43572	0.0196	0.983395
ENSG00000159200	RCAN1	21:34513141-34615142	Protein_coding_from_Ensembl	MCF-7 cells	MCF-7 spheroid cells	2.79475	16.6354	2.57347	3.03288	0.00915	0.983395
ENSG00000198743	SLC5A3	21:34073223-34360033	Protein_coding_from_Ensembl	MCF-7 cells	MCF-7 spheroid cells	6.67231	40.0752	2.58645	4.17345	5.00E-05	0.174136
ENSG00000067064	IDI1	10:971145-1049170	Protein_coding_from_Ensembl	MCF-7 cells	MCF-7 spheroid cells	21.1125	229.461	3.44208	3.50761	0.0018	0.983395
ENSG00000227158	AC073621	17:17422680-17423704	OtherRNA_from_Ensembl	MCF-7 cells	MCF-7 spheroid cells	0.388073	3.37533	3.12063	2.4696	0.0473	0.983395
ENSG00000159140	SON	21:33503930-33977691	Protein_coding_from_Ensembl	MCF-7 cells	MCF-7 spheroid cells	0	2.1672	1.663207972	NA	0.04145	0.983395
ENSG00000151849	CENPJ	13:24882283-24922889	Protein_coding_from_Ensembl	MCF-7 cells	MCF-7 spheroid cells	12.0918	28.9851	1.26128	2.07907	0.0396	0.983395
ENSG00000137440	FGFBP1	4:15935568-15938740	Protein_coding_from_Ensembl	MCF-7 cells	MCF-7 spheroid cells	0.44451	4.77307	3.42463	3.13488	0.02185	0.983395
ENSG00000131370	SH3BP5	3:15254183-15341368	Protein_coding_from_Ensembl	MCF-7 cells	MCF-7 spheroid cells	23.9305	4.97152	-2.26709	-2.59692	0.01795	0.983395
ENSG00000130147	SH3BP4	2:234951972-235055714	Protein_coding_from_Ensembl	MCF-7 cells	MCF-7 spheroid cells	11.1616	4.18845	-1.41405	-2.24632	0.02505	0.983395
ENSG00000213145	CRIP1	14:105486316-10549957	Protein_coding_from_Ensembl	MCF-7 cells	MCF-7 spheroid cells	195.848	52.5847	-1.89702	-2.64685	0.0087	0.983395
ENSG00000198873	GRK5	10:119207588-11945974	Protein_coding_from_Ensembl	MCF-7 cells	MCF-7 spheroid cells	10.4669	3.58856	-1.54435	-2.14653	0.03395	0.983395
ENSG00000171204	TMEM126	11:85628572-85636539	Protein_coding_from_Ensembl	MCF-7 cells	MCF-7 spheroid cells	4.0386	0.000439	-13.1661	-0.07491	0.03535	0.983395
ENSG00000105974	CAV1	7:116524784-116561184	Protein_coding_from_Ensembl	MCF-7 cells	MCF-7 spheroid cells	25.5553	4.00109	-2.67516	-3.19888	0.0029	0.983395
ENSG00000110195	FOLR1	11:72163321-72209241	Protein_coding_from_Ensembl	MCF-7 cells	MCF-7 spheroid cells	407.674	1333.57	1.70981	2.81485	0.00555	0.983395
ENSG00000173227	SYT12	11:67006777-67050863	Protein_coding_from_Ensembl	MCF-7 cells	MCF-7 spheroid cells	7.16075	2.39464	-1.5803	-2.03241	0.0455	0.983395
ENSG00000131737	KRT34	17:41377649-41382403	Protein_coding_from_Ensembl	MCF-7 cells	MCF-7 spheroid cells	0	1.85885	1.515434926	NA	5.00E-05	0.174136
ENSG00000196159	FAT4	4:125316398-125492932	Protein_coding_from_Ensembl	MCF-7 cells	MCF-7 spheroid cells	2.75827	0.608053	-2.18149	-3.10185	0.0043	0.983395
ENSG00000167767	KRT80	12:52168995-52192000	Protein_coding_from_Ensembl	MCF-7 cells	MCF-7 spheroid cells	20.0173	1.44898	-3.78814	-3.80754	0.00265	0.983395
ENSG00000079974	RABL2B	22:50756947-50801309	Protein_coding_from_Ensembl	MCF-7 cells	MCF-7 spheroid cells	0.948605	0.000247	-11.9089	-0.0739	0.04665	0.983395
ENSG00000109321	AREG	4:74445133-74455009	Protein_coding_from_Ensembl	MCF-7 cells	MCF-7 spheroid cells	0.674851	12.3106	4.18919	3.7716	0.01885	0.983395
ENSG00000170638	TRABD	22:50185914-50200837	Protein_coding_from_Ensembl	MCF-7 cells	MCF-7 spheroid cells	0.000406	6.07502	13.8707	0.142634	0.04325	0.983395
ENSG00000150527	CTAGE5	14:39230230-39388513	Protein_coding_from_Ensembl	MCF-7 cells	MCF-7 spheroid cells	0.000312	2.68406	13.0687	0.106231	0.04285	0.983395
ENSG00000115738	ID2	2:8666635-8684453	Protein_coding_from_Ensembl	MCF-7 cells	MCF-7 spheroid cells	9.36369	1.13487	-3.04455	-2.87893	0.0217	0.983395
ENSG00000081052	COL4A4	2:227002710-227164113	Protein_coding_from_Ensembl	MCF-7 cells	MCF-7 spheroid cells	6.46057	1.43223	-2.1734	-3.38885	0.0009	0.983395
ENSG00000215464	AP000354.	22:24269345-24270134	OtherRNA_from_Ensembl	MCF-7 cells	MCF-7 spheroid cells	1.18558	0	-1.12801619	NA	0.0126	0.983395
ENSG00000135127	CCDC64	12:119989868-12009449	Protein_coding_from_Ensembl	MCF-7 cells	MCF-7 spheroid cells	1.75599	9.97794	2.50646	3.14918	0.0054	0.983395
ENSG00000104081	BMF	15:40087889-40108892	Protein_coding_from_Ensembl	MCF-7 cells	MCF-7 spheroid cells	2.67203	0.686264	-1.9611	-2.26278	0.0265	0.983395
ENSG00000105976	MET	7:116672389-116798386	Protein_coding_from_Ensembl	MCF-7 cells	MCF-7 spheroid cells	71.424	27.7139	-1.3658	-2.16391	0.03075	0.983395
ENSG00000188536	HBA2	16:172846-173710	Protein_coding_from_Ensembl	MCF-7 cells	MCF-7 spheroid cells	0	1.27958	1.18876804	NA	0.034	0.983395
ENSG00000100296	THOC5	22:29505878-29581337	Protein_coding_from_Ensembl	MCF-7 cells	MCF-7 spheroid cells	6.8868	0	-2.97944006	NA	0.0283	0.983395
ENSG00000181143	MUC16	19:8848843-8981342	Protein_coding_from_Ensembl	MCF-7 cells	MCF-7 spheroid cells	0.893357	0.251102	-1.83096	-2.7256	0.00755	0.983395
ENSG00000175215	CTDSP2	12:57819926-57846739	Protein_coding_from_Ensembl	MCF-7 cells	MCF-7 spheroid cells	26.1319	10.4193	-1.32656	-2.19095	0.02855	0.983395
ENSG00000206538	VGLL3	3:86937968-86991119	Protein_coding_from_Ensembl	MCF-7 cells	MCF-7 spheroid cells	11.0969	1.36846	-3.01953	-4.5544	0.0001	0.241112
ENSG00000172296	SPTLC3	20:13008978-13169103	Protein_coding_from_Ensembl	MCF-7 cells	MCF-7 spheroid cells	1.99154	0.427419	-2.22016	-2.23889	0.039	0.983395
ENSG00000183762	KREMEN1	22:29073077-29168333	Protein_coding_from_Ensembl	MCF-7 cells	MCF-7 spheroid cells	2.73874	0.447417	-2.61382	-2.94254	0.01035	0.983395
ENSG00000131668	BARX1	9:93951621-93955372	Protein_coding_from_Ensembl	MCF-7 cells	MCF-7 spheroid cells	37.161	13.5976	-1.45044	-2.14366	0.0357	0.983395
ENSG00000140416	TPM1	15:63042631-63071915	Protein_coding_from_Ensembl	MCF-7 cells	MCF-7 spheroid cells	49.787	11.1319	-2.16107	-2.94008	0.00495	0.983395
ENSG00000163026	C2orf44	2:24010080-24063681	Protein_coding_from_Ensembl	MCF-7 cells	MCF-7 spheroid cells	7.18E-05	0.785886	13.4182	0.04316	0.0374	0.983395
ENSG00000129353	SLC44A2	19:10602456-10644559	Protein_coding_from_Ensembl	MCF-7 cells	MCF-7 spheroid cells	7.97485	1.22508	-2.70258	-2.37131	0.0431	0.983395
ENSG00000221869	CEBPD	8:47736908-47739086	Protein_coding_from_Ensembl	MCF-7 cells	MCF-7 spheroid cells	32.9203	86.04	1.38603	2.45033	0.019	0.983395
ENSG00000135919	SERPINE2	2:223975111-224039319	Protein_coding_from_Ensembl	MCF-7 cells	MCF-7 spheroid cells	1.78046	24.3815	3.77546	4.18222	0.00145	0.983395
ENSG00000187514	PTMA	2:231706894-231713541	Protein_coding_from_Ensembl	MCF-7 cells	MCF-7 spheroid cells	1.99192	0	-1.5810716	NA	0.02885	0.983395
ENSG00000196843	ARID5A	2:96536742-96552638	Protein_coding_from_Ensembl	MCF-7 cells	MCF-7 spheroid cells	1.56842	5.92E-05	-14.6933	-0.02043	0.01765	0.983395
ENSG00000111271	ACAD10	12:111686055-11181752	Protein_coding_from_Ensembl	MCF-7 cells	MCF-7 spheroid cells	1.39493	8.33E-05	-14.0307	-0.04106	0.03195	0.983395
ENSG00000182585	EPGN	4:74308472-74315307	Protein_coding_from_Ensembl	MCF-7 cells	MCF-7 spheroid cells	3.34503	27.4658	3.03755	3.08977	0.01075	0.983395
ENSG00000149054	ZNF215	11:6926403-6984632	Protein_coding_from_Ensembl	MCF-7 cells	MCF-7 spheroid cells	0.000154	0.991577	12.657	0.076105	0.03845	0.983395
ENSG00000130600	H19	11:1995162-2001470	OtherRNA_from_Ensembl	MCF-7 cells	MCF-7 spheroid cells	13.7756	0.526464	-4.70964	-4.50981	0.0076	0.983395
ENSG00000176485	PLA2G16	11:63573194-63621671	Protein_coding_from_Ensembl	MCF-7 cells	MCF-7 spheroid cells	21.9477	6.55966	-1.74238	-2.08439	0.0404	0.983395
ENSG00000204054	LINC0096	9:129483450-129513686	OtherRNA_from_Ensembl	MCF-7 cells	MCF-7 spheroid cells	7.396	2.83914	-1.38129	-2.04714	0.0441	0.983395
ENSG00000072274	TFRC	3:196027182-196082189	Protein_coding_from_Ensembl	MCF-7 cells	MCF-7 spheroid cells	5.05733	0	-2.59868201	NA	0.0164	0.983395
ENSG00000147650	LRP12	8:104489230-104589024	Protein_coding_from_Ensembl	MCF-7 cells	MCF-7 spheroid cells	0.627509	4.38E-05	-13.8053	-0.03003	0.0283	0.983395
ENSG00000198728	LDB1	10:102107559-10212045	Protein_coding_from_Ensembl	MCF-7 cells	MCF-7 spheroid cells	25.9362	9.91686	-1.38701	-2.07428	0.04065	0.983395
ENSG00000139915	MDGA2	14:46839628-47674954	Protein_coding_from_Ensembl	MCF-7 cells	MCF-7 spheroid cells	1.62152	0.382041	-2.08555	-2.15222	0.0413	0.983395
ENSG00000197608	ZNF841	19:52031378-52095765	Protein_coding_from_Ensembl	MCF-7 cells	MCF-7 spheroid cells	0.000161	1.77281	13.425	0.094745	0.0443	0.983395
ENSG00000063176	SPHK2	19:48615327-48637438	Protein_coding_from_Ensembl	MCF-7 cells	MCF-7 spheroid cells	1.2889	0	-1.19465443	NA	0.048	0.983395
ENSG00000205758	CRYZL1	21:33503930-33977691	Protein_coding_from_Ensembl	MCF-7 cells	MCF-7 spheroid cells	1.44287	0	-1.28857709	NA	0.04735	0.983395
ENSG00000139132	FGD4	12:32399528-32646050	Protein_coding_from_Ensembl	MCF-7 cells	MCF-7 spheroid cells	4.27639	1.48368	-1.52721	-2.05885	0.04255	0.983395
ENSG00000070371	CLTCL1	22:19179472-19291716	Protein_coding_from_Ensembl	MCF-7 cells	MCF-7 spheroid cells	5.82039	2.05713	-1.50048	-2.02724	0.04125	0.983395
ENSG00000166407	LMO1	11:8224303-8268716	Protein_coding_from_Ensembl	MCF-7 cells	MCF-7 spheroid cells	1.26666	0	-1.180568	NA	0.01155	0.983395
ENSG00000237260	AC073069	2:176338636-176339077	OtherRNA_from_Ensembl	MCF-7 cells	MCF-7 spheroid cells	0	1.09153	1.064558692	NA	0.01745	0.983395
ENSG00000229781	AC013444	2:142854094-142855998	OtherRNA_from_Ensembl	MCF-7 cells	MCF-7 spheroid cells	0.342	2.96218	3.11459	2.81485	0.0283	0.983395
ENSG00000133056	PIK3C2B	1:204377849-204494724	Protein_coding_from_Ensembl	MCF-7 cells	MCF-7 spheroid cells	1.32206	0	-1.21540525	NA	0.0483	0.983395
ENSG00000219481	NBPF1	1:16562318-16613562	Protein_coding_from_Ensembl	MCF-7 cells	MCF-7 spheroid cells	7.27611	2.50163	-1.5403	-2.00213	0.0456	0.983395
ENSG00000112972	HMGCS1	5:43287600-43313512	Protein_coding_from_Ensembl	MCF-7 cells	MCF-7 spheroid cells	4.5477	43.5907	3.26081	3.69248	0.00155	0.983395
ENSG00000103381	CPPED1	16:12659798-12804017	Protein_coding_from_Ensembl	MCF-7 cells	MCF-7 spheroid cells	0.000177	5.66958	14.9638	0.061267	0.0271	0.983395
ENSG00000064195	DLX3	17:49990004-49995224	Protein_coding_from_Ensembl	MCF-7 cells	MCF-7 spheroid cells	1.78525	0.281793	-2.66342	-2.35538	0.04625	0.983395
ENSG00000166016	ABTB2	11:34150987-34358008	Protein_coding_from_Ensembl	MCF-7 cells	MCF-7 spheroid cells	4.02001	1.2687	-1.66385	-2.16342	0.03485	0.983395
ENSG00000106392	C1GALT1	7:7156933-7248651	Protein_coding_from_Ensembl	MCF-7 cells	MCF-7 spheroid cells	3.6754	11.0665	1.59023	2.14651	0.0354	0.983395
ENSG00000163682	RPL9	4:39454123-39458948	Protein_coding_from_Ensembl	MCF-7 cells	MCF-7 spheroid cells	0	3.90675	2.294767768	NA	0.0402	0.983395
ENSG00000224324	THAP5P1	7:158623187-158623449	OtherRNA_from_Ensembl	MCF-7 cells	MCF-7 spheroid cells	5.21951	0	-2.63680092	NA	0.00445	0.983395
ENSG00000144381	HSPD1	2:197486580-197553699	Protein_coding_from_Ensembl	MCF-7 cells	MCF-7 spheroid cells	1.34262	0	-1.22812295	NA	0.0444	0.983395
ENSG00000205758	CRYZL1	21:33503930-33977691	Protein_coding_from_Ensembl	MCF-7 cells	MCF-7 spheroid cells	1.34036	0	-1.22673047	NA	0.0366	0.983395
ENSG00000231804	RP11-317F	9:88484851-88485325	OtherRNA_from_Ensembl	MCF-7 cells	MCF-7 spheroid cells	1.33593	0	-1.22399704	NA	0.0089	0.983395
ENSG00000242028	HYPK	15:43726917-43803043	Protein_coding_from_Ensembl	MCF-7 cells	MCF-7 spheroid cells	0	1.02907	1.020818637	NA	0.0352	0.983395
ENSG00000112096	SOD2	6:159669056-159789749	Protein_coding_from_Ensembl	MCF-7 cells	MCF-7 spheroid cells	0	1.83738	1.504559378	NA	0.04325	0.983395
ENSG00000256061	DYX1C1	15:55202965-55508234	Protein_coding_from_Ensembl	MCF-7 cells	MCF-7 spheroid cells	0	1.31669	1.212065008	NA	0.0467	0.983395
ENSG00000176371	ZSCAN2	15:84599433-84627796	Protein_coding_from_Ensembl	MCF-7 cells	MCF-7 spheroid cells	5.17E-05	0.750554	13.8254	0.033056	0.0441	0.983395
ENSG00000151914	DST	6:56457986-56954628	Protein_coding_from_Ensembl	MCF-7 cells	MCF-7 spheroid cells	0	1.9645	1.567788797	NA	0.0457	0.983395
ENSG00000237412	PRSS56	2:232520462-232525716	Protein_coding_from_Ensembl	MCF-7 cells	MCF-7 spheroid cells	69.0679	4.57998	-3.9146	-4.30141	0.00045	0.671668
ENSG00000115677	HDLBP	2:241227263-241354027	Protein_coding_from_Ensembl	MCF-7 cells	MCF-7 spheroid cells	10.2802	0	-3.49572074	NA	0.02505	0.983395
ENSG00000204520	MICA	6:31399783-31477506	Protein_coding_from_Ensembl	MCF-7 cells	MCF-7 spheroid cells	16.1287	57.1868	1.82606	2.34975	0.02855	0.983395
ENSG00000115365	LANCL1	2:210324758-210477652	Protein_coding_from_Ensembl	MCF-7 cells	MCF-7 spheroid cells	49.6584	17.135	-1.53509	-2.23135	0.02775	0.983395
ENSG00000168385	2-Sep	2:241227263-241354027	Protein_coding_from_Ensembl	MCF-7 cells	MCF-7 spheroid cells	0	1.63322	1.396828061	NA	0.03905	0.983395
ENSG00000149483	TMEM138	11:61299450-61369509	Protein_coding_from_Ensembl	MCF-7 cells	MCF-7 spheroid cells	4.38537	0	-2.42904547	NA	0.0386	0.983395
ENSG00000213771	KRT8P37	10:8513686-8515106	OtherRNA_from_Ensembl	MCF-7 cells	MCF-7 spheroid cells	2.69436	0.258138	-3.38373	-2.78158	0.03365	0.983395
ENSG00000234420	ZNF37BP	10:42513509-42552822	OtherRNA_from_Ensembl	MCF-7 cells	MCF-7 spheroid cells	4.89588	1.80812	-1.43708	-2.0796	0.0398	0.983395
ENSG00000070669	ASNS	7:97852117-97872542	Protein_coding_from_Ensembl	MCF-7 cells	MCF-7 spheroid cells	0	6.00866	2.809138639	NA	0.044	0.983395

ENSG00000133103	COG6	13:39655626-39791665	Protein_coding_from_Ensembl	MCF-7 cells	MCF-7 spheroid cells	5.37169	17.5305	1.70642	2.60766	0.01085	0.983395
ENSG00000114942	EEF1B2	2:206114816-206162928	Protein_coding_from_Ensembl	MCF-7 cells	MCF-7 spheroid cells	0	2.33533	1.73782951	NA	0.0455	0.983395
ENSG00000226549	SCDP1	17:20784644-20785725	OtherRNA_from_Ensembl	MCF-7 cells	MCF-7 spheroid cells	8.14298	56.1956	2.78683	3.88783	0.0014	0.983395
ENSG00000177873	ZNF619	3:40477112-40490236	Protein_coding_from_Ensembl	MCF-7 cells	MCF-7 spheroid cells	0.000186	1.2285	12.6859	0.060574	0.03365	0.983395
ENSG00000205726	ITSN1	21:33503930-33977691	Protein_coding_from_Ensembl	MCF-7 cells	MCF-7 spheroid cells	2.31763	0	-1.730153	NA	0.0376	0.983395
ENSG00000244187	TMEM141	9:136791354-136957733	Protein_coding_from_Ensembl	MCF-7 cells	MCF-7 spheroid cells	0	1.32979	1.220199921	NA	0.04145	0.983395
ENSG00000156976	EIF4A2	3:186781779-186807058	Protein_coding_from_Ensembl	MCF-7 cells	MCF-7 spheroid cells	4.9386	0	-2.57012286	NA	0.04355	0.983395
ENSG00000143321	HDGF	1:156742106-156800817	Protein_coding_from_Ensembl	MCF-7 cells	MCF-7 spheroid cells	2.73287	0	-1.90028527	NA	0.047	0.983395
ENSG00000123843	C4BPB	1:207088841-207099993	Protein_coding_from_Ensembl	MCF-7 cells	MCF-7 spheroid cells	34.6197	6.99485	-2.30723	-2.38531	0.0239	0.983395
ENSG00000154310	TNIK	3:171061338-171460408	Protein_coding_from_Ensembl	MCF-7 cells	MCF-7 spheroid cells	0.587542	0.000174	-11.7194	-0.09823	0.04875	0.983395
ENSG00000213128	RPL32P31	17:80542202-80542605	OtherRNA_from_Ensembl	MCF-7 cells	MCF-7 spheroid cells	1.17202	0	-1.11903739	NA	0.0189	0.983395
ENSG00000185658	BRWD1	21:39184175-39323218	Protein_coding_from_Ensembl	MCF-7 cells	MCF-7 spheroid cells	1.32014	0	-1.21421186	NA	0.0495	0.983395
ENSG00000196924	FLNA	X:154348523-154374638	Protein_coding_from_Ensembl	MCF-7 cells	MCF-7 spheroid cells	0	5.94415	2.795798112	NA	0.03685	0.983395
ENSG00000205726	ITSN1	21:33503930-33977691	Protein_coding_from_Ensembl	MCF-7 cells	MCF-7 spheroid cells	0	1.12144	1.085043876	NA	0.04415	0.983395
ENSG00000142541	RPL13A	19:49446297-49492308	Protein_coding_from_Ensembl	MCF-7 cells	MCF-7 spheroid cells	0	3.05409	2.019378117	NA	0.0188	0.983395
ENSG00000142541	RPL13A	19:49446297-49492308	Protein_coding_from_Ensembl	MCF-7 cells	MCF-7 spheroid cells	1.40067	0	-1.2634371	NA	0.04865	0.983395
ENSG00000128595	CALU	7:128739291-128771807	Protein_coding_from_Ensembl	MCF-7 cells	MCF-7 spheroid cells	0	1.89728	1.534699117	NA	0.0496	0.983395
ENSG00000198818	SFT2D1	6:166319727-166342591	Protein_coding_from_Ensembl	MCF-7 cells	MCF-7 spheroid cells	0.955436	0.000158	-12.5628	-0.04718	0.04205	0.983395
ENSG00000153187	HNRNPU	1:244835321-244864542	Protein_coding_from_Ensembl	MCF-7 cells	MCF-7 spheroid cells	0	2.14851	1.65466925	NA	0.0352	0.983395
ENSG00000240031	OR9A3P	7:141862859-141863737	OtherRNA_from_Ensembl	MCF-7 cells	MCF-7 spheroid cells	0	1.05852	1.041607466	NA	0.0051	0.983395
ENSG00000230667	SETSIP	1:92074532-92075441	Protein_coding_from_Ensembl	MCF-7 cells	MCF-7 spheroid cells	0.000221	1.25238	12.4667	0.024545	0.03105	0.983395
ENSG00000196998	WDR45	X:49071155-49101170	Protein_coding_from_Ensembl	MCF-7 cells	MCF-7 spheroid cells	6.71488	0.000464	-13.8199	-0.09053	0.0304	0.983395
ENSG00000142541	RPL13A	19:49446297-49492308	Protein_coding_from_Ensembl	MCF-7 cells	MCF-7 spheroid cells	0	2.91039	1.967312501	NA	0.02905	0.983395
ENSG00000127603	MACF1	1:39081315-39487177	Protein_coding_from_Ensembl	MCF-7 cells	MCF-7 spheroid cells	0	3.62995	2.210996614	NA	0.04755	0.983395
ENSG00000180398	MCFD2	2:46899274-47176601	Protein_coding_from_Ensembl	MCF-7 cells	MCF-7 spheroid cells	0	2.52909	1.819296223	NA	0.0413	0.983395
ENSG00000160710	ADAR	1:154582061-154627999	Protein_coding_from_Ensembl	MCF-7 cells	MCF-7 spheroid cells	0	4.77239	2.529168776	NA	0.03815	0.983395
ENSG00000198242	RPL23A	17:28719392-28743455	Protein_coding_from_Ensembl	MCF-7 cells	MCF-7 spheroid cells	2.20962	0	-1.6824025	NA	0.0463	0.983395
ENSG00000133056	PIK3C2B	1:204377849-204494724	Protein_coding_from_Ensembl	MCF-7 cells	MCF-7 spheroid cells	1.60383	0	-1.38063526	NA	0.0394	0.983395
ENSG00000127022	CANX	5:179678582-179730925	Protein_coding_from_Ensembl	MCF-7 cells	MCF-7 spheroid cells	0	1.08094	1.057235368	NA	0.0438	0.983395
ENSG00000127022	CANX	5:179678582-179730925	Protein_coding_from_Ensembl	MCF-7 cells	MCF-7 spheroid cells	1.35988	0	-1.2387135	NA	0.04835	0.983395
ENSG00000145425	RPS3A	4:151099572-151325632	Protein_coding_from_Ensembl	MCF-7 cells	MCF-7 spheroid cells	2.39168	0	-1.76200006	NA	0.04275	0.983395
ENSG00000087266	SH3BP2	4:2793022-2841098	Protein_coding_from_Ensembl	MCF-7 cells	MCF-7 spheroid cells	4.54857	1.13255	-2.00583	-2.28847	0.033	0.983395
ENSG00000249992	TMEM158	3:45224465-45226278	Protein_coding_from_Ensembl	MCF-7 cells	MCF-7 spheroid cells	1.24364	8.32412	2.74273	3.08605	0.01095	0.983395
ENSG00000204628	GNB2L1	5:181236908-181272307	Protein_coding_from_Ensembl	MCF-7 cells	MCF-7 spheroid cells	0	4.30185	2.406495854	NA	0.02955	0.983395
ENSG00000120725	SIL1	5:138946719-139331677	Protein_coding_from_Ensembl	MCF-7 cells	MCF-7 spheroid cells	0	4.98073	2.580321589	NA	0.04705	0.983395
ENSG00000118985	ELL2	5:95885097-96808100	Protein_coding_from_Ensembl	MCF-7 cells	MCF-7 spheroid cells	0	3.25361	2.088687763	NA	0.02675	0.983395
ENSG00000184985	SORCS2	4:7192537-7742836	Protein_coding_from_Ensembl	MCF-7 cells	MCF-7 spheroid cells	1.68968	0.217366	-2.95855	-2.93152	0.0149	0.983395
ENSG00000169045	HNRNPH1	5:179614177-179634784	Protein_coding_from_Ensembl	MCF-7 cells	MCF-7 spheroid cells	0	4.10651	2.352337631	NA	0.03975	0.983395
ENSG00000081041	CXCL2	4:74097034-74099293	Protein_coding_from_Ensembl	MCF-7 cells	MCF-7 spheroid cells	3.10037	21.3249	2.78203	3.13934	0.0064	0.983395
ENSG00000112200	ZNF451	6:57086843-57222314	Protein_coding_from_Ensembl	MCF-7 cells	MCF-7 spheroid cells	3.21053	0	-2.07400184	NA	0.04825	0.983395
ENSG00000153113	CAST	5:95885097-96808100	Protein_coding_from_Ensembl	MCF-7 cells	MCF-7 spheroid cells	1.14928	0	-1.10385344	NA	0.0382	0.983395
ENSG00000175426	PCSK1	5:95885097-96808100	Protein_coding_from_Ensembl	MCF-7 cells	MCF-7 spheroid cells	1.96036	0	-1.56577263	NA	0.03775	0.983395
ENSG00000127022	CANX	5:179678582-179730925	Protein_coding_from_Ensembl	MCF-7 cells	MCF-7 spheroid cells	0	1.64993	1.40595425	NA	0.0448	0.983395
ENSG00000145293	ENOPH1	4:82430561-82461091	Protein_coding_from_Ensembl	MCF-7 cells	MCF-7 spheroid cells	5.79537	0.000192	-14.8843	-0.06104	0.03745	0.983395
ENSG00000153113	CAST	5:95885097-96808100	Protein_coding_from_Ensembl	MCF-7 cells	MCF-7 spheroid cells	0	3.62078	2.208136403	NA	0.0429	0.983395
ENSG00000161011	SQSTM1	5:179797596-179907859	Protein_coding_from_Ensembl	MCF-7 cells	MCF-7 spheroid cells	0	1.95899	1.565104821	NA	0.0469	0.983395
ENSG00000164171	ITGA2	5:52675192-53094779	Protein_coding_from_Ensembl	MCF-7 cells	MCF-7 spheroid cells	0	1.20233	1.13903066	NA	0.02365	0.983395
ENSG00000064042	LIMCH1	4:41359606-41700044	Protein_coding_from_Ensembl	MCF-7 cells	MCF-7 spheroid cells	0	2.47117	1.795422023	NA	0.0436	0.983395
ENSG00000218336	TENM3	4:181874437-182803024	Protein_coding_from_Ensembl	MCF-7 cells	MCF-7 spheroid cells	6.91927	1.88892	-1.87306	-2.55837	0.014	0.983395
ENSG00000138674	SEC31A	4:82818660-83012926	Protein_coding_from_Ensembl	MCF-7 cells	MCF-7 spheroid cells	0	5.31096	2.657859479	NA	0.03915	0.983395
ENSG00000169045	HNRNPH1	5:179614177-179634784	Protein_coding_from_Ensembl	MCF-7 cells	MCF-7 spheroid cells	0	2.18707	1.672230706	NA	0.04585	0.983395
ENSG00000153113	CAST	5:95885097-96808100	Protein_coding_from_Ensembl	MCF-7 cells	MCF-7 spheroid cells	2.75457	0	-1.90864769	NA	0.03425	0.983395
ENSG00000179046	TRIML2	4:188091272-188109603	Protein_coding_from_Ensembl	MCF-7 cells	MCF-7 spheroid cells	10.5092	0.948484	-3.46988	-3.33818	0.0146	0.983395
ENSG00000250816	DCAF13P	5:17065597-17276843	OtherRNA_from_Ensembl	MCF-7 cells	MCF-7 spheroid cells	4.54071	0	-2.47007086	NA	0.0279	0.983395
ENSG00000169045	HNRNPH1	5:179614177-179634784	Protein_coding_from_Ensembl	MCF-7 cells	MCF-7 spheroid cells	0	3.45062	2.154006327	NA	0.0409	0.983395
ENSG00000070756	PABPC1	8:100685815-100722809	Protein_coding_from_Ensembl	MCF-7 cells	MCF-7 spheroid cells	5.93123	0	-2.79311139	NA	0.0255	0.983395
ENSG00000108515	ENO3	17:4945651-4957131	Protein_coding_from_Ensembl	MCF-7 cells	MCF-7 spheroid cells	1.34442	0	-1.22923105	NA	0.04855	0.983395
ENSG00000130477	UNC13A	19:17601327-17688365	Protein_coding_from_Ensembl	MCF-7 cells	MCF-7 spheroid cells	0.271063	5.27565	4.28265	3.88171	0.00435	0.983395
ENSG00000164924	YWHAZ	8:100916524-100953388	Protein_coding_from_Ensembl	MCF-7 cells	MCF-7 spheroid cells	0	2.28452	1.71568255	NA	0.0385	0.983395
ENSG00000151914	DST	6:56457986-56954628	Protein_coding_from_Ensembl	MCF-7 cells	MCF-7 spheroid cells	0	1.7388	1.453543918	NA	0.03585	0.983395
ENSG00000169045	HNRNPH1	5:179614177-179634784	Protein_coding_from_Ensembl	MCF-7 cells	MCF-7 spheroid cells	2.93714	0	-1.97714801	NA	0.04545	0.983395
ENSG00000184007	PTP4A2	1:31842018-31944856	Protein_coding_from_Ensembl	MCF-7 cells	MCF-7 spheroid cells	3.78176	0	-2.25754172	NA	0.047	0.983395
ENSG00000137699	TRIM29	11:120111274-12018552	Protein_coding_from_Ensembl	MCF-7 cells	MCF-7 spheroid cells	26.6671	2.50157	-3.41415	-3.15899	0.00495	0.983395
ENSG00000147813	NAPRT	8:143573489-143603224	Protein_coding_from_Ensembl	MCF-7 cells	MCF-7 spheroid cells	1.33653	0	-1.22436756	NA	0.0497	0.983395
ENSG00000152402	GUCY1A2	11:106674011-10701852	Protein_coding_from_Ensembl	MCF-7 cells	MCF-7 spheroid cells	3.21756	0.320086	-3.32944	-4.26804	0.00085	0.983395
ENSG00000166444	ST5	11:8682410-8910951	Protein_coding_from_Ensembl	MCF-7 cells	MCF-7 spheroid cells	1.06452	0	-1.04580639	NA	0.02525	0.983395
ENSG00000140988	RPS2	16:1962051-1964860	Protein_coding_from_Ensembl	MCF-7 cells	MCF-7 spheroid cells	0	1.85477	1.513374517	NA	0.0185	0.983395
ENSG00000166444	ST5	11:8682410-8910951	Protein_coding_from_Ensembl	MCF-7 cells	MCF-7 spheroid cells	2.51421	0	-1.81320041	NA	0.04285	0.983395
ENSG00000110321	EIF4G2	11:10783312-10809110	Protein_coding_from_Ensembl	MCF-7 cells	MCF-7 spheroid cells	0	5.65232	2.733857569	NA	0.03665	0.983395
ENSG00000023171	GRAMD1I	11:123454397-12362777	Protein_coding_from_Ensembl	MCF-7 cells	MCF-7 spheroid cells	16.6521	40.0129	1.26476	2.0379	0.0408	0.983395
ENSG00000178104	PDE4DIP	1:148808180-149048286	Protein_coding_from_Ensembl	MCF-7 cells	MCF-7 spheroid cells	2.17056	6.03413	1.47508	2.01312	0.04525	0.983395
ENSG00000110321	EIF4G2	11:10783312-10809110	Protein_coding_from_Ensembl	MCF-7 cells	MCF-7 spheroid cells	0	1.61537	1.387015061	NA	0.0421	0.983395
ENSG00000104529	EEF1D	8:143573489-143603224	Protein_coding_from_Ensembl	MCF-7 cells	MCF-7 spheroid cells	2.56963	0	-1.83577454	NA	0.04665	0.983395
ENSG00000166444	ST5	11:8682410-8910951	Protein_coding_from_Ensembl	MCF-7 cells	MCF-7 spheroid cells	1.2599	0	-1.17625894	NA	0.04775	0.983395
ENSG00000104529	EEF1D	8:143573489-143603224	Protein_coding_from_Ensembl	MCF-7 cells	MCF-7 spheroid cells	2.48491	0	-1.8011214	NA	0.0493	0.983395
ENSG00000198382	UVRAG	11:75803430-76143195	Protein_coding_from_Ensembl	MCF-7 cells	MCF-7 spheroid cells	0.000117	2.03135	14.0824	0.062382	0.0287	0.983395
ENSG00000110321	EIF4G2	11:10783312-10809110	Protein_coding_from_Ensembl	MCF-7 cells	MCF-7 spheroid cells	1.37555	0	-1.24826157	NA	0.0371	0.983395
ENSG00000117054	ACADM	1:75724346-75795079	Protein_coding_from_Ensembl	MCF-7 cells	MCF-7 spheroid cells	0	1.206	1.141432791	NA	0.04405	0.983395
ENSG00000009307	CSDE1	1:114716912-114758676	Protein_coding_from_Ensembl	MCF-7 cells	MCF-7 spheroid cells	0	1.85685	1.514425289	NA	0.0382	0.983395
ENSG00000052841	TTC17	11:43311962-43494933	Protein_coding_from_Ensembl	MCF-7 cells	MCF-7 spheroid cells	0	1.52728	1.337585511	NA	0.04285	0.983395
ENSG00000166407	LMO1	11:8224303-8268716	Protein_coding_from_Ensembl	MCF-7 cells	MCF-7 spheroid cells	2.52142	0	-1.81615731	NA	0.00125	0.983395
ENSG00000109971	HSPA8	11:123057488-12306323	Protein_coding_from_Ensembl	MCF-7 cells	MCF-7 spheroid cells	2.61999	0	-1.85598571	NA	0.02975	0.983395
ENSG00000166012	TAF1D	11:93661638-93814695	Protein_coding_from_Ensembl	MCF-7 cells	MCF-7 spheroid cells	0	3.73895	2.244567439	NA	0.0241	0.983395
ENSG00000162144	CYB561A	11:61299450-61369509	Protein_coding_from_Ensembl	MCF-7 cells	MCF-7 spheroid cells	0	1.843	1.507414099	NA	0.0446	0.983395
ENSG00000149212	SESN3	11:95150538-95234104	Protein_coding_from_Ensembl	MCF-7 cells	MCF-7 spheroid cells	7.54281	1.93808	-1.96047	-3.06427	0.00365	0.983395
ENSG00000110852	CLEC2B	12:9852983-9870136	Protein_coding_from_Ensembl	MCF-7 cells	MCF-7 spheroid cells	0	1.72082	1.444041516	NA	0.00995	0.983395
ENSG00000111639	MRPL51	12:6491885-6531955	Protein_coding_from_Ensembl	MCF-7 cells	MCF-7 spheroid cells	0	3.24181	2.084680001	NA	0.0466	0.983395
ENSG00000150991	UBC	12:124911603-12491736	Protein_coding_from_Ensembl	MCF-7 cells	MCF-7 spheroid cells	0	1.81036	1.490754948	NA	0.0399	0.983395
ENSG00000257103	LSM14A	19:34172503-34229515	Protein_coding_from_Ensembl	MCF-7 cells	MCF-7 spheroid cells	0	4.45877	2.448575912	NA	0.04245	0.983395
ENSG00000168234	TTC39C	18:23992772-24135829	Protein_coding_from_Ensembl	MCF-7 cells	MCF-7 spheroid cells	10.4662	2.55206	-2.036	-2.17624	0.037	0.983395
ENSG00000273802	HIST1H2E	6:26215158-26216692	Protein_coding_from_Ensembl	MCF-7 cells	MCF-7 spheroid cells	88.0087	31.7522	-1.47079	-2.51509	0.01435	0.983395
ENSG00000082458	DLG3	X:70444860-70505490	Protein_coding_from_Ensembl	MCF-7 cells	MCF-7 spheroid cells	5.30882	1.01622	-2.38518	-2.70354	0.0128	0.983395
ENSG00000173262	SLC2A14	12:7812511-7900140	Protein_coding_from_Ensembl	MCF-7 cells	MCF-7 spheroid cells	6.66557	19.8658	1.57548	2.28405	0.02275	0.983395
ENSG00000139629	GALNT6	12:51351246-51515763	Protein_coding_from_Ensembl	MCF-7 cells	MCF-7 spheroid cells	12.6251	4.9128	-1.36168	-2.12349	0.0326	0.983395
ENSG00000146242	TPBG	6:82363205-82370828	Protein_coding_from_Ensembl	MCF-7 cells	MCF-7 spheroid cells	11.0531	31.6016	1.51555	2.14658	0.03065	0.983395
ENSG00000130479	MAP1S	19:17719241-17734516	Protein_coding_from_Ensembl	MCF-7 cells	MCF-7 spheroid cells	1.1629	0.000118	-13.2661	-0.06391	0.0446	0.983395
ENSG00000168003	SLC3A2	11:62856101-62888875	Protein_coding_from_Ensembl	MCF-7 cells	MCF-7 spheroid cells	1.44947	0	-1.29246962	NA	0.04445	0.983395
ENSG00000060237	WNK1	12:752592-911452	Protein_coding_from_Ensembl	MCF-7 cells	MCF-7 spheroid cells	5.12576	0	-2.61488884	NA	0.0344	0.983395
ENSG00000134333	LDHA	11:18394387-18408425	Protein_coding_from_Ensembl	MCF-7 cells	MCF-7 spheroid cells	1.20606	0	-1.14147203	NA	0.046	0.983395
ENSG00000111642	CHD4	12:6570082-6607476	Protein_coding_from_Ensembl	MCF-7 cells	MCF-7 spheroid cells	4.55591	0	-2.47402323	NA	0.04545	0.983395
ENSG00000111261	MANSC1	12:12326055-12350541	Protein_coding_from_Ensembl	MCF-7 cells	MCF-7 spheroid cells	0.573402	6.77168	3.5619	3.3175	0.01515	0.983395
ENSG00000112096	SOD2	6:159669056-159789749	Protein_coding_from_Ensembl	MCF-7 cells	MCF-7 spheroid cells	32.2701	341.868	3.40517	3.1765	0.00435	0.983395
ENSG00000134294	SLC38A2	12:46358188-46373778	Protein_coding_from_Ensembl	MCF-7 cells	MCF-7 spheroid cells	0	3.97676	2.315206816	NA	0.02665	0.983395
ENSG00000089157	RPLP0	12:120196685-12020123	Protein_coding_from_Ensembl	MCF-7 cells	MCF-7 spheroid cells	0	2.56547	1.834092265	NA	0.0339	0.983395
ENSG00000111077	TNS2	12:53006157-53079420	Protein_coding_from_Ensembl	MCF-7 cells	MCF-7 spheroid cells	1.40097	0	-1.26361738	NA	0.0417	0.983395

ENSG00000123416	TUBA1B	12:49127781-49147869	Protein_coding_from_Ensembl	MCF-7 cells	MCF-7 spheroid cells	0	2.82975	1.937250218	NA	0.0379	0.983395
ENSG00000100644	HIF1A	14:61681040-61796428	Protein_coding_from_Ensembl	MCF-7 cells	MCF-7 spheroid cells	0	2.42749	1.777152457	NA	0.031	0.983395
ENSG00000258818	RNASE4	14:20684099-20707120	Protein_coding_from_Ensembl	MCF-7 cells	MCF-7 spheroid cells	0.000272	2.2401	13.0078	0.052284	0.04085	0.983395
ENSG00000165861	ZFYVE1	14:72969450-73027212	Protein_coding_from_Ensembl	MCF-7 cells	MCF-7 spheroid cells	0.000146	1.25186	13.0649	0.104586	0.04135	0.983395
ENSG00000092199	HNRNPC	14:21209135-21269494	Protein_coding_from_Ensembl	MCF-7 cells	MCF-7 spheroid cells	5.52634	0	-2.70627415	NA	0.04795	0.983395
ENSG00000092199	HNRNPC	14:21209135-21269494	Protein_coding_from_Ensembl	MCF-7 cells	MCF-7 spheroid cells	1.86101	0	-1.51652454	NA	0.03955	0.983395
ENSG00000166710	B2M	15:44711476-44718877	Protein_coding_from_Ensembl	MCF-7 cells	MCF-7 spheroid cells	2.48662	0	-1.80182914	NA	0.04465	0.983395
ENSG00000140391	TSPAN3	15:77041403-77083984	Protein_coding_from_Ensembl	MCF-7 cells	MCF-7 spheroid cells	8.99535	0	-3.32125709	NA	0.02935	0.983395
ENSG00000140451	PIF1	15:64815631-64825668	Protein_coding_from_Ensembl	MCF-7 cells	MCF-7 spheroid cells	0.000107	1.01976	13.2194	0.044923	0.0433	0.983395
ENSG00000100767	PAPLN	14:73237496-73274640	Protein_coding_from_Ensembl	MCF-7 cells	MCF-7 spheroid cells	5.96586	1.18955	-2.32631	-2.17745	0.0457	0.983395
ENSG00000182774	RPS17	15:82536752-82709914	Protein_coding_from_Ensembl	MCF-7 cells	MCF-7 spheroid cells	0	1.54395	1.347070315	NA	0.03495	0.983395
ENSG00000180209	MYLPF	16:30370933-30377991	Protein_coding_from_Ensembl	MCF-7 cells	MCF-7 spheroid cells	2.07861	0	-1.62227912	NA	0.01215	0.983395
ENSG00000067225	PKM	15:72199028-72231822	Protein_coding_from_Ensembl	MCF-7 cells	MCF-7 spheroid cells	0	2.3936	1.762816526	NA	0.03125	0.983395
ENSG00000108518	PFN1	17:4945651-4957131	Protein_coding_from_Ensembl	MCF-7 cells	MCF-7 spheroid cells	0	2.70329	1.88880753	NA	0.04865	0.983395
ENSG00000132507	EIF5A	17:7306998-7329393	Protein_coding_from_Ensembl	MCF-7 cells	MCF-7 spheroid cells	0	1.24622	1.167499236	NA	0.048	0.983395
ENSG00000129197	RPAIN	17:5282264-5469060	Protein_coding_from_Ensembl	MCF-7 cells	MCF-7 spheroid cells	0	1.32843	1.219357511	NA	0.0459	0.983395
ENSG00000184009	ACTG1	17:81509970-81527776	Protein_coding_from_Ensembl	MCF-7 cells	MCF-7 spheroid cells	1.17115	0	-1.1184594	NA	0.03785	0.983395
ENSG00000258890	CEP95	17:64499615-64662068	Protein_coding_from_Ensembl	MCF-7 cells	MCF-7 spheroid cells	0	3.29978	2.104262846	NA	0.04885	0.983395
ENSG00000161960	EIF4A1	17:7561874-7633383	Protein_coding_from_Ensembl	MCF-7 cells	MCF-7 spheroid cells	0	1.75363	1.46133472	NA	0.03745	0.983395
ENSG00000141367	CLTC	17:59618552-59842255	Protein_coding_from_Ensembl	MCF-7 cells	MCF-7 spheroid cells	2.2032	0	-1.67951388	NA	0.034	0.983395
ENSG00000161956	SENP3	17:7561874-7633383	Protein_coding_from_Ensembl	MCF-7 cells	MCF-7 spheroid cells	2.83673	0	-1.93987724	NA	0.037	0.983395
ENSG00000264608	RP11-192H	17:28455751-28645454	OtherRNA_from_Ensembl	MCF-7 cells	MCF-7 spheroid cells	0	2.7256	1.897472787	NA	0.04855	0.983395
ENSG00000161960	EIF4A1	17:7561874-7633383	Protein_coding_from_Ensembl	MCF-7 cells	MCF-7 spheroid cells	0	5.45537	2.690499788	NA	0.03505	0.983395
ENSG00000161960	EIF4A1	17:7561874-7633383	Protein_coding_from_Ensembl	MCF-7 cells	MCF-7 spheroid cells	0	1.57813	1.366325012	NA	0.0498	0.983395
ENSG00000265190	ANXA8	10:47460161-47484158	Protein_coding_from_Ensembl	MCF-7 cells	MCF-7 spheroid cells	9.65172	0.624114	-3.9509	-3.34029	0.02495	0.983395
ENSG00000089639	GMIP	19:19629475-19643667	Protein_coding_from_Ensembl	MCF-7 cells	MCF-7 spheroid cells	3.26E-05	0.581587	14.1225	0.016453	0.0449	0.983395
ENSG00000127452	FBXL12	19:9810266-9827816	Protein_coding_from_Ensembl	MCF-7 cells	MCF-7 spheroid cells	0.000103	2.0047	14.2458	0.031118	0.0233	0.983395
ENSG00000175387	SMAD2	18:47808956-47931146	Protein_coding_from_Ensembl	MCF-7 cells	MCF-7 spheroid cells	5.50815	0	-2.7022475	NA	0.04775	0.983395
ENSG00000267088	RP11-115N	18:42089311-42089752	OtherRNA_from_Ensembl	MCF-7 cells	MCF-7 spheroid cells	0	1.27345	1.184883275	NA	0.01195	0.983395
ENSG00000167460	TPM4	19:16067020-16103005	Protein_coding_from_Ensembl	MCF-7 cells	MCF-7 spheroid cells	0	1.55119	1.351170348	NA	0.046	0.983395
ENSG00000160570	DEDD2	19:42198597-42220140	Protein_coding_from_Ensembl	MCF-7 cells	MCF-7 spheroid cells	7.80328	22.9183	1.55435	2.16023	0.03425	0.983395
ENSG00000167658	EEF2	19:3976055-3985469	Protein_coding_from_Ensembl	MCF-7 cells	MCF-7 spheroid cells	0	15.9543	4.083579315	NA	0.03635	0.983395
ENSG00000128016	ZFP36	19:39406812-39409412	Protein_coding_from_Ensembl	MCF-7 cells	MCF-7 spheroid cells	3.40215	22.5959	2.73154	3.5715	0.00325	0.983395
ENSG00000167658	EEF2	19:3976055-3985469	Protein_coding_from_Ensembl	MCF-7 cells	MCF-7 spheroid cells	0	2.19374	1.675246869	NA	0.03015	0.983395
ENSG00000119574	ZBTB45	19:58513529-58538911	Protein_coding_from_Ensembl	MCF-7 cells	MCF-7 spheroid cells	4.48E-05	0.738967	14.0111	0.015796	0.0471	0.983395
ENSG00000139289	PHLDA1	12:76025446-76033932	Protein_coding_from_Ensembl	MCF-7 cells	MCF-7 spheroid cells	1.09121	34.8989	4.99918	6.04444	5.00E-05	0.174136
ENSG00000120694	HSPH1	13:31134973-31162388	Protein_coding_from_Ensembl	MCF-7 cells	MCF-7 spheroid cells	9.33481	41.4069	2.14918	2.25755	0.03325	0.983395
ENSG00000271503	CCL5	17:35868966-35885863	Protein_coding_from_Ensembl	MCF-7 cells	MCF-7 spheroid cells	42.5852	108.196	1.34522	2.30824	0.02125	0.983395
ENSG00000204389	HSPA1A	6:31815463-31817946	Protein_coding_from_Ensembl	MCF-7 cells	MCF-7 spheroid cells	18.6013	148.794	2.99984	3.43105	0.0018	0.983395
ENSG00000172379	ARNT2	15:80404349-80597937	Protein_coding_from_Ensembl	MCF-7 cells	MCF-7 spheroid cells	1.13467	15.0353	3.728	4.5279	0.00015	0.335834
ENSG00000156508	EEF1A1	6:73515749-73570596	Protein_coding_from_Ensembl	MCF-7 cells	MCF-7 spheroid cells	5.55927	0	-2.71353526	NA	0.01655	0.983395
ENSG00000278771	Metazoa_S	14:49853615-49853914	OtherRNA_from_Ensembl	MCF-7 cells	MCF-7 spheroid cells	64.0354	206.362	1.68823	2.31702	0.022	0.983395
ENSG00000137834	SMAD6	15:66702227-66782848	Protein_coding_from_Ensembl	MCF-7 cells	MCF-7 spheroid cells	7.28906	1.76596	-2.04528	-2.41825	0.023	0.983395
ENSG00000108819	PPP1R9B	17:50133734-50150630	Protein_coding_from_Ensembl	MCF-7 cells	MCF-7 spheroid cells	33.6722	14.0598	-1.25998	-2.16172	0.03505	0.983395
ENSG00000145242	EPHA5	4:65319562-65698029	Protein_coding_from_Ensembl	MCF-7 cells	MCF-7 spheroid cells	3.1484	0.439747	-2.83987	-2.52595	0.03415	0.983395
ENSG00000154511	FAM69A	1:92832024-92961522	Protein_coding_from_Ensembl	MCF-7 cells	MCF-7 spheroid cells	1.05585	0	-1.03973501	NA	0.0258	0.983395
ENSG00000273983	HIST1H3C	6:26269404-26271815	Protein_coding_from_Ensembl	MCF-7 cells	MCF-7 spheroid cells	146.736	46.6223	-1.65414	-2.89793	0.0058	0.983395
ENSG00000276966	HIST1H4E	6:26204551-26206038	Protein_coding_from_Ensembl	MCF-7 cells	MCF-7 spheroid cells	340.534	114.101	-1.57748	-2.78192	0.0082	0.983395
ENSG00000276180	HIST1H4I	6:27138587-27139881	Protein_coding_from_Ensembl	MCF-7 cells	MCF-7 spheroid cells	153.482	39.2462	-1.96745	-3.39081	0.0017	0.983395
ENSG00000261150	EPPK1	8:143857323-143878464	Protein_coding_from_Ensembl	MCF-7 cells	MCF-7 spheroid cells	14.5543	4.56446	-1.67292	-2.8332	0.00545	0.983395
ENSG00000081692	JMJD4	1:227728538-227781231	Protein_coding_from_Ensembl	MCF-7 cells	MCF-7 spheroid cells	0.000251	2.89592	13.4935	0.103976	0.0454	0.983395
ENSG00000102125	TAZ	X:154389954-154421726	Protein_coding_from_Ensembl	MCF-7 cells	MCF-7 spheroid cells	1.08991	0	-1.06344082	NA	0.03345	0.983395
ENSG00000274641	HIST1H2B	6:27893462-27893843	Protein_coding_from_Ensembl	MCF-7 cells	MCF-7 spheroid cells	402.862	170.83	-1.23773	-2.1027	0.03795	0.983395
ENSG00000162496	DHRS3	1:12567909-12617731	Protein_coding_from_Ensembl	MCF-7 cells	MCF-7 spheroid cells	35.605	9.16188	-1.95836	-2.92088	0.0069	0.983395
ENSG00000173702	MUC13	3:124905441-124953819	Protein_coding_from_Ensembl	MCF-7 cells	MCF-7 spheroid cells	0.097093	2.24094	4.52858	3.672	0.0424	0.983395
ENSG00000178917	ZNF852	3:44498969-44510640	Protein_coding_from_Ensembl	MCF-7 cells	MCF-7 spheroid cells	7.04E-05	1.226	14.088	0.019884	0.0182	0.983395
ENSG00000169499	PLEKHA2	8:38901234-38973909	Protein_coding_from_Ensembl	MCF-7 cells	MCF-7 spheroid cells	15.0168	4.86799	-1.62518	-2.5892	0.0104	0.983395
ENSG00000197584	KCNMB2	3:178202049-178860405	Protein_coding_from_Ensembl	MCF-7 cells	MCF-7 spheroid cells	0.000241	0.55946	11.1823	0.069699	0.0271	0.983395
ENSG00000182919	C11orf54	11:93661638-93814695	Protein_coding_from_Ensembl	MCF-7 cells	MCF-7 spheroid cells	0	1.70468	1.435457914	NA	0.04005	0.983395
ENSG00000174938	SEZ6L2	16:29871158-29899547	Protein_coding_from_Ensembl	MCF-7 cells	MCF-7 spheroid cells	1.2494	5.24013	2.06836	2.25541	0.0421	0.983395
ENSG00000237412	PRSS56	2:232520462-232525716	Protein_coding_from_Ensembl	MCF-7 cells	MCF-7 spheroid cells	82.9341	4.55672	-4.1859	-4.79377	0.00035	0.609476
ENSG00000128965	CHAC1	15:40952961-40956519	Protein_coding_from_Ensembl	MCF-7 cells	MCF-7 spheroid cells	26.7689	10.1692	-1.39635	-2.12762	0.02855	0.983395
ENSG00000277775	HIST1H3F	6:26250194-26250605	Protein_coding_from_Ensembl	MCF-7 cells	MCF-7 spheroid cells	445.949	135.548	-1.71808	-2.90212	0.00425	0.983395
ENSG00000183049	CAMK1D	10:12349481-12835545	Protein_coding_from_Ensembl	MCF-7 cells	MCF-7 spheroid cells	5.1207	0.787193	-2.70155	-3.51519	0.0035	0.983395
ENSG00000275713	HIST1H2B	6:26251650-26253710	Protein_coding_from_Ensembl	MCF-7 cells	MCF-7 spheroid cells	50.8418	11.3922	-2.15797	-3.56057	0.00055	0.801836
ENSG00000111877	MCM9	6:118813441-119149387	Protein_coding_from_Ensembl	MCF-7 cells	MCF-7 spheroid cells	0.935763	0.000198	-12.2059	-0.10073	0.04785	0.983395
ENSG00000167508	MVD	16:88651934-88663161	Protein_coding_from_Ensembl	MCF-7 cells	MCF-7 spheroid cells	5.63781	22.4543	1.99379	2.32753	0.03005	0.983395
ENSG00000196890	HIST3H2B	1:228458106-228460470	Protein_coding_from_Ensembl	MCF-7 cells	MCF-7 spheroid cells	6.02936	1.60508	-1.90936	-2.31729	0.02665	0.983395
ENSG00000233276	GPX1	3:49357175-49358600	Protein_coding_from_Ensembl	MCF-7 cells	MCF-7 spheroid cells	82.5417	214.308	1.37649	2.37198	0.02125	0.983395
ENSG00000274267	HIST1H3B	6:26031649-26032060	Protein_coding_from_Ensembl	MCF-7 cells	MCF-7 spheroid cells	1490.93	550.043	-1.4386	-2.54587	0.01325	0.983395
ENSG00000182985	CADM1	11:115169217-11550495	Protein_coding_from_Ensembl	MCF-7 cells	MCF-7 spheroid cells	3.78173	0.751215	-2.33175	-2.24955	0.0426	0.983395
ENSG00000010626	LRRC23	12:6867118-6923698	Protein_coding_from_Ensembl	MCF-7 cells	MCF-7 spheroid cells	0	1.42123	1.275740132	NA	0.0468	0.983395
ENSG00000280335	RP11-15F1	18:49013105-49013819	OtherRNA_from_Ensembl	MCF-7 cells	MCF-7 spheroid cells	1.34927	0	-1.23221253	NA	0.00305	0.983395
ENSG00000151623	NR3C2	4:148078761-148444698	Protein_coding_from_Ensembl	MCF-7 cells	MCF-7 spheroid cells	0.663537	3.46E-05	-14.2287	-0.03578	0.0351	0.983395
ENSG00000135346	CGA	6:87085497-87095406	Protein_coding_from_Ensembl	MCF-7 cells	MCF-7 spheroid cells	0.473541	76.3553	7.33309	6.91006	0.01885	0.983395
ENSG00000280502	Metazoa_S	11:118994333-11899463	OtherRNA_from_Ensembl	MCF-7 cells	MCF-7 spheroid cells	2.18688	0	-1.6721447	NA	0.0189	0.983395
ENSG00000282988	RP1-34B2	6:26195565-26199293	Protein_coding_from_Ensembl	MCF-7 cells	MCF-7 spheroid cells	399.044	117.338	-1.76588	-2.65566	0.00815	0.983395
