# Supplementary material for: Protease Nexin I is a feedback regulator of EGF/PKC/MAPK/EGR1 signaling in breast cancer cells metastasis and stemness
Source: Cell Death Dis. 2019 Sep 9;10(9):649. doi: 10.1038/s41419-019-1882-9 (PMC6733841; doi:10.1038/s41419-019-1882-9)
Supplement: Supplementary file 9 — Supplementary Table S3. [file 41419_2019_1882_MOESM9_ESM.docx]

**Supplementary Table S3. PN-1 expression and clinicopathological features in 70 patients with breast cancer**

| **Characteristics** | **Expression of PN-1** | | **p value*** |
| --- | --- | --- | --- |
|  | **low** | **high** |  |
| **Sex** |  |  |  |
| male | 0 | 0 |  |
| female | 35 | 35 |  |
| **Age** |  |  | 0.232 |
| ≤60 | 26 | 30 |  |
| >60 | 9 | 5 |  |
| **Grade** |  |  | 0.031* |
| Ⅰ/I-II, well-differentiated | 2 | 1 |  |
| Ⅱ/II-III, moderately differentiated | 20 | 10 |  |
| Ⅲ, poorly differentiated | 13 | 24 |  |
| **Tumor histological** |  |  | 0.019* |
| Ductal carcinoma *in situ* | 15 | 6 |  |
| Invasive ductal carcinoma | 20 | 29 |  |
| **T Classification** |  |  | 0.060 |
| T1 | 13 | 5 |  |
| T2 | 16 | 25 |  |
| T3 | 6 | 5 |  |
| **N Classification** |  |  | 0.051 |
| N0 | 26 | 16 |  |
| N1 | 7 | 11 |  |
| N2 | 1 | 7 |  |
| N3 | 1 | 1 |  |
| **ER status** |  |  | 0.000* |
| Negative | 8 | 29 |  |
| Positive | 27 | 6 |  |
| **PR status** |  |  | 0.094 |
| Negative | 13 | 20 |  |
| Positive | 22 | 15 |  |
| **HER2 status** |  |  | 0.000* |
| Negative | 4 | 23 |  |
| Positive | 31 | 12 |  |
| **Tumor size**(cm^3^) |  |  | 0.151 |
| ≤ 6 | 20 | 14 |  |
| > 6 | 15 | 21 |  |
| **Lymph node metastasis** |  |  | 0.017* |
| Negative | 23 | 13 |  |
| Positive | 12 | 22 |  |

Median expression level was used as a cutoff to divide the 70 patients into PN-1 low group (n = 35) and PN-1 high group (n = 35).

Two-sided χ^2^ test. * p<0.05
