# Supplementary material for: Protease Nexin I is a feedback regulator of EGF/PKC/MAPK/EGR1 signaling in breast cancer cells metastasis and stemness
Source: Cell Death Dis. 2019 Sep 9;10(9):649. doi: 10.1038/s41419-019-1882-9 (PMC6733841; doi:10.1038/s41419-019-1882-9)
Supplement: Supplementary file 10 — Supplementary Table S4. [file 41419_2019_1882_MOESM10_ESM.docx]

**Table S4. The primers used in ChIP-qPCR.**

| **Primer Name** | **Forward Primer** | **Reverse Primer** |
| --- | --- | --- |
| **P1** | CGATAAAGCCCCCGCCGCCG | AACCGACGCAGGAGGCTGA |
| **P2** | AGGCGGGCCGGGGGCGGGG | CCCCGGGCGCGCCGCGGCG |
| **P3** | GACACGGACCCCTGGCGGGGCGG | CCGCCGCCCTTCCGCCCCGT |
| **P4** | GTACGAGCCCTGGTATTCC | GCAAGGCCCGCGTCGGGGTG |
| **Neg** | GGCCCCTGGGGTATGTGCCT | AAGGGGAGCAGACATAAACGG |
